# Supplementary material for: The Lassa virus fusion domain has structural plasticity and exploits bis(monoacylglycero)phosphate for fusion
Source: Biophys J. 2026 Feb 13;125(5):1384–402. doi: 10.1016/j.bpj.2026.01.049 (PMC13351859; doi:10.1016/j.bpj.2026.01.049)
Supplement: Document S1. Figures S1–S20 and Tables S1–S8 [file mmc1.pdf]

Biophysical Journal, Volume *125*

Supplemental information

The Lassa virus fusion domain has structural plasticity and exploits bis(monoacylglycero)phosphate for fusion

Hallie N. Pennington, Kiruthika Prahadeesh, Quinn M. Mulvihill, Sungjai Shin, Wonpil Im, and Jinwoo Lee

SUPPORTING INFORMATION

THE LASSA VIRUS FUSION DOMAIN HAS STRUCTURAL PLASTICITY AND EXPLOITS BIS(MONOACYLGLYCERO)PHOSPHATE FOR FUSION

HALLIE N. PENNINGTON[1], KIRUTHIKA PRAHADEESH[1], QUINN M. MULVIHILL[1], SUNGJAI SHIN[1], WONPIL IM[2], AND JINWOO LEE[1]*

[1]Department of Chemistry and Biochemistry, College of Computer, Mathematics, and Natural Science, University of Maryland, College Park, MD, 20740, USA

[2]Department of Biological Sciences, College of Arts and Sciences, Lehigh University, Bethlehem, PA, 18015, USA

*Correspondence: Jinwoo Lee (jinwoo@umd.edu)

Figures	Supporting Information
S1	Structural organization of LASV Glycoprotein complex (GPC) and its viral entry pathway via endocytosis
S2	Structures of purchased lipids
S3	Temperature titration of the LASV FD in the pre- and post-fusion states via solution NMR spectroscopy
S4	Chemical shift indexing of the LASV FD vs BMRB values
S5	Cβ strips for the pre- and post-fusion states of the LASV FD
S6	Thiol assay in the LASV FD under native and reducing conditions
S7	Gd-DTPA titration of the LASV FD in the pre-fusion state
S8	Gd-DTPA and 16-DSA titration of the LASV FD in the post-fusion state
S9	R_1 and R_2 relaxation rates of the LASV FD in the pre- and post-fusion states
S10	^{1}H – ^{15}N NOE shows that the FD becomes reduced in flexibility upon fusion
S11	^{1}H – ^{15}N strips for L^{266} in the pre-fusion state
S12	^{1}H – ^{15}N strips for L^{266} in the post-fusion state
S13	Membrane depth and dynamics of different L^{266} conformations
S14	^{19}F spectra of $F^{293}W$ in the pre- and post-fusion state
S15	Exemplary isotherms of the lysine mutants
S16	Exemplary isotherms of the LASV FD in different lipids

S17	Fusion of the LASV FD in lipids with different tail saturations
S18	Helical graphs of the LASV FD in POPG, BMP, and POPS
S19	Effect of LASV FD on membrane packing
S20	Contact analysis of LASV FD in different anionic lipid membranes

**Tables**	**Supporting Information**
S1	Lipid compositions of the membrane systems generated for MD simulations
S2	TALOS+ statistics for the pre-fusion state
S3	TALOS+ statistics for the post-fusion state
S4	Average relative intensities of LASV FD in different paramagnetic probes
S5	Overall relaxation times of the LASV FD in the pre- and post-fusion state
S6	Populations for residues with multiple conformers in the pre- and post-fusion state
S7	Predicted dihedral angles for different conformers in the pre-fusion state
S8	Predicted dihedral angles for different conformers in the post-fusion state

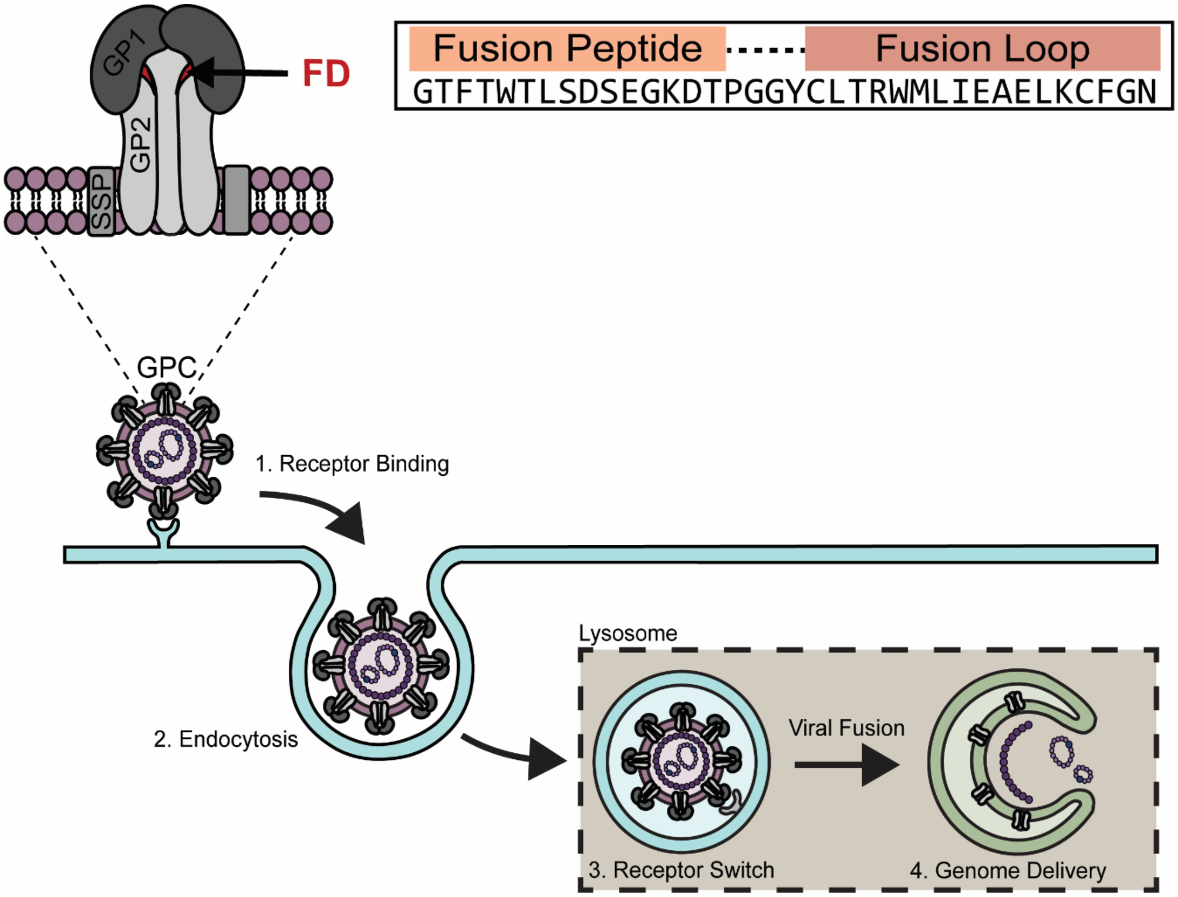

Figure S1: Structural organization of LASV Glycoprotein complex (GPC) and its viral entry pathway via endocytosis. The glycoprotein complex on the Lassa Virus (LASV) surface consists of three components; the receptor binding subunit (GP1), the fusion subunit (GP2), and the stable signal peptide (SSP). Fusion Domain (FD) located at the N-terminus of GP2, is highlighted in red, with its sequence provided to distinguish the fusion peptide (FP) and fusion loop (FL). During the entry, LASV first binds to the host cell receptor through GP1 (Step 1), triggering endocytosis of the virion (Step 2). After internalization, the virion is trafficked to the late endosome/ lysosome where GP1 interact with its secondary receptor, LAMP1 (Step 3). Following GP1 dissociation from GPC, membrane fusion occurs, delivering the viral genome into the host cell cytoplasm (Step 4).

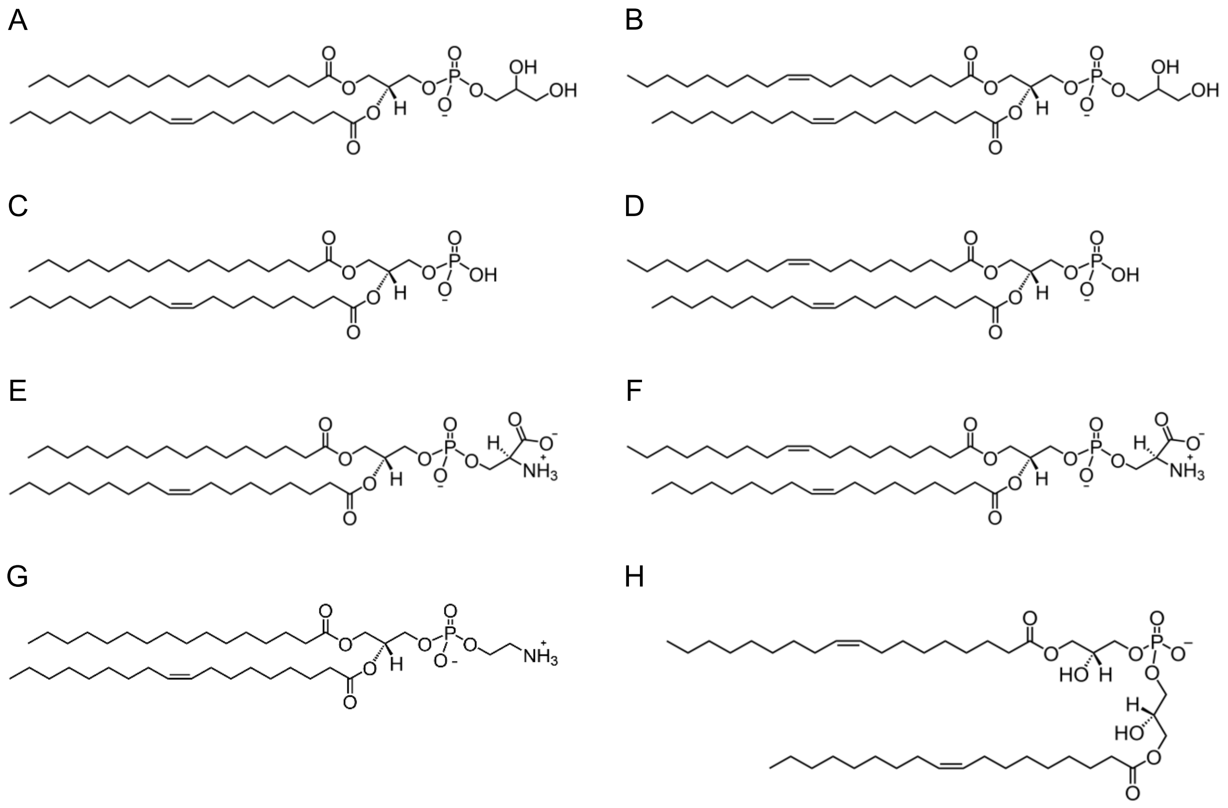

Figure S2. Structure of various lipids employed in this study that were purchased from Avanti Lipids. [A] 16:0–18:1 1-palmitoyl-2-oleoyl-*sn*-glycero-3-phospho-[1′-rac-glycerol] (POPG); [B] 1,2-dioleoyl-*sn*-glycero-3-[phospho-rac-(3-lysyl(1-glycerol))] (DOPG); [C] 1-palmitoyl-2-oleoyl-*sn*-glycero-3-phosphate (POPA); [D] 1,2-dioleoyl-*sn*-glycero-3-phosphate (DOPA); [E] 1-palmitoyl-2-oleoyl-*sn*-glycero-3-phospho-L-serine (POPS); [F] 1,2-dioleoyl-*sn*-glycero-3-phospho-L-serine (DOPS); [G] 1-palmitoyl-2-oleoyl-*sn*-glycero-3-phosphoethanolamine (POPE); and [H] bis(monooleoylglycero)phosphate (S,R Isomer) (BMP). All structures drawn in ChemDraw.

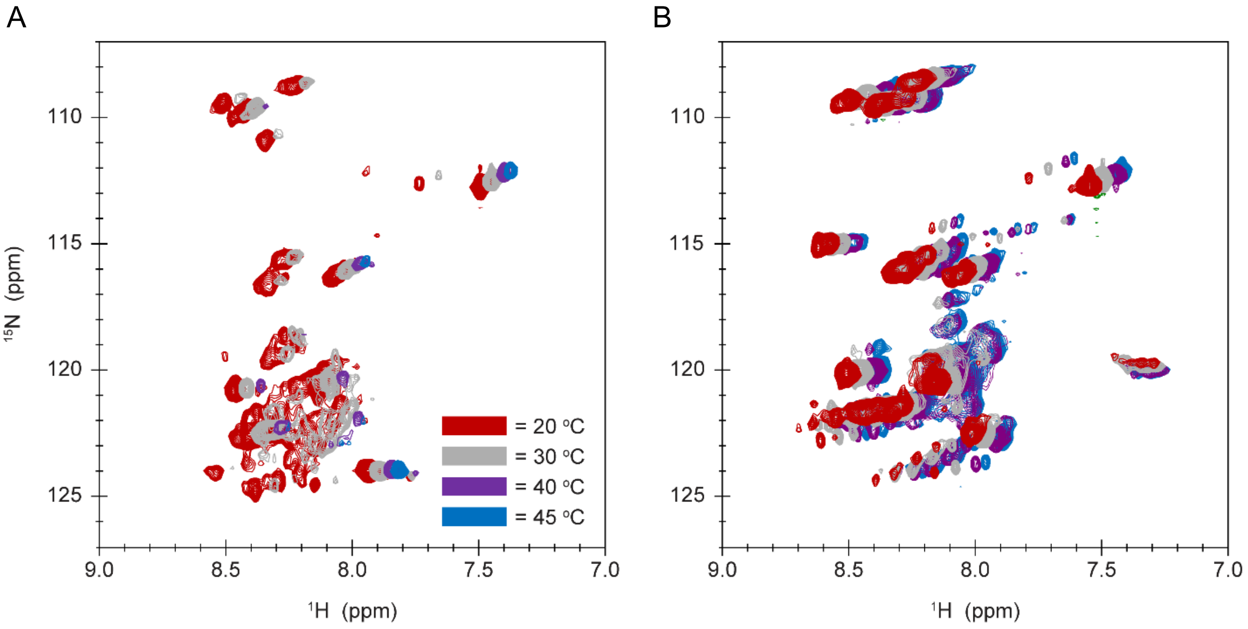

Figure S3. The LASV FD requires a different temperature for the different fusion states to be effectively visualized via NMR spectroscopy. [A,B] An overlay of 1H – 15N HSQC spectra for the [A] pre- and [B] post-fusion state of the LASV FD at 20 ℃ (red), 30 ℃ (grey), 40 ℃ (purple), and 45 ℃ (blue). All measurements were carried out on ~500 μM of the LASV FD in 300 μL of 25 mM Na2HPO4, 100 mM NaCl, pH 7.0 (pre-fusion) or pH 4.0 with acidic bicelles, q = 0.5 (post-fusion).

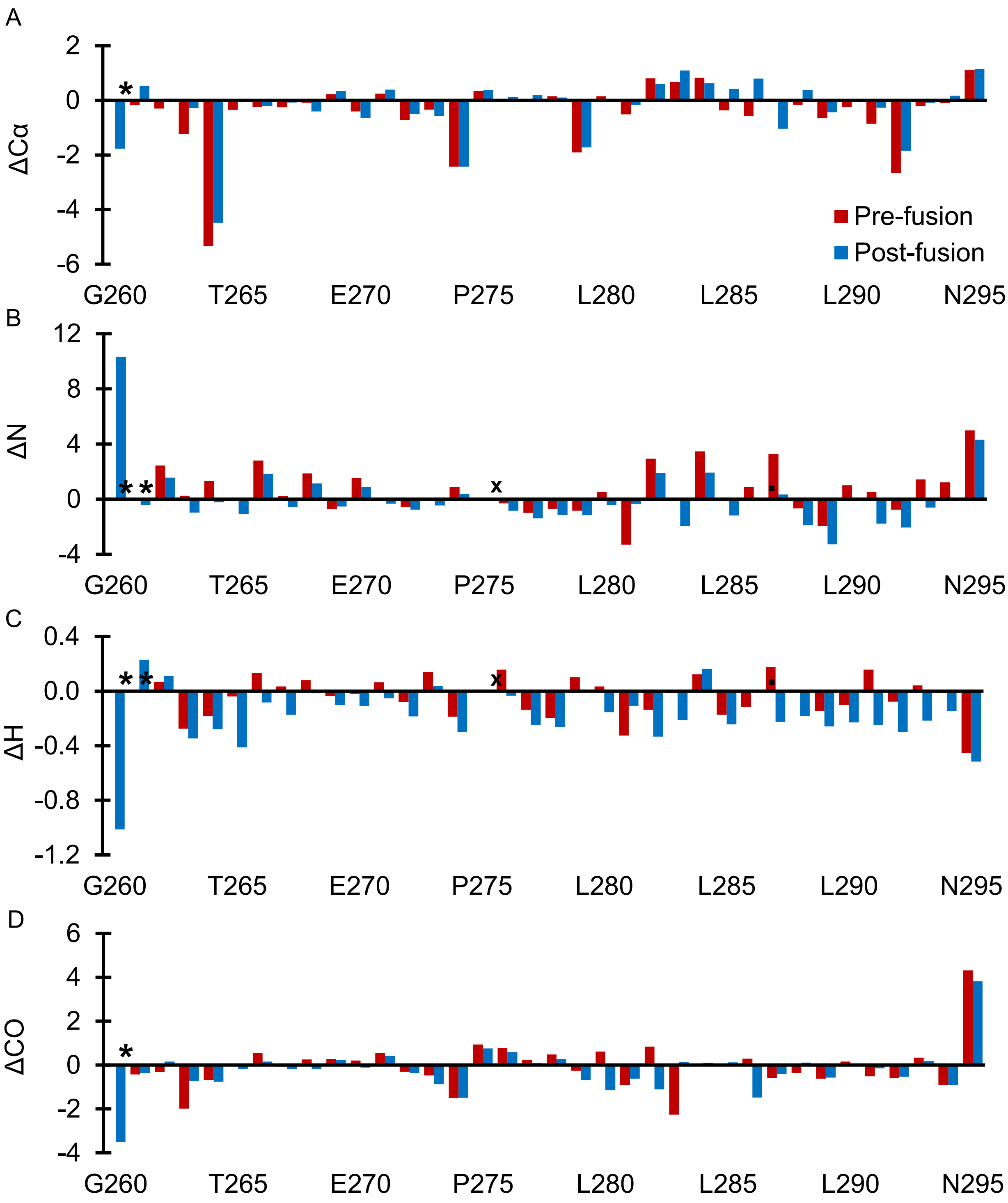

Figure S4. Chemical shift indexing in comparison to backbone atoms does not reveal information regarding the secondary structure of the LASV FD. [A] ΔCα [B] ΔN; [C] ΔH; and [D] ΔCO. All measurements were carried out on ~500 μM of the LASV FD in 300 μL of 25 mM Na2HPO4, 100 mM NaCl, pH 7.0 at 20 ℃ (pre-fusion) or pH 4.0 with acidic bicelles, q = 0.5 at 45 ℃ (post-fusion). Proline residues and other residues that could not be assigned for both states are marked (X), whereas residues that could not be assigned for the pre- or post-fusion state are marked with an asterisk (*) or period (.), accordingly.

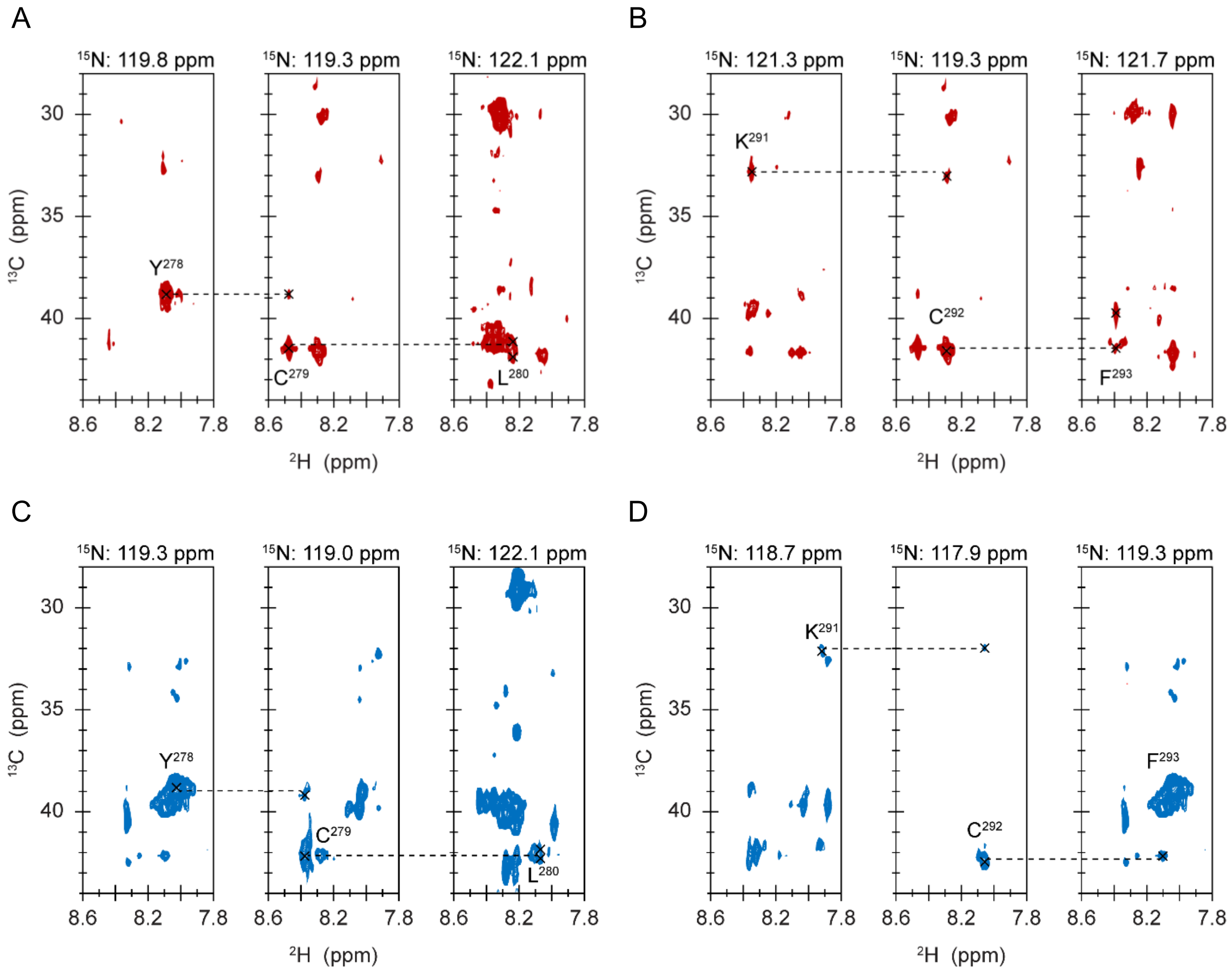

Figure S5. Cβ shifts confirm the presence of an internal disulfide bond within the FL of the LASV FD in both the pre- and post-fusion states. [A] Pre-fusion C279; [B] Pre-fusion C292; [C] Post-fusion C279; and [D] Post-fusion C292 all have 13Cβ chemical shifts greater than 35 ppm, indicative that the cysteine residues were oxidized. All data were acquired from HN(CA)CB experiments with 650 μM of a triple labelled sample (2H/13C/15N) in 25 mM Na2HPO4, 100 mM NaCl, pH 7.0 at 20 ℃ [A, B] or pH 4.0 at 45 ℃ with acidic bicelles, q = 0.5 [C,D].

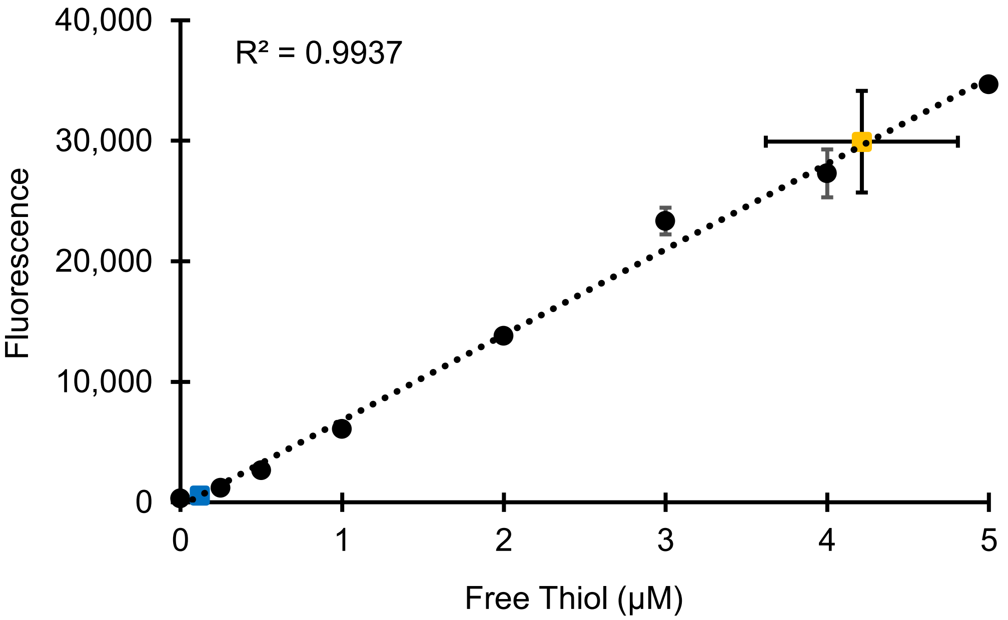

Figure S6. The LASV FD has a properly formed disulfide bond. Under native conditions (blue, square; n = 3), the LASV FD showed that the disulfide bond was properly formed. Upon introduction of 1 mM TCEP to the FD (yellow, square; n = 2), there was a large increase in the concentration of free thiol present, indicative that the disulfide bond was disrupted. Assay was performed according to the manufacturer's protocol with standards in duplicates. Standard curve (black, circle) was generated to determine the unknown thiol concentration. The average fluorescence of 1 mM TCEP was run and subtracted from the average fluorescence of the FD with 1 mM TCEP. Errors were propagated accordingly from the standard deviations.

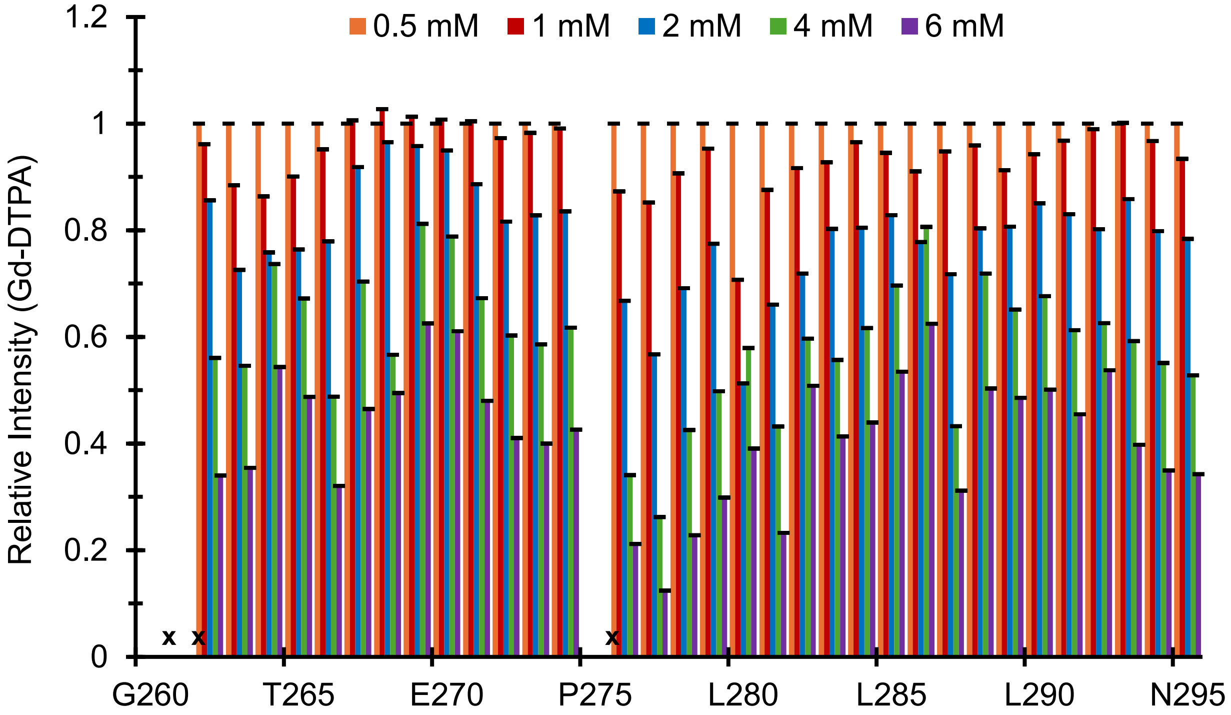

Figure S7. The LASV FD is solvent-exposed in the pre-fusion state. Progressive titration of the water-soluble, paramagnetic agent Gd-DTPA in increments of 0.5 mM (orange), 1 mM (red), 2 mM (blue), 4 mM (green), and 6 mM (blue) into the LASV FD revealed that all residues experience virtually the same amount of quenching. The relative intensity prior to titration is not shown due to increasing signals from the paramagnetic effect. Data normalized to 0.5 mM Gd-DTPA for each titration. Error bars shown are propagated from the signal-to-noise ratio and standard error of the mean (SEM). All measurements were carried out on ~500 μM of the LASV FD in 300 μL of 25 mM Na2HPO4, 100 mM NaCl, pH 7.0 at 20 ℃. Proline residues and other residues that could not be assigned are marked (X).

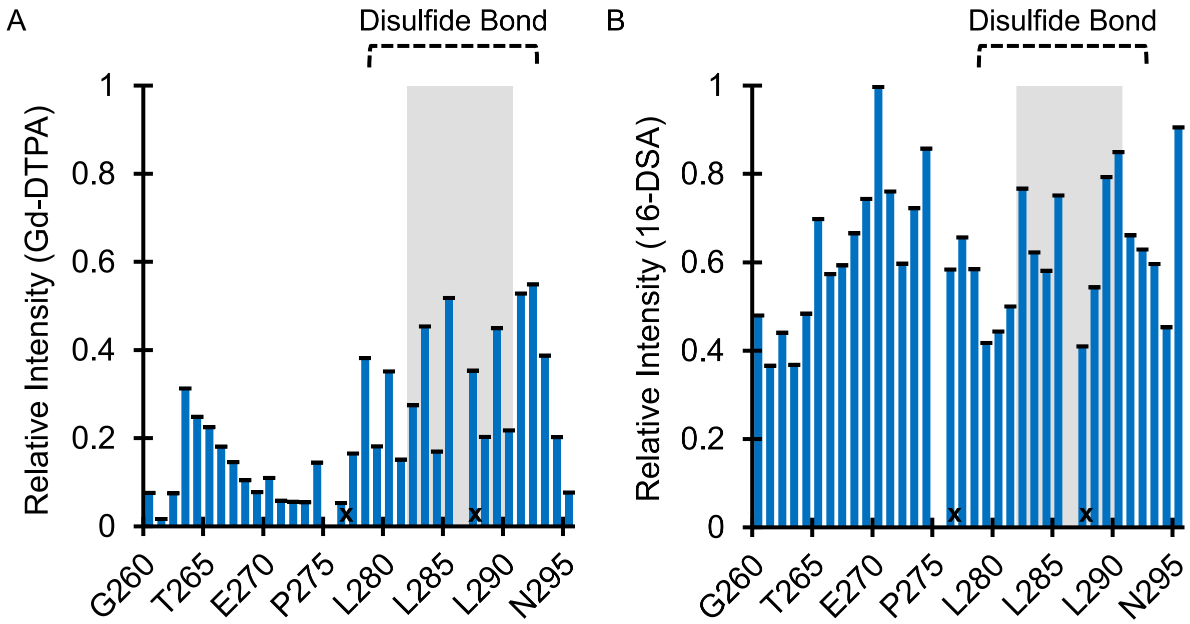

Figure S8. In the post-fusion state, the LASV FD associates with the membrane in a shallow manner via its FL while the FP remains solvent-exposed. [A] Introduction of 6 mM Gd-DTPA into the system containing the LASV FD resulted in significant quenching of the LASV FP, particularly D268 – T274, but not the FL. Titration of 6 mM 16-DSA into the LASV resulted in nearly identical quenching for both the FP and FL. For all experiments, the relative intensity at 0.5 mM of a given probe was subtracted due to increasing signals. All error bars shown are propagated from the signal-to-noise ratio and standard error of the mean (SEM). All measurements were carried out on ~500 μM of the LASV FD in 300 μL of 25 mM Na2HPO4, 100 mM NaCl, pH 4.0 with acidic bicelles, q = 0.5 at 45 ℃. The disulfide bond (C^{279} and C^{292}, dashed line) and helix (R^{282} – L^{290}, transparent grey box) are indicated. Proline residues and other residues that could not be assigned are marked (X).

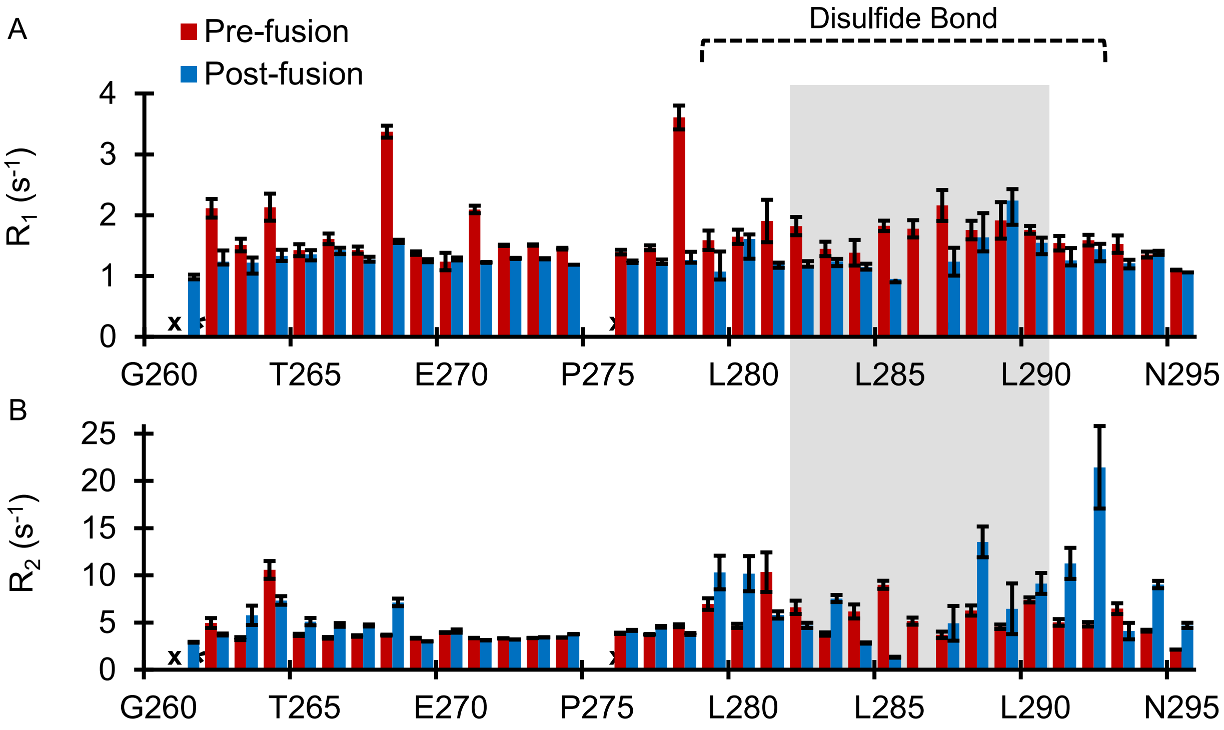

Figure S9. Dynamic properties were slightly altered in several regions within the LASV FD from the pre- (red) to post-fusion (blue) state. [A] Both the FP and FL have similar R_1 relaxation rates in the pre-fusion state that were similar but decreased in the post-fusion state. [B] R_2 relaxation rates for the FP were similar between the pre- and post-fusion states but increased for the FL in the post-fusion state. Error bars shown are propagated from the signal-to-noise ratio. All measurements were carried out on ~500 μM of the LASV FD in 300 μL of 25 mM Na_2HPO_4, 100 mM NaCl, pH 7.0 at 20 ℃ (pre-fusion) or pH 4.0 with acidic bicelles, q = 0.5 at 45 ℃ (post-fusion). The disulfide bond (C^{279} and C^{292}, dashed line) and helix (R^{282} – L^{290}, transparent grey box) are indicated. Proline residues and other residues that could not be assigned for both states are marked (X), whereas residues that could not be assigned for the pre- or post-fusion state are marked with an asterisk (*) or period (.), accordingly.

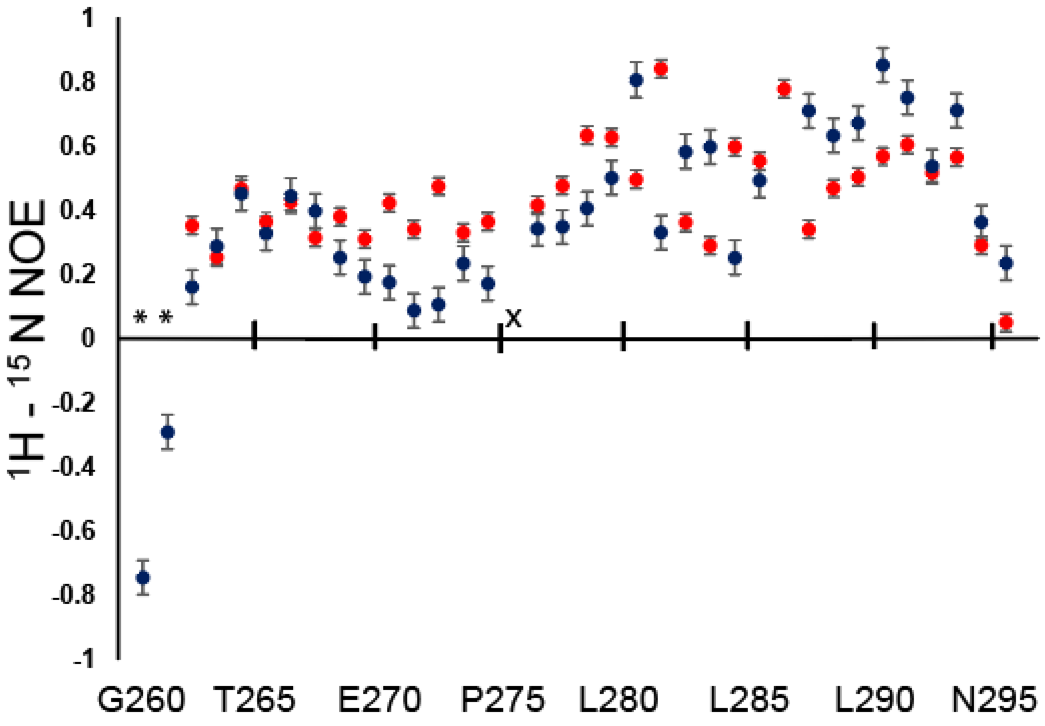

Figure S10. ^{1}H – ^{15}N NOE shows that the FD becomes reduced in flexibility upon fusion (Prefusion-red and postfusion-blue). All measurements were carried out on ~500 μM of the LASV FD in 300 μL of 25 mM Na_2HPO_4, 100 mM NaCl, pH 7.0 at 20 ℃ (pre-fusion) or pH 4.0 with acidic bicelles, q = 0.5 at 45 ℃ (post-fusion). Proline residues and other residues that could not be assigned for both states are marked with an x, whereas residues that could not be assigned for the pre- or post-fusion state are marked with an asterisk (*).

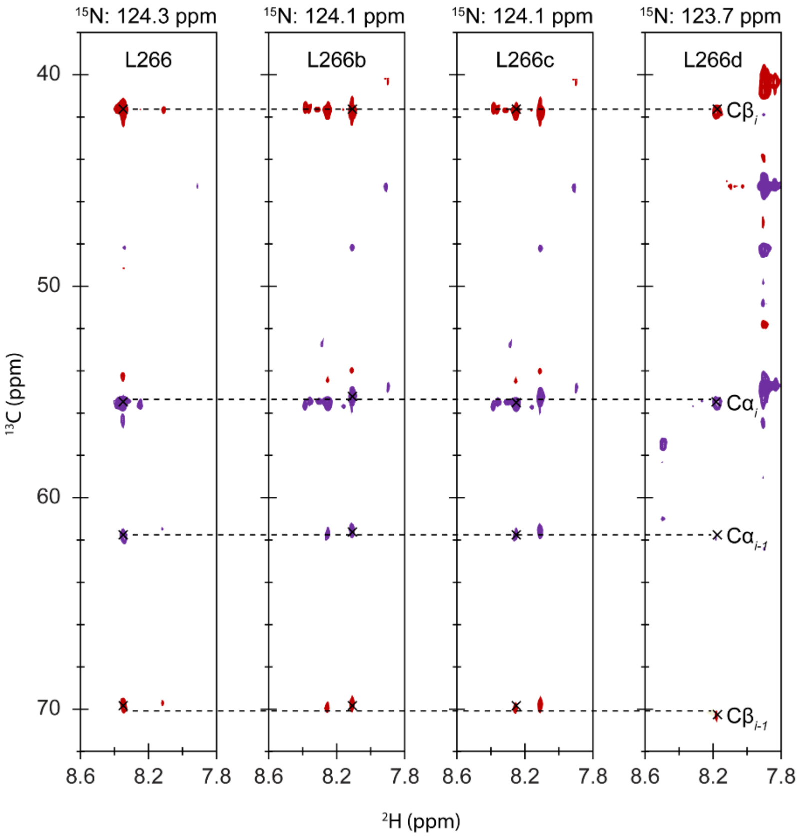

Figure S11. Multiple conformations for L^{266} were observed in the pre-fusion states. Four different ^{1}H – ^{15}N strips had the same Cα (purple) and Cβ (red) shifts for the *i* and *i – 1,* corresponding to L^{266} and T^{265}, accordingly. Data was acquired from HN(CA)CB experiments with 650 μM of a triple labelled sample (^{2}H/^{13}C/^{15}N) in 25 mM Na_2HPO_4, 100 mM NaCl, pH 7.0 at 20 ℃. The most populated conformer (a) is the residue label, whereas the second (b), third (c), and fourth (d) most populated conformers are indicated as such.

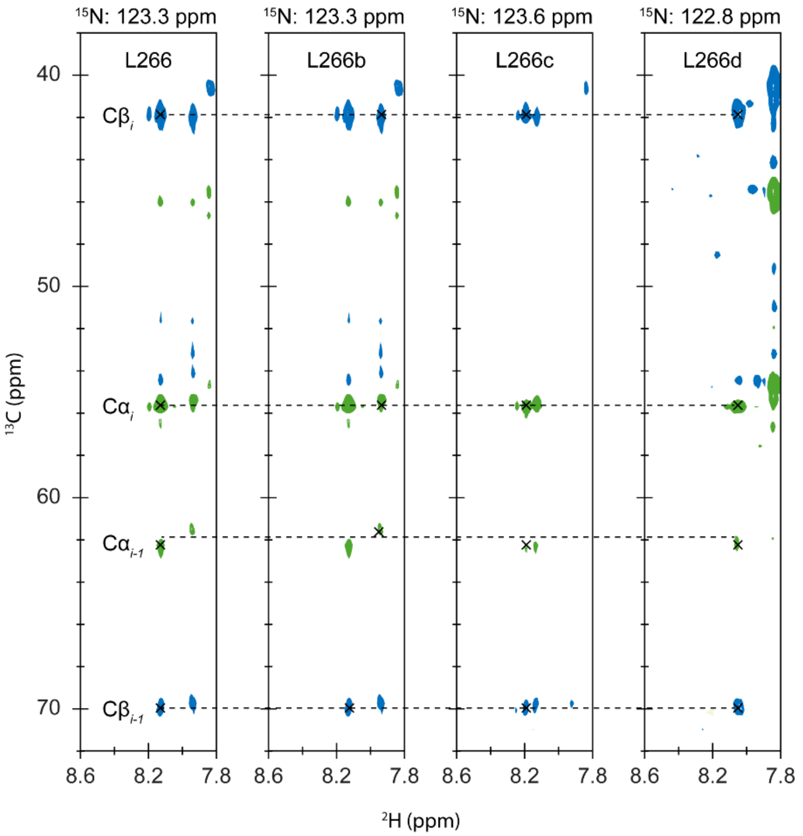

Figure S12. L266 has several conformers in the post-fusion state. Different ^{15}N strips with the same Cα (green) and Cβ (blue) shifts were observed in the post-fusion state for L266. All data were acquired from HN(CA)CB experiments with 650 μM of a triple labelled sample ($^{2}H/^{13}C/^{15}N$) in 25 mM Na_2HPO_4, 100 mM NaCl, pH 4.0 at 45 ℃ with acidic bicelles, q =0.5. The most populated conformer (a) is the residue label, whereas the second (b), third (c), and fourth (d) most populated conformers are indicated as such.

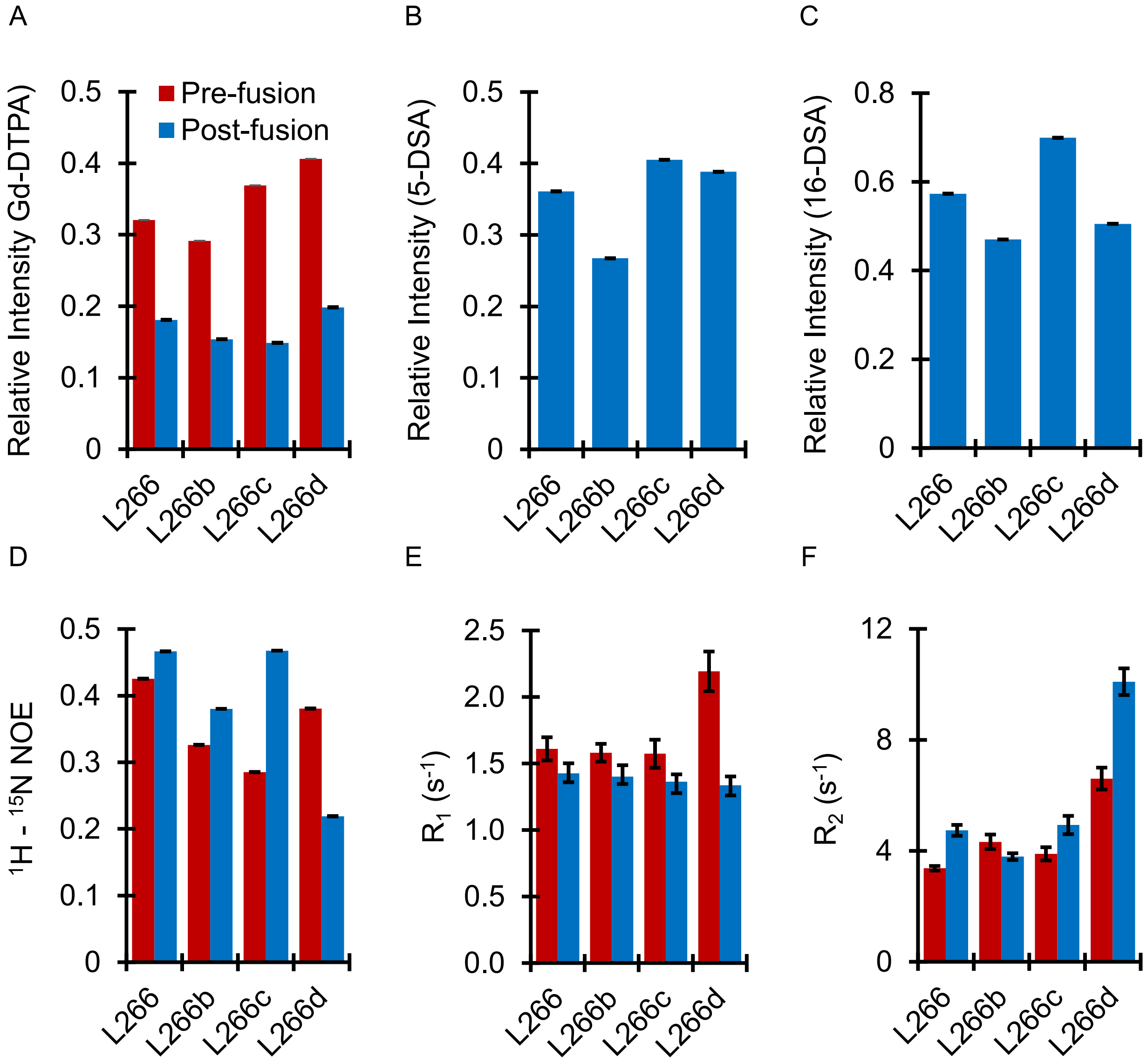

Figure S13. Membrane depth and dynamics of L^{266} are relatively similar between the different conformations. [A-C] Each conformer of L^{266} experiences comparable amounts of quenching in the presence of [A] 6 mM Gd-DTPA, [B] 8 mM 5-DSA, and [C] 6 mM 16-DSA. [D-E] The different L^{266} populations have similar dynamics as revealed by [D] ^{1}H – ^{15}N NOE, [E] R_1 relaxation, and [F] R_2 relaxation experiments. Error bars shown are propagated from the signal-to-noise ratio. All measurements were carried out on ~500 μM of the LASV FD in 300 μL of 25 mM Na_2HPO_4, 100 mM NaCl, pH 7.0 at 20 ℃ (pre-fusion, red) or pH 4.0 with acidic bicelles, q = 0.5 at 45 ℃ (post-fusion, blue). The second (b), third (c), and fourth (d) most populated conformers of a given residue are indicated.

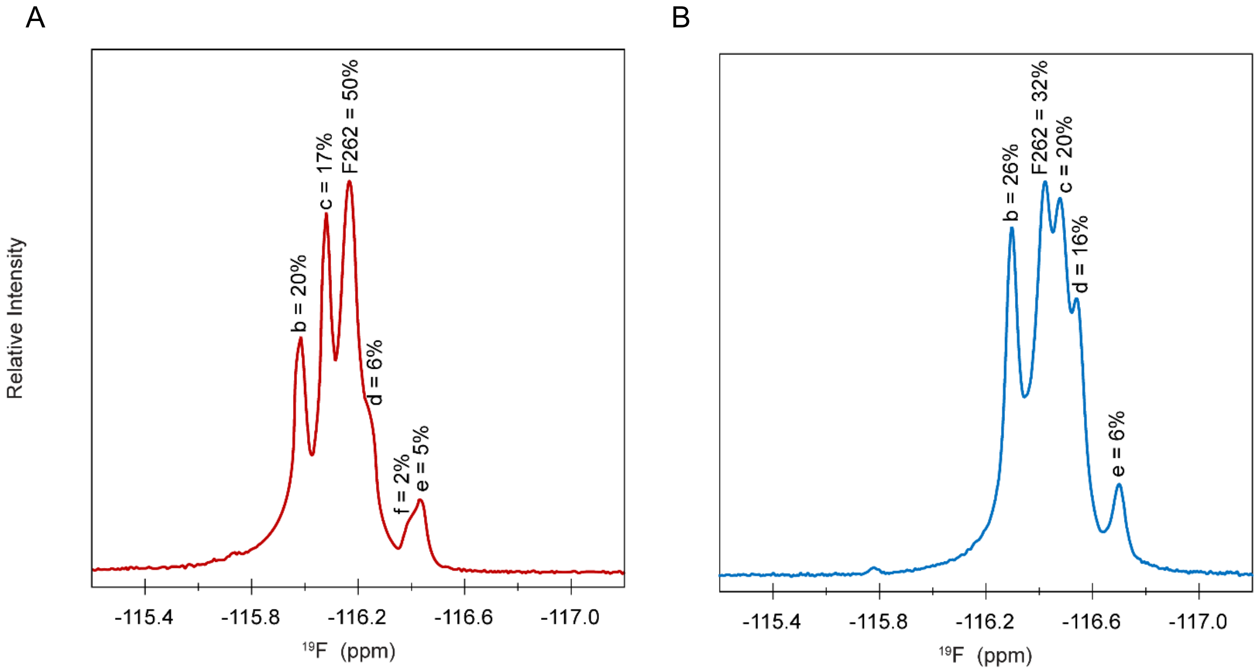

Figure S14. Site specific ^{19}F labeling of F^{262} in the LASV FD revealed additional conformers adopted by the side chain that were not observed in the backbone assignment. [A,B] Each F^{262} conformer is occupied for different percentages of time in the [A] pre- and [B] post-fusion state. All measurements were carried out on ~1,000 µM of $F^{293}W$ in 300 µL of 25 mM Na_2HPO_4, 100 mM NaCl, pH 7.0 at 20 ℃ (pre-fusion) or pH 4.0 with acidic bicelles, q = 0.5 at 45 ℃ (post-fusion). The most populated conformer (a) is the residue label, whereas the second (b), third (c), fourth (d), fifth (e), and sixth (f) most populated conformers are indicated as such.

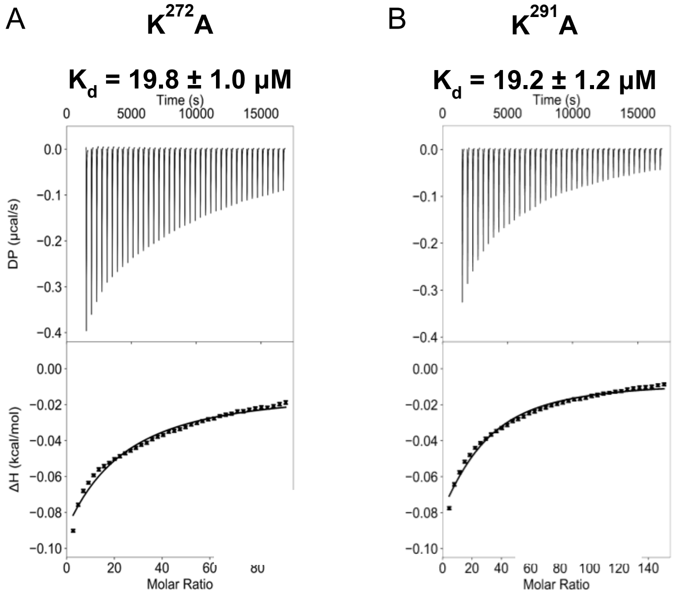

Figure S15. Mutation of the charged lysine residue located within the FP (K^{272}) or FL (K^{291}) to a chemically inert alanine residue had no impact on the binding affinity of the LASV FD. [A] K^{272}A and [B] K^{291}A mutant FD. Both ITC experiments were conducted in 10 mM NaOAc, 100 mM NaCl, pH 4.0 with 65:35 POPC:POPG vesicles titrated into the protein. Dissociation constants (K_d) are displayed above the respective isotherm.

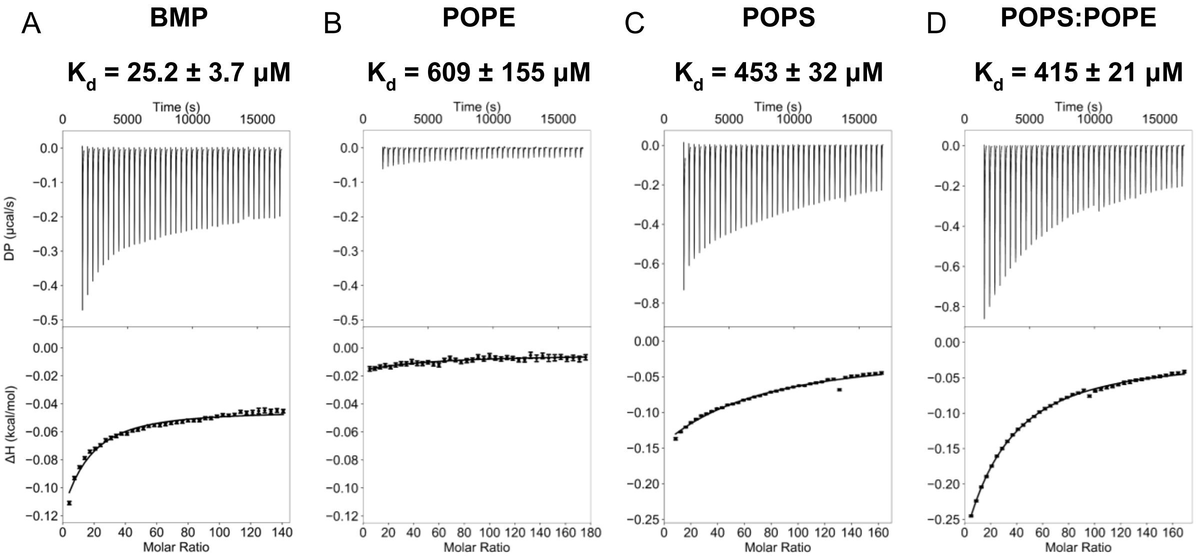

Figure S16. The LASV FD has the highest binding affinity for vesicles comprised of BMP. [A] 65:35 POPC:BMP; [B] 65:35 POPC:POPE; [C] 65:35 POPC:POPS; and [D] 65:17.5:17.5 POPC:POPS:POPE were titrated into the protein. All ITC experiments were conducted in 10 mM NaOAc, 100 mM NaCl, pH 4.0 with vesicles titrated into the protein. Dissociation constants (K_d) are displayed above the respective isotherm.

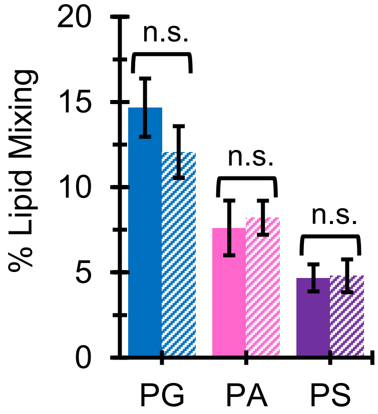

Figure S17. Tail saturation has no impact on LASV FD-initiated fusion. Lipids with a single unsaturated tail (POXX, solid color) and double unsaturated tail (DOXX, dashed color) did not have significant impact on the ability of the LASV FD to initiate fusion if the same head group moiety was present, i.e., PG (blue), PA (pink), and PS (purple) ($n \geq 9$). Student's t–test assuming unequal variances used to calculate the *P*-value; n.s. = not significant.

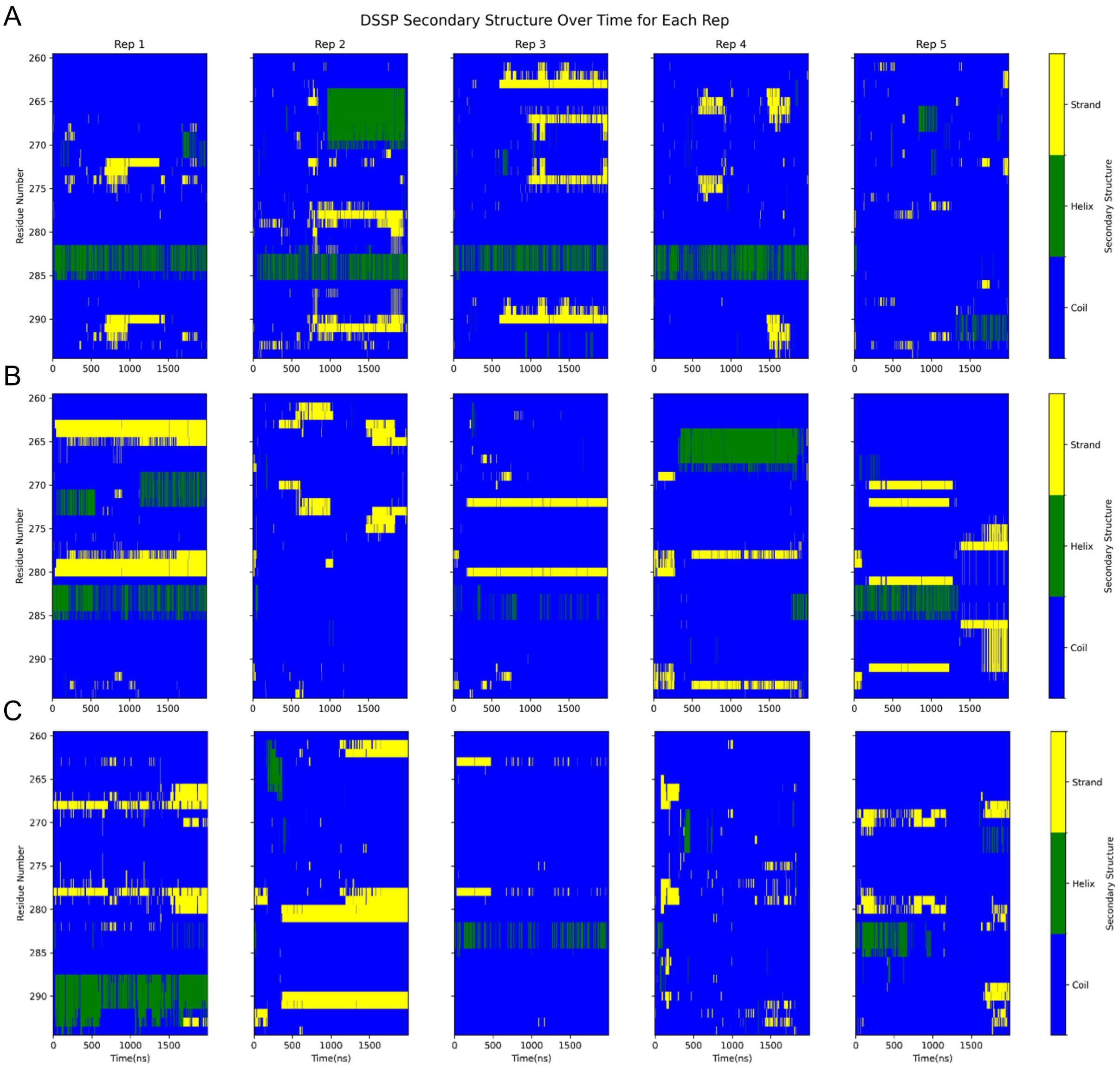

Figure S18. In MD simulations, the LASV FD can associate with membranes containing POPG and BMP, but not POPS. [A-C] Helical graph over the time course of the simulation for the different replicates indicates that the LASV FD has helical content in [A] POPC:POPG and [B] POPC:BMP, but relatively transient in [C] POPC:POPS.

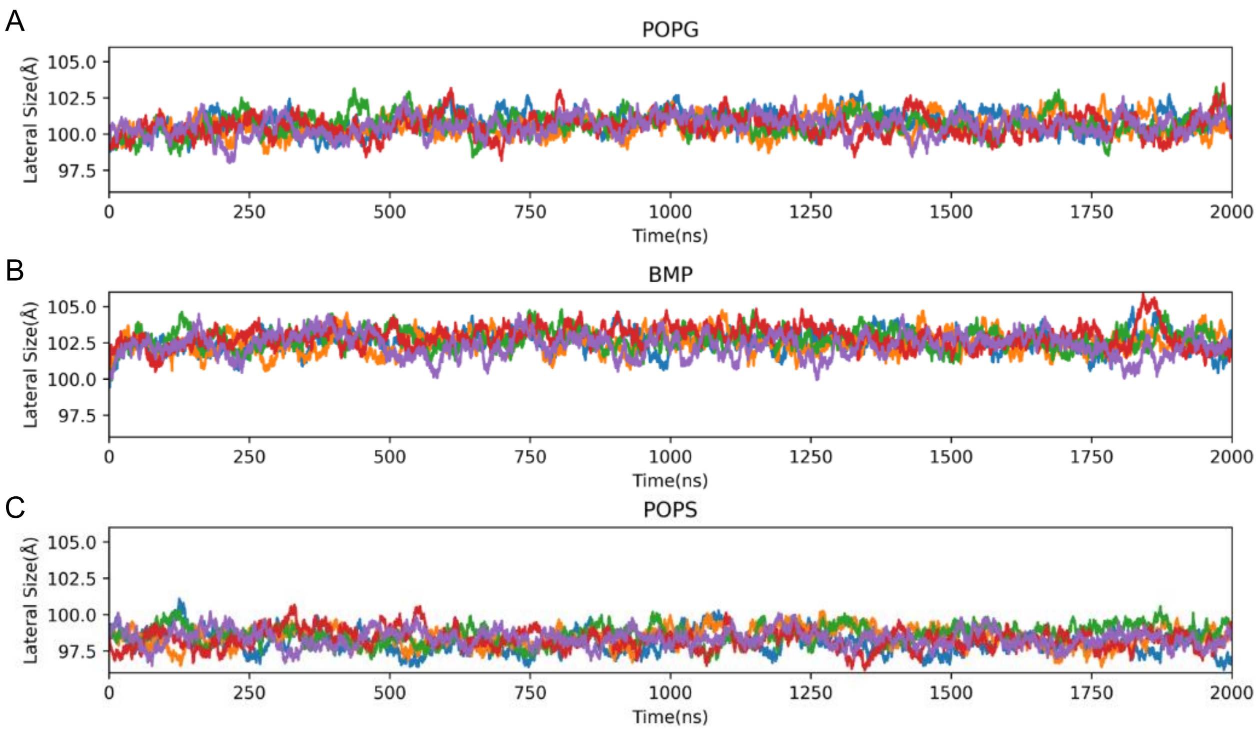

Figure S19. MD simulation showing the effect of the LASV FD on packing of different membrane environment. [A-C] shows how the lateral size of membrane changes over the time course in the presence of [A] POPC:POPG [B] POPC:BMP and [C] POPC:POPS.

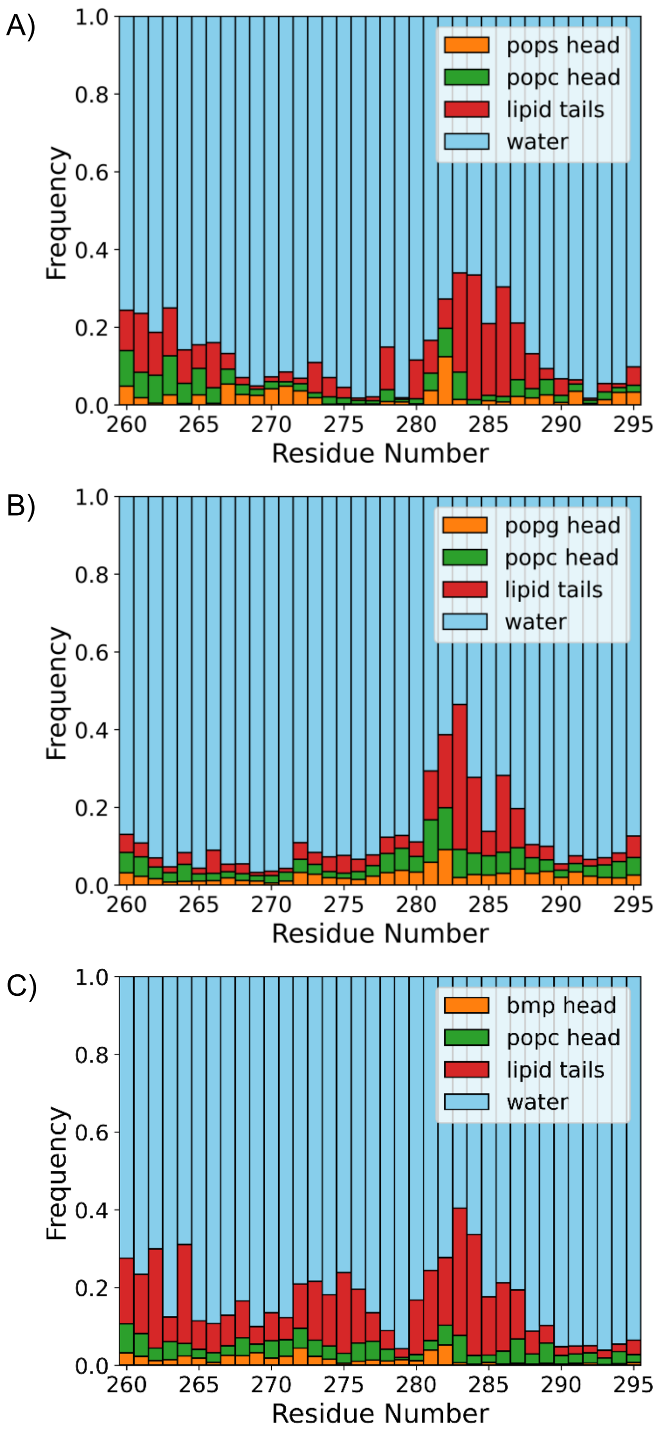

Figure S20. Contact analysis of LASV FD in different anionic lipid membranes. The frequency of contact is shown with water (blue), POPC head (green), head group of specific lipids (orange) [A] POPC:POPS, [B]POPC:POP, and [C] POPC:BMP, and lipid tails (red).

Table S1. Lipid composition of each membrane system utilized in molecular dynamics (MD) simulations.

Membrane System	POPC (16:0, 18:1 (9Z))	POPG (16:0, 18:1 (9Z))	BMP (18:1, 18:1)	POPS (16:0, 18:1 (9Z))	Total Lipid Count per Leaflet
POPC:POPG	104	56	0	0	160
POPC:BMP	104	0	56	0	160
POPC:POPS	104	0	0	56	160

Table S2. The LASV FD is largely a random coil in the pre-fusion state. A three-state backbone code was assigned to each residue via TALOS+ to relate the NMR chemical shifts and backbone torsion angles: A – alpha, -160 < Φ < 0 and -70 < Ψ < 60; P – positive, 0 < Φ < 160 and -60 < Ψ < 95; B – all others.

Residue	A	P	B	Φ (deg)	Ψ (deg)	Classification
G260	X	X	X	X	X	X
T261	0.3629	0.0445	0.5926	9999	9999	B
F262	0.02	0.0038	0.9762	-103.36	135.905	B
T263	0.0072	0.0012	0.9916	-124.138	135.929	B
W264	0.0091	0.002	0.9889	-140.552	160.013	B
T265	0.0026	0	0.9974	-90.04	142.794	B
L266	0.1901	0.0053	0.8046	-81.972	133.706	B
S267	0.1238	0.0067	0.8695	-87.453	131.924	B
D268	0.9132	0	0.0868	-62.05	-37.185	A
S269	0.9256	0.0081	0.0663	-66.001	-35.269	A
E270	0.7835	0.0149	0.2016	-99.847	2.97	A
G271	0.0891	0.7638	0.1471	87.45	5.133	P
K272	0.0459	0	0.9541	-77.97	145.786	B
D273	0.1483	0.0232	0.8285	-90.186	133.528	B
T274	0	0	1	-87.326	126.207	B
P275	0.1642	0.0092	0.8265	-56.491	139.354	B
G276	0.0379	0.9368	0.0253	71.27	13.882	P
G277	0.0665	0.7898	0.1437	87.597	2.132	P
Y278	0.0146	0	0.9854	-72.939	140.26	B
C279	0.5061	0.0253	0.4686	-69.064	-29.003	A
L280	0.9162	0.0138	0.07	-94.849	-20.5	A
T281	0.0559	0.0041	0.94	-140.896	125.101	B
R282	0.8469	0	0.1531	-76.105	-28.183	A
W283	0.7979	0.0154	0.1867	-72.422	-30.108	A
M284	0.8202	0.0143	0.1655	-71.043	-23.787	A
L285	0.1161	0.0255	0.8584	-75.178	130.323	B
I286	0.0266	0	0.9734	-97.401	137.845	B
E287	0.1494	0.0082	0.8423	-89.871	136.457	B
A288	0.9317	0.0077	0.0606	-70.208	-26.748	A
E289	0.2645	0.0476	0.688	-103.589	147.751	B
L290	0.0107	0.0056	0.9837	-81.757	136.902	B
K291	0.0281	0.0015	0.9704	-100.366	146.75	B
C292	0.0171	0	0.9829	-87.151	116.576	B
F293	0.3211	0.0255	0.6535	-104.298	141.561	B
G294	0	0.0031	0.9969	-127.736	162.394	B
N295	0.372	0.0498	0.5782	9999	9999	B

Table S3. Predicted dihedral angles suggest that the LASV FD has a helical structure within its FL in its post-fusion state. A three-state backbone code was assigned to each residue via TALOS+ to relate the NMR chemical shifts and backbone torsion angles: A – alpha, $-160 < \Phi < 0$ and $-70 < \Psi < 60$; P – positive, $0 < \Phi < 160$ and $-60 < \Psi < 95$; B – all others. The residues likely to form a helix (black box) are located within the FL from R^{282} to L^{290}.

Residue	A	P	B	Φ (deg)	Ψ (deg)	Classification
G260	0.5068	0.0437	0.4495	9999	9999	N
T261	0.0235	0	0.9765	-96.604	138.83	B
F262	0.3067	0.013	0.6804	-101.298	129.364	B
T263	0.1028	0.0218	0.8755	-109.042	138.603	B
W264	0.0032	0.004	0.9928	-138.058	163.49	B
T265	0.0019	0	0.9981	-88.179	152.043	B
L266	0.5564	0	0.4436	-82.183	137.134	A
S267	0.4401	0.0407	0.5192	-66.043	-34.703	B
D268	0.9278	0.0036	0.0686	-68.762	-31.162	A
S269	0.9466	0.0073	0.0461	-65.386	-34.876	A
E270	0.9587	0	0.0413	-97.485	0.763	A
G271	0.0986	0.8361	0.0653	78.626	16.904	P
K272	0.1452	0.012	0.8428	-75.683	144.486	B
D273	0.169	0.0318	0.7993	-86.531	141.921	B
T274	0	0	1	-94.543	126.679	B
P275	0.259	0.0147	0.7264	-56.491	139.354	B
G276	0.0389	0.943	0.0181	68.861	18.746	P
G277	0.0694	0.8347	0.0959	86.458	1.243	P
Y278	0.0216	0	0.9784	-83.047	138.209	B
C279	0.6291	0.0277	0.3432	-80.975	115.565	A
L280	0.0176	0.0008	0.9816	-105.654	134.554	B
T281	0.1842	0.0049	0.8109	-86.045	142.567	B
R282	0.9033	0	0.0967	-69.85	-31.059	A
W283	0.9265	0.0195	0.0539	-72.727	-33.439	A
M284	0.9522	0.0014	0.0464	-66.117	-36.429	A
L285	0.987	0.0043	0.0088	-78.996	-30.413	A
I286	0.8864	0.0063	0.1073	-73.005	-27.414	A
E287	0.9854	0.0061	0.0085	-67.92	-37.857	A
A288	0.9877	0.0033	0.009	-64.826	-33.075	A
E289	0.9816	0.0047	0.0136	-79.945	-20.587	A
L290	0.6746	0.016	0.3094	-84.02	-26.751	A
K291	0.2883	0.0155	0.6962	-101.144	146.926	B
C292	0.2778	0.0155	0.7066	-72.498	-28.285	B
F293	0.9382	0.0141	0.0476	-100.604	-7.119	A
G294	0	0.0459	0.9541	-121.627	146.372	B
N295	0.3975	0.0624	0.5401	9999	9999	N

Table S4. Comparison of the average relative intensities of the LASV FD in different paramagnetic probes. In the pre-fusion state, both components of the FD have similar signals and are solvent-exposed. However, in the post-fusion state, the FP remains solvent-exposed, whereas the FL was inserted into the lipid head group of the membrane.

	**PRE-FUSION**	**POST-FUSION**		
	**Gd-DTPA**	**Gd-DTPA**	**5-DSA**	**16-DSA**
FD	0.420 ± 0.021	0.222 ± 0.026	0.439 ± 0.037	0.620 ± 0.027
FP	0.458 ± 0.027	0.126 ± 0.022	0.530 ± 0.068	0.623 ± 0.046
FL	0.431 ± 0.025	0.317 ± 0.038	0.353 ± 0.034	0.620 ± 0.039

Table S5. Comparison of the overall relaxation times of the LASV FD in the different fusion states. In the pre-fusion state, the FP was slightly more flexible than the FL with values supportive of a random coil conformation. The FP continued to be more flexible than the FL in the post-fusion state, but to a much greater extent, with the FL becoming restricted.

	**PRE-FUSION**			**POST-FUSION**		
	**$^{1}H - ^{15}N$ NOEs (s)**	**R_1 (s^{-1})**	**R_2 (s^{-1})**	**$^{1}H - ^{15}N$ NOEs (s)**	**R_1 (s^{-1})**	**R_2 (s^{-1})**
FD	0.448 ± 0.027	1.735 ± 0.091	4.941 ± 0.356	0.419 ± 0.038	1.305 ± 0.040	6.109 ± 0.683
FP	0.369 ± 0.018	1.750 ± 0.159	4.137 ± 0.550	0.253 ± 0.034	1.280 ± 0.036	4.426 ± 0.390
FL	0.497 ± 0.046	1.651 ± 0.062	5.708 ± 0.501	0.564 ± 0.048	1.339 ± 0.078	7.947 ± 1.224

Table S6. The percentage that a given conformer is populated is residue-dependent in both the pre- and post-fusion states. Notably, in the pre-fusion state, the signal for the b conformer of W[264] and a conformer of D[268] (bolded) overlap with each other and are not an accurate representation of the conformer populations. In turn, peak intensities in the backbone strips were used to justify the assignment of each population. Residues that do not occupy a given conformer are blacked out. The first (a), second (b), third (c), and fourth (d) most populated conformers of a given residue are shown.

		PRE-FUSION				POST-FUSION			
		a	b	c	d	a	b	c	d
FP	F262	53%	37%	10%		59%	26%	9%	6%
FP	T263	76%	13%	11%		45%	28%	27%	
FP	**W264**	**34%**	**56%**	**10%**		70%	13%	9%	8%
FP	T265	71%	29%			53%	29%	18%	
FP	L266	38%	25%	22%	15%	40%	24%	20%	16%
FP	S267	79%	21%			68%	32%		
FP	**D268**	**78%**	**22%**			100%			
FP	K272	91%	9%			88%	12%		
	T274	95%	5%			91%	9%		
	Y278	43%	27%	23%	7%	100%			
FL	C279	71%	29%			61%	39%		
FL	L285	77%	23%			100%			
FL	I286	47%	35%	18%		100%			
FL	E287	89%	11%			100%			
FL	A288	79%	21%			100%			
FL	E289	46%	32%	22%		83%	17%		
FL	L290	67%	67%	12%		100%			
FL	C292	100%				57%	43%		
	F293	100%				78%	22%		
	**Average**	**67 ± 19%**	**24 ± 12%**	**16 ± 6%**	**11 ± 6%**	**67 ± 17%**	**25 ± 11%**	**16 ± 8%**	**10 ± 6%**

Table S7. In the pre-fusion state, the different conformers of the LASV FD have similar predicted dihedral angles. A three-state backbone code was assigned to each residue via TALOS+ to relate the NMR chemical shifts and backbone torsion angles: A – alpha, $-160 < \Phi < 0$ and $-70 < \Psi < 60$; P – positive, $0 < \Phi < 160$ and $-60 < \Psi < 95$; B – all others. The most populated conformer (a) is the residue label, whereas the second (b), third (c), and fourth (d) most populated conformers are indicated as such.

Residue	A	P	B	Φ (deg)	Ψ (deg)	Classification
F262	0.0200	0.0038	0.9762	-103.360	135.905	B
F262b	0.4000	0.0072	0.9528	-99.874	146.588	B
F262c	0.0147	0.0016	0.9837	-107.236	141.816	B
T263	0.0072	0.0012	0.9916	-124.138	135.929	B
T263b	0.0057	0.0015	0.9928	-127.687	138.507	B
T263c	0.0233	0.0029	0.9738	-108.678	131.182	B
W264	0.0091	0.0020	0.9889	-140.552	160.013	B
W264b	0.0096	0.0021	0.9883	-140.353	158.993	B
W264c	0.0065	0.0023	0.9912	-132.191	159.132	B
T265	0.0026	0	0.9974	-90.040	142.794	B
T265b	0.0029	0	0.9971	-94.818	150.419	B
L266	0.1901	0.0053	0.8046	-81.972	133.706	B
L266b	0.2166	0.0018	0.7816	-81.972	133.706	B
L266c	0.1909	0.0036	0.8055	-81.972	133.706	B
L266d	0.1845	0.0027	0.8128	-81.972	133.706	B
S267	0.1238	0.0067	0.8695	-87.453	131.924	B
S267b	0.2735	0.0140	0.7124	-63.324	-37.756	B
D268	0.9132	0	0.0868	-62.050	-37.185	A
D268b	0.9444	0.0010	0.0546	-64.607	-35.129	A
K272	0.0459	0	0.9541	-77.970	145.786	B
K272b	0.0468	0	0.9532	-79.519	136.657	B
T274	0	0	1	-87.326	126.207	B
T274b	0	0	1	-115.991	127.654	B
Y278	0.0146	0	0.9854	-72.939	140.260	B
Y278b	0.0189	0	0.9811	-72.939	140.260	B
Y278c	0.0034	0	0.9966	-76.794	141.392	B
Y278d	0.0036	0	0.9966	-77.226	143.301	B
C279	0.5061	0.0253	0.4686	-69.064	-29.003	A
C279b	0.3114	0.0077	0.6808	-101.077	142.122	B
L285	0.1161	0.0255	0.8584	-75.178	130.323	B
L285b	0.4407	0.0586	0.5007	-69.516	126.270	B
I286	0.0266	0	0.9734	-97.401	137.845	B
I286b	0.0120	0.0056	0.9824	-77.489	131.803	B
I286c	0.0445	0.0009	0.9546	-96.844	150.896	B
TABLE S7 CONTINUED ON NEXT PAGE						

TABLE S7 CONTINUED						
**Residue**	**A**	**P**	**B**	**Φ (deg)**	**Ψ (deg)**	**Classification**
E287	0.1494	0.0082	0.8423	-89.871	136.457	B
E287b	0.0429	0	0.9571	-90.715	144.977	B
A288	0.9317	0.0077	0.0606	-70.208	-26.748	A
A288b	0.9257	0.0110	0.0633	-75.261	-21.553	A
E289	0.2645	0.0476	0.688	-103.589	147.751	B
E289b	0.1203	0.0278	0.8519	-93.807	146.772	B
E289c	0.0893	0.0236	0.8871	-87.517	134.364	B
L290	0.0107	0.0056	0.9837	-81.757	136.902	B
L290b	0.0155	0.0038	0.9808	-80.729	134.134	B
L290c	0.0121	0.0045	0.9834	-85.355	130.667	B

Table S8. Predicted dihedral angles of the different conformers are relatively similar for the LASV FD in the post-fusion state. A three-state backbone code was assigned to each residue via TALOS+ to relate the NMR chemical shifts and backbone torsion angles: A – alpha, -160 < Φ < 0 and -70 < Ψ < 60; P – positive, 0 < Φ < 160 and -60 < Ψ < 95; B – all others. The most populated conformer (a) is the residue label, whereas the second (b), third (c), and fourth (d) most populated conformers are indicated as such.

Residue	A	P	B	Φ (deg)	Ψ (deg)	Classification
F262	0.3067	0.0130	0.6804	-101.298	129.364	B
F262b	0.2659	0.0236	0.7105	-104.883	132.792	B
F262c	0.0752	0.0056	0.9191	-102.444	131.969	B
F262d	0.0666	0.0052	0.9282	-102.444	131.969	B
T263	0.1028	0.0218	0.8755	-109.042	138.603	B
T263b	0.0019	0.0049	0.9932	-123.795	140.850	B
T263c	0.0291	0.0049	0.9660	-111.131	141.192	B
W264	0.0091	0.0020	0.9889	-140.552	160.013	B
W264b	0.0095	0.0020	0.9885	-139.437	159.124	B
W264c	0.0067	0.0027	0.9906	-140.259	165.618	B
W264d	0.0069	0.0026	0.9905	-140.259	165.618	B
T265	0.0019	0	0.9981	-88.179	152.043	B
T265b	0.0016	0	0.9984	-92.295	154.232	B
T265c	0.0023	0	0.9977	-94.363	154.151	B
L266	0.5564	0	0.4436	-82.183	137.134	A
L266b	0.3562	0	0.6438	-82.183	137.134	B
L266c	0.8594	0	0.1406	-68.489	-24.282	A
L266d	0.8645	0	0.1355	-68.489	-24.282	A
S267	0.4401	0.0407	0.5192	-66.043	-34.703	B
S267b	0.6084	0.0075	0.3841	-64.255	-33.783	A
K272	0.1452	0.0120	0.8428	-75.683	144.486	B
K272b	0.1128	0.0049	0.8823	-74.603	134.747	B
T274	0	0	1	-94.543	126.679	B
T274b	0	0	1	-119.516	126.546	B
C279	0.6291	0.0277	0.3432	-80.975	115.565	A
C279b	0.5678	0.0284	0.4038	-75.107	122.862	A
E289	0.9816	0.0047	0.0136	-79.945	-20.587	A
E289b	0.9869	0.0046	0.0085	-79.557	-19.575	A
C292	0.2778	0.0155	0.7066	-72.498	-28.285	B
C292b	0.2577	0.0108	0.7315	-72.498	-28.285	B
F293	0.9382	0.0141	0.0476	-100.604	-7.119	A
F293b	0.9430	0.0135	0.0436	-104.646	-3.126	A
