# Supplementary material for: The Lassa virus fusion domain has structural plasticity and exploits bis(monoacylglycero)phosphate for fusion
Source: Biophys J. 2026 Feb 13;125(5):1384–402. doi: 10.1016/j.bpj.2026.01.049 (PMC13351859; doi:10.1016/j.bpj.2026.01.049)
Supplement: Document S2. Article plus supporting material [file mmc5.pdf]

Biophysical Journal
Article

Biophysical Society

The Lassa virus fusion domain has structural plasticity and exploits bis(monoacylglycero)phosphate for fusion

Hallie N. Pennington,[1] Kiruthika Prahadeesh,[1] Quinn M. Mulvihill,[1] Sungjai Shin,[1] Wonpil Im,[2] and Jinwoo Lee[1,*]
[1]Department of Chemistry and Biochemistry, College of Computer, Mathematics, and Natural Science, University of Maryland, College Park, Maryland and [2]Department of Biological Sciences, College of Arts and Sciences, Lehigh University, Bethlehem, Pennsylvania

ABSTRACT Infection with Lassa virus (LASV), an arenavirus endemic to West Africa, results in a viral hemorrhagic fever with high mortality rates and public health implications. The glycoprotein complex is central to LASV's infectivity as it mediates viral entry via membrane fusion. The fusion domain (FD) (G^{260}-N^{295}) facilitates the initiation of membrane fusion and, thus, the merging of the viral and host cell membranes in a pH-dependent fashion at the lysosomal membrane. The FD consists of two distinct regions: an N-terminal fusion peptide (FP) (G^{260}-T^{274}) and an internal fusion loop (FL) (C^{279}-N^{295}) that are connected by a short linker region (P^{275}-Y^{278}). Nonetheless, the precise structural and functional characteristics of the LASV FD remain unknown. Here, we demonstrate that the LASV FD associates with the host cell membrane via its FL, specifically residues R^{282}-L^{290}, whereas the FP is more solvent exposed, especially for residues D^{268}-T^{274}. We found that a multitude of conformational states are adopted by the entire LASV FD before membrane association, while only the FP, and not the FL, continues to sample numerous states after membrane association. Moreover, we provide evidence that the LASV FD prefers to interact with anionic lipids, namely bis(monoacylglycero)phosphate (BMP). In conclusion, our findings indicate that the LASV FD preferentially initiates fusion in the presence of BMP, at which point the FL adopts a helical conformation to associate with the membrane, whereas the FP remains exposed to the environment.

SIGNIFICANCE Membrane fusion is a fundamental biological process utilized by enveloped viruses to enter host cells, yet the physical mechanisms governing how lipid-protein interactions regulate this step remain poorly understood. This study shows that the Lassa virus fusion domain is structurally plastic and selectively engages bis(monoacylglycero)phosphate, a lipid enriched in late endosomal membranes. Using biophysical and structural approaches, we demonstrate that this specific lipid environment promotes a helical conformational change that enhances membrane fusion efficiency. These findings reveal how viral fusion proteins respond to lipid composition to trigger fusion at the appropriate cellular location, advancing our understanding of lipid-dependent mechanisms underlying viral membrane fusion.

INTRODUCTION

Lassa virus (LASV), a member of the *Arenaviridae* family, is a zoonotic pathogen responsible for Lassa fever (LF), a severe hemorrhagic fever that poses a significant public health threat, chiefly in West Africa where it is endemic (1–4). Annually, an estimated 100,000 to 300,000 individuals are infected with LASV, but poor healthcare in endemic regions has caused an underrepresentation of cases. The case fatality rate of LASV historically averages around 20%, especially in severe cases and pregnant individuals, leading to thousands of annual deaths (5–8). LASV is most commonly spread by contact with excrement from infected *Mastomys natalensis* rodents, but an increased incidence of direct human-to-human transmission has been observed in recent years (9–11). Despite its clinical importance, there are presently no FDA-approved therapeutic options for the explicit treatment of LF (12,13). It thus comes as no surprise that the World Health Organization has categorized LASV as one of the top 5 infectious agents requiring prioritized research and development due to its pandemic potential, should it complete its zoonotic jump (14,15).

Submitted August 15, 2025, and accepted for publication January 28, 2026.
*Correspondence: jinwoo@umd.edu
Editor: Daniel Huster.
https://doi.org/10.1016/j.bpj.2026.01.049
© 2026 The Author(s). Published by Elsevier Inc. on behalf of Biophysical Society.
This is an open access article under the CC BY license (http://creativecommons.org/licenses/by/4.0/).

```
                    Fusion Peptide ........ Fusion Loop
LASV**    260  GTFTWTLSDSEGKDTPGGYCLTRWMLIEAELKCFGN  295
LCMV**    266  GTFTWTLSDSSGVENPGGYCLTKWMILAAELKCFGN  301
LUJOV**   219  KLFQWSLSDETGSPLPGGHCLERWLIFASDIKCFDN  267
DNDV      266  GTFTWTLSDSSGTENPGGYCLTKWMLIAAELKCFGN  301
IPPYV     264  STFTWSLSDSSGTENPGGYCLTRWMLFAADLKCFGN  299
MOBV      260  GTFTWTLSDSEGNDLPGGYCLQRWMLIEAEMKCFGN  295
MOPV      258  GLFTWTLSDSEGNDMPGGYCLTRSMLIGLDLKCFGN  293
                 * *:***. *   ***:** : :::  ::***.*
```

FIGURE 1 Multiple sequence alignment of the FD in the Old-World clade of the arenavirus family. The structurally distinct regions that make up the FD, the fusion peptide (FP) (*yellow*) and internal fusion loop (FL) (*orange*), are conjoined by a short linker region. Positively charged residues within the LASV FD are highlighted (*bold*, *green*) with those studied in this article denoted (*box*). Viruses with pathogenic potential in humans are indicated by **. Residues with full conservation are indicated by an asterisk (*), whereas those with strong and weak similarities are denoted by a semicolon (;) and period (.), respectively. LCMV, lymphocytic choriomeningitis virus; LUJOV, Lujo virus; DNDV, Dandenong virus; IPPYV, Ippy virus; MOBV, Mobala virus; MOPV, Mopeia virus.

LASV relies on membrane fusion to enter the host cell, a process facilitated by the glycoprotein complex (GPC) (16–18) (Fig. S1). The GPC is the sole protein on the virion surface and is comprised of a receptor binding subunit (glycoprotein 1 [GP1]) and a fusion subunit (glycoprotein 2 [GP2]) that is associated with a stable signal peptide. After an initial interaction between GP1 and its primary receptor, α-dystroglycan, which is located on the host cell plasma membrane, the virion is endocytosed and delivered late within the endocytic pathway, where fusion ultimately occurs via the six-helix bundle mechanism (19–23). More specifically, the low pH environment triggers GP1 to undergo conformational changes, allowing it to engage with its secondary receptor, lysosomal-associated membrane protein 1, and dissociate from the GPC (24–28). A hydrophobic sequence at the N-terminus of GP2, known as the fusion domain (FD), is subsequently exposed and anchors itself into the host cell membrane. This results in the initiation of membrane fusion, leading to the formation of a fusion pore and delivery of LASV's genetic material into the host cell. Thus, it is evident that the LASV FD has an essential role in viral entry and its structure before (pre-) and after (post-) fusion should be further explored.

LASV is a class one fusion protein, similar to human immunodeficiency virus (HIV), influenza, Ebola virus (EBOV), and severe acute respiratory coronavirus 2 (SARS-CoV-2). Typically, the FD of class one fusion proteins is an N-terminal fusion peptide (FP), as seen in HIV and influenza (29–37). In some cases, however, an internal fusion loop (FL) has been observed, such as that found in EBOV (38,39). The FD of LASV and other arenaviruses unusually contains both an FP (G^{260}-T^{274}) and FL (C^{279}-N^{295}), which are connected by a short linker region (P^{275}-Y^{278}) (Fig. 1) (40–43). Notably, the internal disulfide bond within the FL is located between residues C^{279} and C^{292}. This feature is shared only with coronaviruses, which includes SARS-CoV-2, the causative agent of coronavirus disease 2019 (COVID-19). While the SARS-CoV-2 FP and FL can function independently, they are most efficient in synergy (44–51). It has been postulated that the SARS-CoV-2 FD functions as an evolutionary hybrid, potentially contributing to the remarkable infectivity of COVID-19. However, many aspects of the LASV FD's molecular mechanisms, particularly those governing its ability to mediate fusion and the roles of both the FP and FL, remain poorly understood. In the literature, it has been demonstrated that the LASV FD undergoes a significant conformational change from the pre- to postfusion state, as it transitions from a nonfusogenic coil to a fusogenic helix that can associate with the target host cell membrane, primarily the lysosomal membrane (42). For clarity, the nonfusogenic coil conformation will be referred to as the prefusion state, and fusogenic helix conformation as the postfusion state in this manuscript. Nonetheless, the exact location of the helix, residues that perturb the membrane, and influence of lipids found within the lysosomal membrane are unknown.

In this study, we aimed to elucidate the structure of the LASV FD in its pre- and postfusion states and the functional influence of key lipids found within the lysosomal membrane. Through a combination of NMR techniques, we illustrate a conformational change undergone by the LASV FD after its association with the membrane. In the postfusion state, the FL, namely residues R^{282}-L^{290}, adopts a helix that embeds itself just below the lipid headgroup of the membrane. In contrast, the FP, mainly residues D^{268}-T^{274}, remains exposed to the aqueous environment. Multiple conformational states are adopted by both the FP and FL in the prefusion state, but this only applies to the FP after fusion occurs. Additionally, the LASV FD is partial to associate, and thus, initiate fusion, in membranes comprised of anionic lipids, especially bis(monoacylglycero)phosphate (BMP). We provide evidence that this interaction is not facilitated by positively charged, lysine residues (K^{272} and K^{291}) as mutagenesis of these residues did not have significant impacts on secondary structure, membrane affinity, or fusion. Together, this study illuminates how the LASV FD associates with the membrane and shows its preference to initiate fusion with anionic lipids, chiefly BMP, which is not mediated by an ionic interaction with lysine residues.

MATERIALS AND METHODS

Lipids

1,2-Dimyristoyl-*sn*-glycero-3-[phospho-rac-(1-glycerol)] (DMPG) was purchased from Anatrace (Maumee, OH). 16:0–18:1 1-palmitoyl-2-oleoyl-glycero-3-phosphocholine (POPC), 16:0–18:1 1-palmitoyl-2-oleoyl-*sn*-glycero-3-phospho-[1′-rac-glycerol] (POPG) (Fig. S2 *A*), 1,2-dioleoyl-*sn*-glycero-3-[phospho-rac-(3-lysyl(1-glycerol))] (DOPG) (Fig. S2 *B*), 1-palmitoyl-2-oleoyl-*sn*-glycero-3-phosphate (POPA) (Fig. S2 *C*), 1,2-dioleoyl-*sn*-glycero-3-phosphate (DOPA) (Fig. S2 *D*), 1-palmitoyl-2-oleoyl-*sn*-glycero-3-phospho-L-serine (POPS) (Fig. S2 *E*), 1,2-dioleoyl-*sn*-glycero-3-phospho-L-serine (DOPS) (Fig. S2 *F*), 1-palmitoyl-2-oleoyl-*sn*-glycero-3-phosphoethanolamine (POPE) (Fig. S2 *G*), BMP (S,R isomer) (BMP) (Fig. S2 *H*), 18:1 1,2-dioleoyl-*sn*-glycero-3-phosphoethanolamine-*N*-[lissamine rhodamine B sulfonyl] (Rh-PE), 18:1 1,2-dioleoyl-*sn*-glycero-3-phosphoethanolamine-*N*-[7-nitro-2-1,3-benzoxadiazol-4-yl] (NBD-PE), 1,2-dimyristoyl-*sn*-glycero-3-phosphocholine (DMPC), and 1,2-dihexanoyl-*sn*-glycero-3-phosphocholine (DHPC) were all purchased from Avanti Polar Lipids (Alabaster, AL).

Expression and isotopic labeling

The expression protocol and single, ^{15}N-labeling of the LASV FD has been previously described (42,52). The lysine mutants (i.e., K^{272}A and K^{291}A) followed the designed expression protocol. However, the F^{293}W-containing vector was transformed into *Escherichia coli* strain DL39(DE3) auxotrophic cells instead of *E. coli* strain BL21(DE3)pLysS cells. For selective, ^{19}F-labeling, a single colony from the transformation was used to inoculate 5 mL of LB medium containing 50 μg/mL kanamycin (Thermo Fisher Scientific, Waltham, MA) and grown overnight (16–18 h) at 37°C and 225 rpm in a MaxQ 4000 incubated shaker (Thermo Fisher Scientific). The next morning, the starter culture was added to 1 L of MDAG minimal medium (25 mM Na_2HPO_4, 25 mM KH_2PO_4, 50 mM NH_4Cl, 5 mM Na_2SO_4, 2 mM $MgSO_4$, 0.2× trace metals, 0.5% glucose, 50 μg/mL kanamycin [pH 7.2]) supplemented with 200 μg/mL of each amino acid, except for Cys and Tyr, which were not added due to solubility issues (53). The trace metals came from a 1000× stock that contained 50 mM $FeCl_3$, 20 mM $CaCl_2$, 10 mM $MnCl_2$, 10 mM $ZnSO_4$, and 2 mM each of $CoCl_2$, $CuCl_2$, $NiCl_2$, Na_2MoO_4, Na_2SeO_3, and H_3BO_3 in 60 mM HCl. The cells were grown at 37°C and 225 rpm until an OD_{600} of 0.6 was achieved, as confirmed on an Ultrospec 1000 Spectrophotometer (Pharmacia Biotech, Cambridge, England), then harvested for 20 min at 4000 × *g* and 4°C in an Avanti J-15R centrifuge (Beckman Coulter, Brea, CA). Each pellet was subsequently resuspended in 250 mL of MDAG minimal medium that lacked any amino acids (25 mM Na_2HPO_4, 25 mM KH_2PO_4, 50 mM NH_4Cl, 5 mM Na_2SO_4, 2 mM $MgSO_4$, 0.2× trace metals, 0.5% glucose, 50 μg/mL kanamycin [pH 7.2]), then centrifuged for 20 min at 4000 × *g* and 4°C to remove any residual natural phenylalanine from the system. The rinsed cell pellets were resuspended in 1 L of ^{19}F-Phe MDAG minimal medium (100 mg 4-fluoro-D,L-phenylalanine [Fisher Scientific, Hampton, NH], 25 mM Na_2HPO_4, 25 mM KH_2PO_4, 50 mM NH_4Cl, 5 mM Na_2SO_4, 2 mM $MgSO_4$, 0.2× trace metals, 0.5% glucose, 50 μg/mL kanamycin [pH 7.2]) with 200 μg/mL of each amino acid except Cys, Try, and Phe. The resuspended cells were shaken for 30 min at 37°C and 225 rpm to give the cells time to adjust to the new medium. After 30 min, the temperature was decreased to 18°C and the cells were shaken at 225 rpm for an additional 30 min to allow the cells to acclimate to the new temperature. Protein expression was subsequently induced with 1 mM isopropyl β-D-1-thiogalactopyranoside (IPTG), and then the cells were incubated overnight (∼20 h) before being harvested at 4000 × *g* and 4°C for 45 min.

Selective, ^{15}N-Leu-labeling of the LASV FD was achieved in a similar manner as site specific, ^{19}F-labeling with some modifications. Initially, 4 × 120 mL propagated starter cultures of the LASV FD in *E. coli* BL21(de3)pLysS cells were added to 4 × 1 L of EMBL minimal medium (1 g/L NH_4Cl, 10 g/L glucose, 8 g/L Na_2HPO_4, 2 g/L KH_2PO_4, 0.5 g/L NaCl, 1 mM $MgSO_4$, 0.3 mM Na_2SO_4, 0.3 mM $CaCl_2$, 50 μg/mL kanamycin, 34 μg/mL chloramphenicol, trace amounts of biotin and thiamine) at 37°C and 225 rpm in a MaxQ 4000 incubated shaker until an OD_{600} between 0.6 and 0.8 was achieved, as measured on an Ultrospec 1000 Spectrophotometer. Upon reaching the appropriate OD_{600}, cells were harvested for 15 min at 4000 × *g* and 15°C in an Avanti J-15R such that each pellet contained 1 L worth of cells. Each pellet was subsequently resuspended in 250 mL of EMBL minimal medium that lacked a nitrogen source (10 g/L glucose, 8 g/L Na_2HPO_4, 2 g/L KH_2PO_4, 0.5 g/L NaCl, 1 mM $MgSO_4$, 0.3 mM Na_2SO_4, 0.3 mM $CaCl_2$, 50 μg/mL kanamycin, 34 μg/mL chloramphenicol, trace amounts of biotin and thiamine) then spun down for 15 min at 4000 × *g* and 15°C to remove any residual unlabeled media from the system. The rinsed cell pellets were resuspended in 1 L of ^{15}N-Leu EMBL minimal medium (1 g/L ^{15}N-Leu [Cambridge Isotope Laboratories, Tewksbury, MA], 10 g/L glucose, 8 g/L Na_2HPO_4, 2 g/L KH_2PO_4, 0.5 g/L NaCl, 1 mM $MgSO_4$, 0.3 mM Na_2SO_4, 0.3 mM $CaCl_2$, 50 μg/mL kanamycin, 34 μg/mL chloramphenicol, trace amounts of biotin and thiamine) with 10 g of each, unlabeled amino acid, except for Leu and Tyr. The addition of unlabeled amino acids in 10-fold excess over the desired amino acid to incorporate (Leu) reduces scrambling during protein expression, whereas Tyr was not included due to solubility issues (53,54). The resuspended cells were split into 2 × 500 mL and grown for 1 h at 37°C and 225 rpm to acclimate the cells to the new medium and temperature. Cells were then induced with 1 mM IPTG and incubated overnight (∼20 h) before being harvested at 4000 × *g* and 4°C for 45 min.

For double, ^{15}N/^{13}C isotopic labeling of the LASV FD, a comparable method to ^{15}N-Leu labeling was employed. However, cells were initially grown in 4 × 1 L of LB until the appropriate OD_{600}, rinsed with 250 mL of EMBL minimal medium lacking a carbon or nitrogen source (8 g/L Na_2HPO_4, 2 g/L KH_2PO_4, 0.5 g/L NaCl, 1 mM $MgSO_4$, 0.3 mM Na_2SO_4, 0.3 mM $CaCl_2$, 50 μg/mL kanamycin, 34 μg/mL chloramphenicol, trace amounts of biotin and thiamine), then resuspended in 1 L of ^{15}N/^{13}C EMBL minimal medium (1 g/L $^{15}NH_4Cl$ [Cambridge Isotope Laboratories], 2 g/L ^{13}C glucose [Cambridge Isotope Laboratories], 8 g/L Na_2HPO_4, 2 g/L KH_2PO_4, 0.5 g/L NaCl, 1 mM $MgSO_4$, 0.3 mM Na_2SO_4, 0.3 mM $CaCl_2$, 50 μg/mL kanamycin, 34 μg/mL chloramphenicol, trace amounts of biotin and thiamine). The cells were grown for1 h at 37°C and 225 rpm before being induced with 1 mM IPTG, incubated for an additional 4 h at 37°C and 225 rpm, and then harvested at 4000 × *g* and 4°C for 45 min. For ^{2}H/^{15}N/^{13}C isotopic labeling, the same *E. coli* BL21(DE3)pLysS strain was used without preadaptation to D_2O. Briefly, 1 L of D_2O was utilized to prepare the ^{15}N/^{13}C EMBL minimal medium as opposed to H_2O but expressed in the same manner as the ^{15}N/^{13}C sample; however, after induction with 1 mM IPTG, the cells were incubated for 8 h at 37°C and 225 rpm, then harvested with the same parameters. Cell pellets were stored at −80°C or immediately purified.

Purification

The purification protocol of the LASV FD has been described in detail previously (42,52). Briefly, a construct containing the entire FD [^{260}(GTFTWTLSDSEGKDTPGGYCLTRWMLIEAELKCFGN)^{295}] was designed with an N-terminal 9x-His tag followed by a trp operon leader sequence (TrpLE), and a thrombin cleavage site. The natural thrombin cleavage site was replaced with LVPR↓GT to yield the native sequence of the LASV FD after cleavage, wherein the downward arrow indicates the actual cleavage site of thrombin. Ni-NTA affinity chromatography and thrombin cleavage were employed to separate the protein from the tags. The separated protein was then subjected to size-exclusion chromatography with HMA buffer (10 mM HEPES/MES/NaOAc, 100 mM NaCl [pH 7.4]) or NMR buffer (25 mM Na_2HPO_4, 100 mM NaCl [pH 7.0]) to further isolate the protein and achieve the correct buffer system. All mutants (i.e., K^{272}A, K^{291}A, and F^{293}W) in this study were otherwise purified in the same manner as the LASV FD (WT).

NMR experiments

NMR spectra were acquired utilizing a Shigemi NMR tube with a sample volume of ~300 μL in NMR buffer pH 7.0 (prefusion) or pH 4.0 (postfusion) and 9:1 ratio of H_2O:D_2O. The protein concentration was ~500 μM for single, ^{15}N-labeled samples, ~650 μM for double, ^{15}N/^{13}C-labeled samples, ~1000 μM for the ^{15}N-Leu-labeled sample, and 1000 μM for the ^{19}F-labeled samples. For the postfusion state, 25% acidic bicelles ((75:25 DMPC:DMPG):DHPC) with a q value of 0.5 were combined with the sample, then the pH was dropped to 4.0 with 1 M HCl and verified with a calibrated pH probe. The q value was confirmed via phosphorous experiments on an Ascend 800 MHz magnet (Bruker, Billerica, MA) equipped with a CPQCI ^{1}H-^{31}P/^{13}C/^{15}N/D Z-GRD Cryoprobe (55). Experiments were carried out on either the Ascend 800 MHz magnet or an Ultrashield 600 MHz magnet (Bruker) with a CP2.1 TCI 600S3 H&F/C/N-D-05 Z XT Cryoprobe at a temperature of either 20°C (prefusion) or 45°C (postfusion) to improve peak sharpness (Fig. S3). A backbone assignment was completed for both states after HNCA, HN(CO)CA, HNCO, HN(CA)CO, and HN(CA)CB experiments using either 20 or 25% nonuniform sampling schedule. An HN(CA)CB experiment was run on the triple-labeled (^{2}H/^{15}N/^{13}C) sample for the prefusion state, whereas HNCA, HNCO, HN(CA)CO, and HN(CA)CB experiments were run for the postfusion state to improve signal/noise for the backbone assignment. All backbone experiments were processed via NMRPipe (56) and NMRFAM-SPARKY (57) via NMRBox (58). The chemical shift index (CSI) for each residue was deduced from the equation CSI = $C\alpha_{FD}$-$C\alpha_{BMRB}$ where $C\alpha_{FD}$ is the Cα measured for the LASV FD and $C\alpha_{BMRB}$ is the Cα of a given residue as published in the Biological Molecular Resonance Bank (BMRB) (59,60). CSI of N, H, and CO was performed in the same manner but with the values of N, H, or CO from BMRB subtracted from the N/H/CO of the FD. A chemical shift list was exported from NMRFAM-SPARKY in a format suitable for Torsion Angle Likelihood Obtained from Shift and Sequence Similarity (TALOS+) with the tf command (57,61). The file was adjusted to have the input table with the required data format, then the TALOS+ prediction was performed with the -iso option in the command line to correct the change in the chemical shift of ^{13}Cα and ^{13}Cβ due to ^{2}H isotopic labeling. Visual analysis of the resultant secondary structure was conducted on a Ramachandran map. Integrations for the multiple conformers of each residue were calculated in Bruker TopSpin, and then the percentage of each conformer was calculated from Population % $= \frac{|\int|}{\sum|\int|} \times 100\%$ where $|\int|$ is the absolute integration of a conformer and $\Sigma|\int|$ is the sum of the absolute integration for all conformers of a given residue. The Poky Suite was used to extract ^{1}H slices from the ^{1}H-^{15}N heteronuclear single quantum coherence (HSQC) of the pre- and postfusion state to visualize the populations of the different conformers for a given residue (62). All ^{19}F experiments were carried out on the Ultrashield 600 MHz magnet with a CP2.1 TCI 600S3 H&F/C/N-D-05 Z XT Cryoprobe, then processed using the software MestReNova (Mnova) within NMRBox.

Experiments probing molecular dynamics

Heteronuclear (^{1}H-^{15}N) NOEs, T_1, and T_2 experiments at pH 7.0 and pH 4.0 were conducted on the Ascend 800 MHz magnet equipped with a CPQCI ^{1}H-^{31}P/^{13}C/^{15}N/D Z-GRD Cryoprobe. Spin-lattice T_1 relaxation experiments were performed with a variable delay list of either 0.1, 0.3, 0.5, 0.8, 1.5, and 3 s or 0.1, 0.2, 0.5, 0.8, 1, 2, and 4 s for the pre- and postfusion states, respectively. Spin-spin T_2 relaxation experiments had a variable counter list of either 2, 4, 6, 8, 10, 20, 30, and 40 or 2, 4, 6, 8, 10, 15, 20, 30, and 40 for the pre- and postfusion states, correspondingly, yielding relaxation times of 0.03392, 0.06784, 0.10176, 0.13568, 0.1696, 0.2544, 0.5088, and 0.6784 s or 0.03392, 0.06784 0.10176, 0.13568, 0.1696, 0.2544, 0.3392, 0.5088 and 0.6784 s for the loops accordingly. Heteronuclear NOEs were processed via NMRPipe (56) and NMRFAM-SPARKY (57) via NMRBox (58). Data from the T_1 and T_2 experiments were processed in the Bruker TopSpin Dynamics Center, then converted to R_1 and R_2 by taking the inverse of T_1 and T_2, correspondingly. The error shown is propagated from the signal/noise or standard error of the mean (SEM).

Paramagnetic relaxation enhancement experiments

Membrane depth was probed utilizing gadolinium (III) diethylenetriaminepentaacetic acid (Gd-DTPA), 5-doxyl stearic acid (5-DSA), and 16-doxyl stearic acid (16-DSA). Gd-DTPA (100 mM) was solubilized in H_2O and then titrated into the NMR sample to achieve 0.5, 1, 2, 4, and 6 mM. For 5- and 16-DSA, known concentrations of DSA were added to glass tubes. A film was created by gently vortexing the tube under a nitrogen stream to remove the chloroform. The NMR sample was subsequently added to the glass tube to resuspend the DSA film and incorporate the paramagnetically tagged stearic acid into the system. The 5- and 16-DSA titrations were carried out to achieve concentrations of 0.5, 1, 2, 4, 6, and 8 mM. All data were collected through a ^{1}H-^{15}N HSQC at either 20°C (prefusion) or 45°C (postfusion). All spectra were processed using NMRPipe and NMRFAM-SPARKY via NMRBox to obtain peak intensities and signal/noise ratios for the titration of each relaxation agent. Relative intensities were calculated as $\frac{I_n}{I_o}$ where I_n is the peak intensity at titration point n and I_o is the maximum peak intensity. The error shown is from the signal/noise ratio or SEM.

Preparation of unilamellar vesicles

Large unilamellar vesicles (LUVs) were prepared by combining specified amounts of lipid stock solutions in glass test tubes. Chloroform was removed by applying a continuous nitrogen stream while gently vortexing the glass tube to create a lipid film before being left in a vacuum desiccator overnight to evaporate any residual chloroform. The following day, the lipid film was resuspended via vortexing in the appropriate volume of HMA buffer, subjected to 10 freeze-thaw cycles between liquid nitrogen and a 42°C water bath, then extruded 21 times through a double layer of 100 nm pore-size polycarbonate membranes (Avestin, Ottawa, ON, Canada). The lipid film for small unilamellar vesicles (SUVs) was created analogous to the LUVs but was resuspended in the appropriate volume of either circular dichroism (CD) buffer (1 mM HMA, 10 mM NaCl [pH 7.4]) or isothermal titration calorimetry (ITC) buffer (10 mM NaOAc, 100 mM NaCl [pH 4.0]), then sonicated on ice for 15 total min (1 s on, 1 s off, 10% power) using a Branson ultrasonicator furnished with a titanium microtip (Emerson, Danbury, CT). The transparent solution was subsequently centrifuged for 15 min at 20,000 × *g* in an Eppendorf 5425 microcentrifuge (Sigma-Aldrich, St. Louis, MO) to remove any particulates and transferred to a new microcentrifuge tube. All vesicles used in this study were comprised of 65 mol % POPC:35 mol % POPG, unless otherwise specified.

FRET-based fusion assay

Unlabeled LUVs comprised of 65:35 POPC:POPG and labeled LUVs of 63:35:1:1 POPC:POPG:Rh-PE:NBD-PE were mixed at a ratio of 9:1 unlabeled:labeled, unless otherwise indicated. Protein (5 μM), whether that be the WT or each mutant, and 100 μM LUVs were mixed to achieve a protein:lipid ratio of 1:20 in HMA buffer. All experiments were carried out in a Corning Costar black-walled, clear-bottomed 96-well plate with 150 μL per well. Fluorescence was recorded at room temperature (~23°C) on a SpectraMax M5 microplate reader (Molecular Devices, San Jose, CA) with excitation and emission wavelengths recorded at 460 and 538 nm, accordingly, with a cutoff at 530 nm. Percent fusion was calculated from the equation Fusion % $= \frac{F_M - F_o}{F_T - F_0} \times 100\%$, where F_M is the measured fluorescence at lysosomal pH (4.0), F_o is the background fluorescence at physiological pH (7.4), and F_T is the total fluorescence after 1% Triton X-100

was added to the system to cause complete vesicle disruption. Acidification was carefully controlled through the addition of 1 M HCl, as verified by a pH titration of the HMA buffer, which is linear from pH 3.0 to 8.0. Controls containing no protein, merely LUVs and HMA buffer, were run alongside each experimental condition, subjected to the same equation, and subtracted from the final values with error propagated from the SEM.

CD spectroscopy

To prepare the protein, either WT or each mutant, for CD experiments, the sample was diluted with 15 mL of CD buffer, then concentrated down to 1 mL with a 15 mL capacity, 3 kDa MWCO Amicon Ultra Centrifugal Filter (Sigma-Aldrich). This was repeated five times to fully exchange the sample from HMA buffer to CD buffer, where the latter is 10× diluted to prevent noisy signals from excess salt concentrations. CD measurements were conducted at room temperature (∼23°C) on a Jasco J-810 spectrometer (Jasco, Easton, MD) utilizing a quartz cuvette with a 2 mm pathlength. Samples were comprised of 8 μM of WT or each mutant and 0.8 mM SUVs in CD buffer for a ratio of 1:100 protein:lipid. Spectra were collected from 260 to 198 nm with a step size of 1 nm at 20 nm/min and averaged over three accumulations. Control measurements were taken for 0.8 mM SUVs alone in CD buffer. CDToolX was employed to subtract the control measurements from the respective sample, then the spectra were smoothed via the software (63). Spectra were converted to mean residue ellipticity units. Helical percentages were calculated from the mean residue ellipticity of a given spectrum at 222 nm (θ_{222}) from the equation $F_H = \frac{\theta_{222} - \theta_C}{\theta_H - \theta_C}$ where θ_C = 2220–53T, $\theta_H = (250T–44{,}000) \times (1 - \frac{3}{n})$, T is the temperature in Celsius, and n is the number of residues in the protein (39,42,64,65).

ITC

The desired protein, either WT or each mutant, was added to a 0.5–3 mL, 3.5 kDa MWCO Slide-A-Lyzer dialysis cassette (Thermo Fisher Scientific) and dialyzed against 2 L of ITC buffer at 4°C for a minimum of 4 h. The protein was subsequently removed from the dialysis cassette, spun down for 15 min at 20,000 × *g* in an Eppendorf 5425 microcentrifuge, and transferred to a clean microcentrifuge tube. The protein was then degassed for 8 min at room temperature in a Microcal Thermovac alongside 20 mM of 65:35 POPC:POPG SUVs that were in the ITC buffer. The microplate reader protocol for the Pierce Bicinchoninic Acid protein assay kit (Thermo Fisher Scientific) was employed to determine the protein concentration of the resulting sample, which was typically ∼15–20 μM, then ran on the ITC. A Malvern VP-ITC microcalorimeter (Malvern Panalytical, Malvern, England) was used for all ITC measurements where the sample cell was filled with the degassed protein and the injection syringe with the degassed SUVs. A total of 41 injections were performed at 23°C with the reference power set to 5 μcal/s, stirring speed of 310 rpm, and filter period of 2 s. Each injection was set to 7 μL with a 16.8 s duration, except for the first injection which was 2 μL with a 4.8 s duration. Al processing was conducted through NITPIC (66) and SEDPHAT (67) with final figures created via GUSSI (68). At least two titrations were performed for each condition to deduce the average dissociation constant (K_d) and SD, which are presented on each isotherm.

MD simulations

The initial structural model of the LASV FD was taken from the Protein Data Bank (PDB: 7PUY (16)) using residues G^{260}-N^{295}. The terminal amino acids were capped using the acetylated N-terminal (ACE) and methyl-amidated C-terminal (CT3) blocking groups (50,69,70). A series of membrane systems composed of POPC and either POPG, POPS, or BMP were built using CHARMM-GUI *Membrane Builder* (Table S1) (71–73). All simulations were performed with all-atom CHARMM36(m) force fields for the protein and lipids (74,75), whereas TIP3P parameters were used for water with sodium and chloride ions added to reach a neutral concentration of 150 mM NaCl (76). The pH of the system was set to 4.0 with all aspartic acid (D^{268}) and glutamic acid (E^{270}, E^{287}, and E^{289}) residues protonated. The temperature and pressure of the system were set to 318.15 K and 1 bar, accordingly. The protonated LASV FD was initially translated approximately 20 Å above the membrane. The initial system size of each replica was 100 × 100 × 128 Å to accommodate all initial components, yielding a total of roughly 130,000 atoms per replica. MD simulations (2 μs) were performed for five replicas for each of the three membrane systems, leading to a total of 15 independent simulations. All simulations were conducted using OpenMM (77) with the inputs generated by Membrane Builder (78,79). Analyses were performed using MDanalysis (80,81) and models were visualized in VMD, version 1.9.4 (82).

RESULTS

A conformational change of the LASV FD at a low pH results in its FL adopting a helix and associating with the host cell membrane

We utilized a series of solution NMR spectroscopy backbone experiments to investigate the structure of the LASV FD in both its pre- and postfusion states. For the prefusion state, G^{260} and T^{261} could not be assigned, due to their inherent flexibility as terminal residues, whereas I^{286} of the postfusion backbone was not assigned due to peak ambiguity, correlating with 95% pre- and 97% postfusion backbone assignment, respectively. A comparison of ^{1}H-^{15}N HSQC spectra of the FD in solution at physiological pH (Fig. 2 *A*) and with acidic bicelles at lysosomal pH (Fig. 2 *B*) revealed moderate chemical shift perturbations. This perturbation could arise from potential environmental and/or conformational differences between the two conditions, although a previous CD study showed conformational changes upon pH changes in the presence of acidic bicelles. To further distinguish, we determined the secondary structure of the LASV FD by generating a CSI from the pre- and postfusion backbone assignments. The chemical shift of the alpha carbons (Cα) for all residues within the FD was compared with that of a random coil where a positive value greater than one indicates a helix, a negative value indicates a sheet, and a value close to zero indicates a random coil (60). The CSI suggests an almost completely random coil secondary structure for both the pre- and postfusion states (Fig. S4 *A*). The same trend was observed for the CSI of the amide (N) (Fig. S4 *B*), amide hydrogen (H) (Fig. S4 *C*), and carbonyl carbons (CO) (Fig. S4 *D*). While the CSI of the prefusion state agreed with the literature (16,17,42), the lack of helical structure in the postfusion state was unexpected. Subsequently, we employed TALOS+ to yield a more in-depth secondary structural analysis of the LASV FD from our backbone assignment and provide predictions for the residues with missing peaks in either state. This analysis further supported the notion that the prefusion state of the LASV FD is a random coil (Fig. 2 *C*, *red*; Table S2). In the postfusion, however, it was revealed

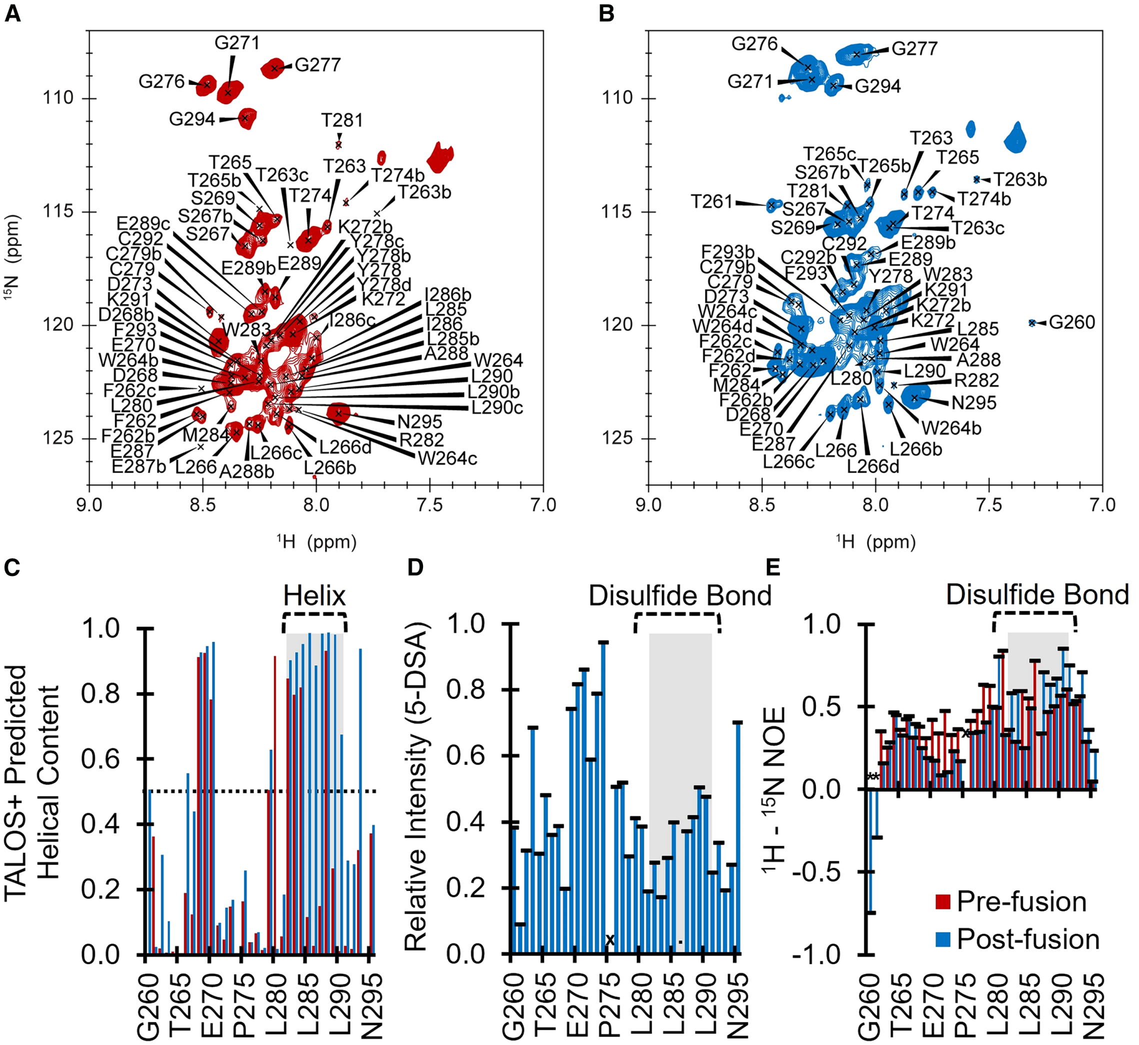

FIGURE 2 The LASV FD undergoes a conformational change from the pre- to postfusion state and associates with the membrane via its FL. (*A* and *B*) ^{1}H-^{15}N HSQC spectra highlight a multitude of peaks that shift from the (*A*) pre- to (*B*) postfusion state. (*C*) An in-depth secondary structure analysis from the pre- (*red*) and postfusion (*blue*) backbone assignment via TALOS+ provides predicted dihedral angles and suggests a helix is formed in the FL from R^{282} to L^{290} as values are consistently above 0.5. (*D*) Addition of 8 mM 5-DSA into the system containing the LASV FD resulted in quenching of the FL, but not the FP. (*E*) ^{1}H-^{15}N NOE indicates that the FD becomes more restricted from the pre- (*red*) to postfusion (*blue*) state with the FL being particularly limited. All measurements were carried out on ~500 μM of the LASV FD in 300 μL of 25 mM Na_2HPO_4, 100 mM NaCl (pH 7.0) at 20°C (prefusion) or pH 4.0 with acidic bicelles, q = 0.5 at 45°C (postfusion). Proline residues and other residues that could not be assigned for both states are marked with an X, whereas residues that could not be assigned for the pre- or postfusion state are marked with an asterisk (*) or period (.), accordingly. The most populated conformer (*a*) is the residue label, whereas the second (*b*), third (*c*), and fourth (*d*) most populated conformers are shown. The disulfide bond (C^{279} and C^{292}, *dashed line*) and helix (R^{282}-L^{290}, *transparent gray box*) are indicated. Error bars shown are propagated from signal-to-noise ratio.

that an α helix of approximately three turns exists within the FL for residues R^{282}-L^{290} (Fig. 2 *C*, *blue*; Table S3). Additionally, TALOS+ indicated a short helix potentially formed for residues S^{267}-E^{270}, but this would be unlikely due to the high propensity of destabilizing amino acids (i.e., S and D) and lack of hydrophobic amino acids. This corresponds well with the literature where it has been shown that the LASV FD adopts a helical structure with ~13 residues involved in the postfusion state, as previously shown via CD spectroscopy (42). Notably, the internal disulfide bond is juxtaposed to this potential helix and formed correctly in both states. This is evidenced by the β carbon (Cβ) chemical shifts being greater than 35 ppm (Fig. S5) in both states, indicating oxidized cysteines, and a lack of free thiols under native conditions (Fig. S6). Given its proximity to the helix, the disulfide bond potentially restricts the length of the helix in the postfusion state. Altogether, it is indicated that the LASV FD undergoes a conformational change from a random coil in the prefusion state to a helix in the postfusion state, which forms for residues R^{282}-L^{290} within the FL and can thus associate with the host cell membrane during fusion.

To further elucidate the depth of the LASV FD in the pre- and postfusion states, we utilized paramagnetic relaxation enhancement (PRE) experiments with the paramagnetic probes Gd-DTPA, 5-DSA, and 16-DSA. The water-soluble Gd-DTPA causes signal quenching of solvent-exposed residues, whereas 5-DSA and 16-DSA quench signals for residues located proximally to the head and tail groups, respectively. In the prefusion state, the entire LASV FD (0.420 ± 0.021) experienced virtually the same amount of quenching as Gd-DTPA was titrated into the solution (Fig. S7; Table S4). The maximum quenching achieved for the LASV FP (0.458 ± 0.027) and FL (0.431 ± 0.025) were nearly identical, indicative that both were solvent exposed. In the postfusion state, however, we found that the FP was solvent exposed, whereas the FL was inserted into the lipid headgroup of the membrane. The overall quenching of the FD in its postfusion state (Gd-DTPA = 0.222 ± 0.026, 5-DSA = 0.439 ± 0.037, and 16-DSA = 0.620 ± 0.027) suggests that it associates with the membrane in a shallow manner. To be more specific, the relative intensities from the Gd-DTPA titration were 0.126 ± 0.022 and 0.317 ± 0.038 for the FP and FL, accordingly (Fig. S8 *A*). A reverse trend was witnessed for the 5-DSA titration where the relative intensities of the FP were increased (0.530 ± 0.068) in comparison to the FL (0.353 ± 0.034) (Fig. 2 *C*). Furthermore, residues D^{268}-T^{274} within the FP were significantly quenched by Gd-DTPA, but not 5-DSA, whereas residues R^{282}-L^{285} and K^{291}-F^{293} within the FL were quenched by 5-DSA, but not Gd-DTPA. The relative intensities from the titration of 16-DSA were nearly identical for the FP (0.623 ± 0.046) and the FL (0.620 ± 0.039) (Fig.S8 *B*). Taking all of the PRE data into consideration, we believe that the FD associates with the membrane in a relatively shallow manner. The FL forms a boat-like helical structure for residues R^{282}-L^{290} that is embedded just below the lipid headgroups. In contrast, the FP, mainly D^{268}-T^{274}, does not insert itself into the membrane and is more solvent exposed in both states.

We also investigated the protein dynamics of the LASV FD in the pre- and postfusion states using ^{1}H-^{15}N NOEs and R_1 and R_2 relaxation rates (Table S5). The overall relaxation times of the FD in its prefusion state (^{1}H-^{15}N NOEs = 0.448 ± 0.027 s [Figs. 2 *E* and S10, *red*], and rates, R_1 = 1.735 ± 0.091 s^{-1} [Fig. S9 *A*, *red*], and R_2 = 4.941 ± 0.356 s^{-1} [Fig. S9 *B*, *red*]) agree relatively well with the dynamics of a random coil (44,83). In particular, the FP was slightly more flexible than the FL in the prefusion state, as denoted by the ^{1}H-^{15}N NOEs of 0.369 ± 0.018 and 0.497 ± 0.046 s, accordingly. In turn, for our relaxation experiments, we found that the FP had similar R_1 relaxation rates (1.750 ± 0.159 s^{-1}) to the FL (1.651 ± 0.062 s^{-1}), but lower R_2 relaxation rates (4.137 ± 0.550 s^{-1}) than the FL (5.708 ± 0.501 s^{-1}), further suggesting that the FP is somewhat more dynamic than the FL in the prefusion state. Opposingly, the relaxation times of the FD in its postfusion state (^{1}H-^{15}N NOEs = 0.419 ± 0.038 s [Figs. 2 *E* and S10, *blue*], and rates, R_1 = 1.305 ± 0.040 s^{-1} [Fig. S9 *A*, *blue*], and R_2 = 6.109 ± 0.683 s^{-1} [Fig. S9 *B*, *blue*]) are suggestive of a more restricted structural conformation. In the postfusion state, the FP had much lower ^{1}H-^{15}N NOEs (0.253 ± 0.034 s), particularly residues D^{268}-T^{274} than the FL (0.564 ± 0.048 s), indicating that the FP continued to be more flexible than the FL, but to a greater extent than in the prefusion state. Interestingly, the FP again had similar R_1 relaxation rates (1.280 ± 0.036 s^{-1}) to the FL (1.339 ± 0.078 s^{-1}), but lower R_2 relaxation times (4.426 ± 0.390 s^{-1}) than the FL (7.947 ± 1.224 s^{-1}), further correlating with the FP being more dynamic than the FL in the postfusion state. Taken together, these findings suggest that the two regions of the LASV FD have different dynamic properties in both states. While both are relatively flexible in the prefusion state, the FL becomes especially restricted in the postfusion state due to its association with the membrane via residues R^{282}-L^{290}, which are in a helical conformation, whereas the FP becomes more dynamic, chiefly for residues D^{268}-T^{274}, likely due to its increased exposure to the solvent.

Multiple conformations are adopted by certain residues in the pre- and postfusion states of the LASV FD, as evidenced by a particularly dynamic leucine residue

When performing the backbone assignment, we noticed that multiple residues within the FD appeared to slowly sample several conformations in both the pre- and postfusion states. We observed numerous ^{1}H-^{15}N strips with the same chemical shifts for the Cα, Cβ, and CO, but different hydrogen and nitrogen shifts, of the particular residue (*i*). Additionally, in all of these strips, the chemical shifts of the Cα, Cβ, and CO were identical for the preceding residue (*i – 1*) as well. This suggested that a single residue was slowly exchanging between different conformations on a timescale that could be captured by the NMR as distinctive peaks, i.e., the millisecond to second scale (84–86). The first residue was what we believed to be residue L^{266} located in the middle of the FP, which appeared to have distinct conformations in both the pre- and postfusion states. In particular, we observed four different ^{1}H-^{15}N strips with a $C\alpha_i$ of ∼55 ppm and $C\beta_i$ of ∼42 ppm, a trademark of leucine residues, and $C\alpha_{i-1}$ of ∼55 ppm, $C\beta_{i-1}$ of ∼62 ppm, and Cβ of ∼69 ppm, typical of threonine residues for both the pre- (Fig. S11) and postfusion states (Fig. S12). Given the sequence of the LASV FD, this left one residue, L^{266}, which is the only leucine residue to be preceded by a threonine residue, T^{265}, as the most likely candidate for this assignment.

Nonetheless, before we delved too far into assigning an unknown number of conformers, we aimed to determine if there was any merit behind our notion that the LASV FD was adopting multiple conformations at a residue-specific

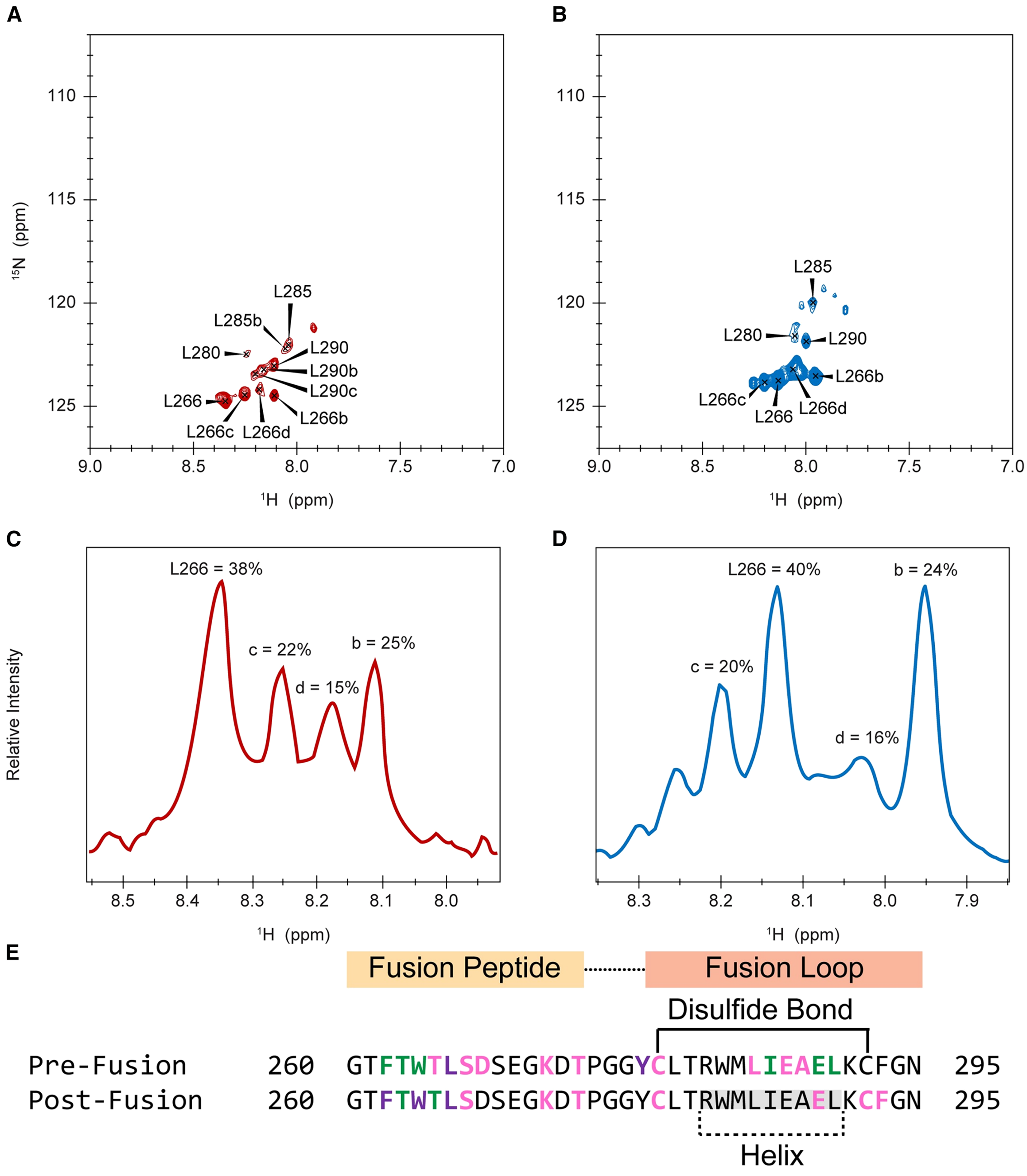

FIGURE 3 Multiple conformations are adopted by L^{266} in the LASV FD. (*A* and *B*) ^{1}H-^{15}N-Leu HSQC spectra indicate a multitude of conformers exist for leucine residues in the (*A*) pre- and (*B*) postfusion state. (*C* and *D*) Each L^{266} conformer is occupied for different percentages of time in the (*C*) pre- and (*D*) postfusion state. All measurements were carried out on ~1000 μM of the LASV FD in 300 μL of 25 mM Na_2HPO_4, 100 mM NaCl (pH 7.0) at 20°C (prefusion) or pH 4.0 with acidic bicelles, q = 0.5 at 45°C (postfusion). The most populated conformer (*a*) is the residue label, whereas the second (*b*), third (*c*), and fourth (*d*) most populated conformers are indicated as such. (*E*) Numerous residues throughout the FP and FL sample multiple conformations in the prefusion state (*top*), but only the FP continuing to do so in the postfusion state (*bottom*). Residues with two conformers (*pink*), three conformers (*green*), and four conformers (*purple*) and the helix (*gray box*) formed in the postfusion state are shown.

level. Since we were confident that L^{266} had four distinct populations and several other leucine residues are distributed throughout the LASV FD, we decided to selectively ^{15}N label all leucine residues to further probe the existence of other multiple conformers and their population. A ^{1}H-^{15}N HSQC of the isotopically labeled ^{15}N-Leu FD at physiological pH (Fig. 3 *A*) and lysosomal pH with acidic bicelles (Fig. 3 *B*) correlated well with that of the entirely labeled FD. When we overlaid the ^{1}H-^{15}N-Leu HSQC with the ^{1}H-^{15}N HSQC and then looked at the corresponding strips in the different backbone experiments, we were led to the same ^{1}H-^{15}N strips that we had previously identified as L^{266}. This affirmed our notion that the observed strips with the same shifts arose from the same L^{266} residue and

not a different residue with similar chemical shifts. From the ^{1}H-^{15}N HSQC, we deduced the percentage of each population and assigned accordingly by taking the integration of each conformer. In the prefusion state, the first (*a*), second (*b*), third (*c*), and fourth (*d*) most populated conformer of L^{266} existed at 38, 25, 22, and 15%, correspondingly (Fig. 3 *C*), which was comparable with the postfusion state at 40, 24, 20, and 16%, respectively (Fig. 3 *D*). Here, labels (*a*), (*b*), (*c*), and (*d*) are assigned independently for each residue based on peak intensity, with (*a*) corresponding to the most populated conformer for that specific residue. These labels do not represent globally shared conformational states, but instead serve as residue-specific identifiers of multiple observable populations. The PRE values and dynamics of L^{266} were somewhat analogous between each conformation, aside from the d conformer, indicative that each conformer was similarly exposed to the environment and flexible (Fig. S13). The difference observed here could be due to the d conformation being infrequently adopted and, thus, on a timescale that cannot be adequately captured by these experiments. Furthermore, we effectively applied this approach to the other leucine residues, i.e., L^{280}, L^{285}, and L^{290}, allowing us to identify conformations for other leucine residues. We found two conformations for L^{285} and three for L^{290} in the prefusion state, but no additional conformers for the other leucine residues in the postfusion state. For L^{285}, the a conformation was occupied 68% of the time, whereas the b conformer was 32%. The a conformer of L^{290} was populated 53% of the time, b conformer was 34%, and c conformer was 13%. These findings indicate that multiple residues within the LASV FD adopt different populations, which can be different between the pre- and postfusion states.

We next turned our attention back to the entire ^{15}N-labeled FD to further elucidate residues with multiple populations (Table S6). In the prefusion state, we found eight other residues in the LASV FD with two different conformations (T^{265}, S^{267}, D^{268}, K^{272}, T^{274}, C^{279}, E^{287}, and A^{288}), five with three conformations (F^{262}, T^{263}, W^{264}, I^{286}, and E^{289}), and one with four conformations (Y^{278}). Thus, a total of 17 residues (F^{262}, T^{263}, W^{264}, T^{265}, L^{266}, S^{267}, D^{268}, K^{272}, T^{274}, Y^{278}, C^{279}, L^{285}, I^{286}, E^{287}, A^{288}, E^{289}, and L^{290}) with multiple conformations were identified in the prefusion state, equating to approximately 50% of the LASV FD having numerous populations (Fig. 3 *E*, *top*). The FP exchanged populations slightly more than the FL with 9 out of 15 residues (60%) shown to have different conformers, as opposed to the 7 out of 17 residues (41%) in the FL. In the postfusion state, however, the fraction of the LASV FD having different conformations decreased to 12 residues (33%) with a bulk majority of the same residues in the FP, but not the FL, continuing to exchange (Fig. 3 *E*, *bottom*). Here, only four residues (24%) of the FL had two different conformers in the postfusion state, including C^{279} and E^{289}, which occurred in the prefusion state as well, and newly identified C^{292} and F^{293}. Nonetheless, all residues identified in the FP to have multiple conformers in the prefusion state, except D^{268}, continued to do the same in the postfusion state. Intriguingly, while S^{267}, K^{272}, and T^{274} maintained two conformations, T^{263} and T^{265} now had three conformations, whereas F^{262} and W^{264} joined L^{266} in having four conformations. All of the residues identified to have multiple conformations occupied each population to a varying degree in the pre- and postfusion state (Table S6). On average, the a, b, c, and d conformers were populated 67 ± 19%, 24 ± 12%, 16 ± 6%, and 11 ± 6% of the time, accordingly, in the prefusion state. This remained relatively the same in the postfusion state with the a, b, c, and d conformations adopted 67 ± 17%, 25 ± 11%, 16 ± 8%, and 10 ± 6% of the time, correspondingly.

Notably, the predicted dihedral angles of each conformer are relatively similar to each other in both the pre- (Table S7) and postfusion (Table S8) states, which led us to question where these alternative conformers arose from. Thus, we employed site specific, ^{19}F labeling of the LASV FD with 4-fluoro-D,L-phenylalanine, which has a fluorine attached at the *para* position of the aromatic. The rationale here is that fluorine is highly sensitive to its chemical environment, which allows us to monitor the side chain for chemical shifts undergone that occur in response to its surroundings (87). Notably, there are two phenylalanine residues in the LASV FD: one at the N-terminal of the FP (F^{262}) and one at the C-terminal of the FL (F^{293}) (Fig. 1). Since F^{262}, but not F^{293}, appeared to sample numerous conformers in both the pre- and postfusion state, we decided to selectively ^{19}F-label F^{262}. To accomplish this, we introduced a single mutation into F^{293} (i.e., $F^{293}W$), such that only F^{262} would be labeled and analyzed. Given our previous data, we expected to see three peaks in the prefusion state and four peaks in the postfusion state—accounting for one peak per conformer observed. Interestingly, our ^{19}F experiments revealed that the side chain of F^{262} experienced two additional conformers in the prefusion state (Fig. S14 *A*) for a total of six conformers and one additional conformer in the postfusion state (Fig. S14 *B*) for a total of five conformers. This suggests that these alternative conformers are due to the side chains of a given residue sampling different states. Altogether, it is evident that numerous residues throughout the LASV FD populate different conformers in the prefusion state, whereas only the FP continues to sample multiple conformations in the postfusion state, likely because it is still solvent exposed, but the FL becomes affixed when it associates with the membrane.

A net negative charge akin to the lysosomal compartment correlates with increased LASV FD-initiated fusion, which is not influenced by positively charged residues

The LASV FD has been established to preferentially initiate fusion with the lysosomal membrane, which has a high

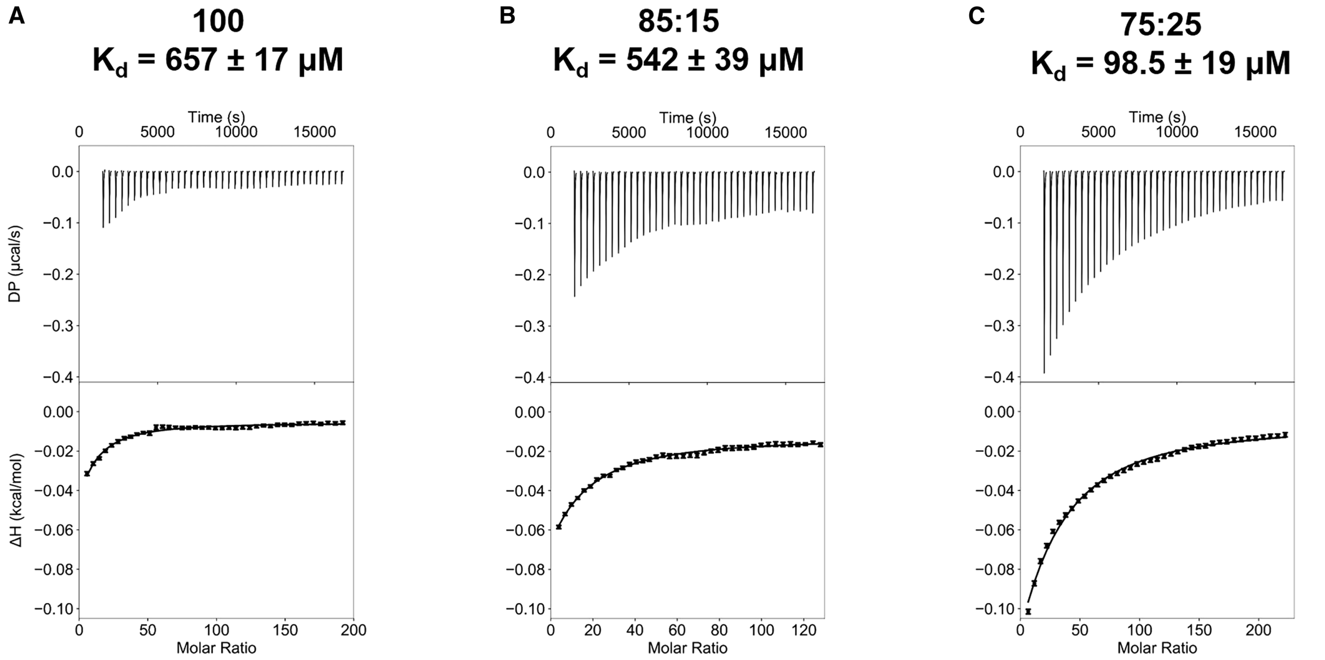

FIGURE 4 A net negative charge akin to the lysosomal membrane is important for the function of the LASV FD. (*A*) 100 POPC, (*B*) 85:15 POPC:POPG, and (*C*) 75:25 POPC:POPG were titrated into the protein. Displayed ratios are in terms of POPC:POPG. All ITC experiments were conducted in 10 mM NaOAc, 100 mM NaCl (pH 4.0) with vesicles titrated into the protein. Dissociation constants (K_d) are displayed above the respective isotherm. An exemplary isotherm for LASV FD into 65:35 POPC:POPG is not shown as it has been published previously.

propensity of anionic lipids that can impact fusion—as observed with other class one fusion viruses (45,88–93). We first aimed to determine if the LASV FD has a specificity for anionic lipids by performing a proof-of-concept titration with various molar ratios of POPC:POPG, to provide a simple mimic of the lysosomal membrane, where POPG is a simple anionic lipid commonly used in model systems. We observed that the LASV FD had a weak affinity for vesicles that were completely zwitterionic and neutral (100 POPC) with a K_d of 657 ± 17 μM (Fig. 4 *A*). Minimal affinity was noted for vesicles with low concentrations of anionic lipids and negative charge (85:15 POPC:POPG) as denoted by the slight decrease in K_d to 542 ± 39 μM (Fig. 4 *B*). In contrast, there was a drastic decrease in the K_d to 98.5 ± 19 μM when vesicles with an anionic lipid ratio and net negative charge approaching that of the lysosomal membrane were introduced (75:25 POPC:POPG), correlating with an increased affinity for the vesicles (Fig. 4 *C*). This increase in affinity for the vesicles continued as the concentration of anionic lipids and, thus, net negative charge was increased to match that of the lysosomal compartment with a K_d of 11.7 ± 0.8 μM previously established for vesicles comprised of 65:35 POPC:POPG (42). More specifically, a nearly 50-fold increase in affinity was witnessed between 85:15 POPC:POPG and 65:35 POPC:POPG vesicles, whereas an 8-fold increase was noted when the latter was compared with 75:25 POPC:POPG vesicles, indicating that anionic lipids influence LASV FD-initiated fusion.

To investigate the structural and functional impact of the different POPC:POPG vesicles on the LASV FD, we employed CD spectroscopy and a FRET-based lipid mixing assay, respectively. We found that the helical propensity of the FD was dependent upon the concentration of POPG present (Fig. 5 *A*). The LASV FD appeared to exist in a predominantly random coil conformation in vesicles comprised of 100 POPC and 85:15 POPC:POPG, as delineated by the single dip at 200 nm. As the concentration of anionic lipids and, thus, net negative charge, was increased to 75:25 POPC:POPG and 65:35 POPC:POPG, the secondary structure of the LASV FD transitioned to be mainly helical, indicated by the double dip at 208 and 222 nm, with similar helical content at 33 and 35%, correspondingly. This correlated with an exponential increase in fusion, as expected given that the helical conformation of the LASV FD has been established to be its fusogenic form (Fig. 5 *B*). In particular, we witnessed no fusion in 100 POPC vesicles. Minimal fusion was noted in 85:15 POPC:POPG vesicles, which was followed by a negligible increase in 75:25 POPC:POPG vesicles. In contrast, fusion readily occurred in vesicles comprised of 65:35 POPC:POPG. Despite the FD having comparable helical propensity in 75:25 POPC:POPG and 65:35 POPC:POPG, a fivefold increase in fusion was observed between 75:25 POPC:POPG and 65:35 POPC:POPG vesicles. These findings suggest that the LASV FD is sensitive to the concentration of anionic lipids present with optimal fusion occurring when a net negative charge akin to the lysosomal membrane is present.

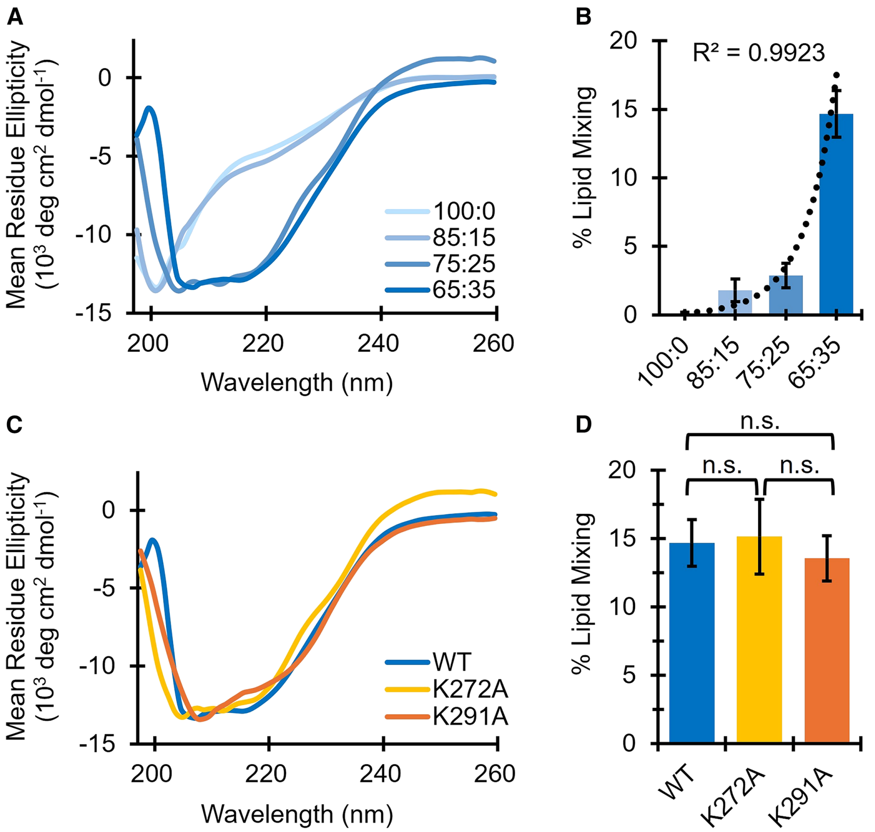

FIGURE 5 Structure and function of the LASV FD is reliant on the overall negative charge, which is not mediated by positively charged lysine residues. (*A*) Global secondary structure is dependent on the zwitterionic:anionic lipid ratio with a helical structure adopted only once a net negative charge akin to the lysosomal membrane was achieved. (*B*) Exponential relationship between increased concentrations of POPG and fusion with optimal fusion occurring in 65:35 POPC:POPG ($n \geq 16$). (*C*) In vesicles comprised of 65:35 POPC:POPG, all lysine mutants (K272A, *yellow*; K291A, *orange*) had similar secondary structure to WT (*blue*). (*D*) Mutation of the lysine residues had no consequential impact on fusion ($n \geq 15$). Student's *t*-test assuming unequal variances used to calculate the *P* value; n.s., not significant. Displayed ratios are in terms of POPC:POPG. The error bars shown are propgated from standard error of the mean (SEM).

We next asked if this preference for a high propensity of negatively charged lipids was mediated by an ionic interaction. A sequence alignment revealed that there are three positively charged residues within the LASV FD: K^{272}, located in the FP, and R^{282} and K^{291}, found in the FL (Fig. 1). Notably, K^{272} is not conserved, R^{282} is moderately conserved, and K^{291} is completely conserved. Single point mutations were generated to alter the positively charged side chains of residues K^{272} and K^{291} into a chemically inert alanine (i.e., K^{272}A and K^{291}A) instead. Mutagenesis of R^{282} was not performed as it has previously been established to be a part of a salt bridge that is important to LASV FD-initiated fusion (42). Surprisingly, the global secondary structure of the LASV FD remained relatively unchanged when either residue was mutated (Fig. 5 *C*). To be more specific, the helical content of K^{272}A and K^{291}A was 32 and 33%, respectively, which was akin to that observed in WT (35%) (42). There were no significant impacts on function with both K^{272}A and K^{291}A having similar amounts of fusion as WT (Fig. 5D). This corresponded to a high affinity for the membrane with a K_d of 19.8 $\pm$ 1.0 μM for K^{272}A (Fig. S15 *A*) and 19.2 $\pm$ 1.2 μM for K^{291}A (Fig. S15 *B*). Thus, the observed preference for a negatively charged lipid does not appear to be mediated by an ionic interaction between the membrane and the LASV FD.

The LASV FD has an enhanced interaction with BMP, an anionic and conical lipid prevalent within the lysosomal membrane

We next sought to deduce if anionic lipids with physiological relevance to eukaryotic cells, chiefly those found in the lysosomal compartment, have a particular effect on the LASV FD. The lysosomal membrane has a high composition of lipids known to influence fusion, namely BMP, phosphatidylethanolamine (PE), and phosphatidylserine (PS). We thus conducted experiments with 65:35 POPC:X, where X was either BMP, POPE, or POPS. Notably, BMP has both a negatively charged lipid headgroup, similar to POPS, and an inverted cone shape, akin to POPE, which is not captured when studying POPS and POPE alone. As such, we also investigated the effect that vesicles comprised of 65:17.5:17.5 POPC:POPS:POPE had on the function of the LASV FD. We demonstrate that the FD only has helical content when in the presence of POPC:BMP, which was comparable with that observed in POPC:POPG (Fig. 6 *A*). More specifically, even at 2$\times$ higher concentrations of POPE (i.e., 65:35 POPC:POPE vs. 20% PE in the lysosomal compartment) or 10$\times$ higher concentrations of POPS (i.e., 65:35 POPC:POPS vs. 2% PS in the lysosomal compartment) than physiologically relevant, the LASV FD seemed to exist as a random coil structure, as the presence of a

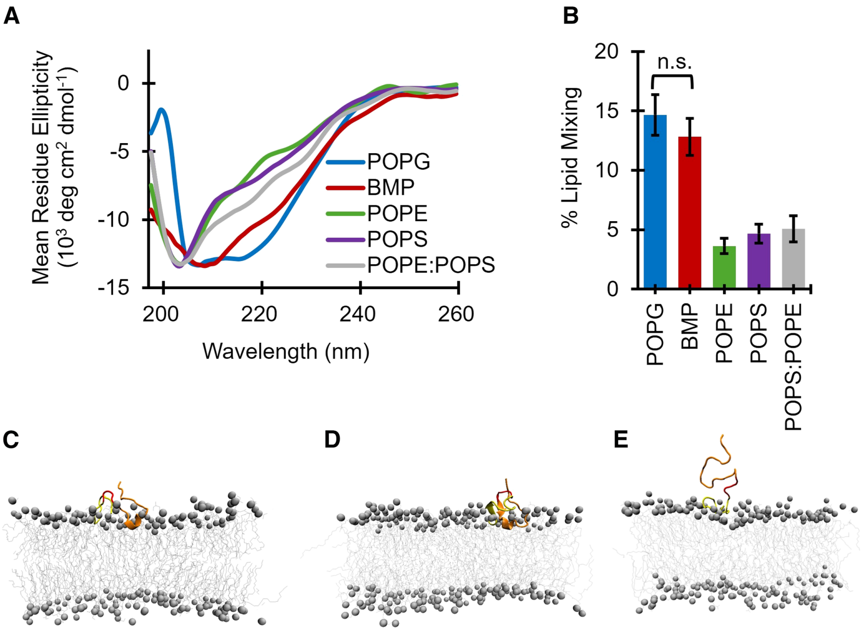

FIGURE 6 Anionic lipids, chiefly BMP, impact the structure and function of the LASV FD. (*A*) Global secondary structure was influenced by the type of physiologically relevant lipid present with coil character observed in all lipids (POPE, *green*; POPS, *purple*; POPS:POPE, *gray*), except BMP (*red*), which appeared similarly to POPG (*blue*). (*B*) Fusion of the LASV FD was dependent on the lipid present with optimal fusion occurring in BMP ($n \geq 12$). Student's *t*-test assuming unequal variances used to calculate the *P* value. All results were significant unless designated; n.s., not significant. (*C* and *E*) After 2 μs, the LASV FD adopts a helix in its FL (*orange*) that associates with the lipid headgroup, whereas the FP (*yellow*) remains solvent exposed in membranes comprised of (*C*) POPC:POPG and (*D*) POPC:BMP, but not (*E*) POPC:POPS. The error bars shown are propagated from standard error of the mean (SEM).

deep minimum near 200 nm was witnessed. This remained true in the presence of POPS and POPE together (POPC:POPS:POPE), as the FD still existed in a largely random coil conformation. However, in BMP, dips at 208 and 222 nm were observed, indicative of a helix. While the 208 nm intensity was comparable with that observed in POPC:POPG, there was a slight decrease in the 222 nm intensity, correlating with a decrease in helical content to 30%. This could be attributed to the tendency of the helix to be slightly decreased as a result of subtle alterations in its environment.

Since there is a direct link between the structure and initiation of fusion, we aimed to decipher how these physiologically relevant lipids impacted the function of the LASV FD. We witnessed a negligible decrease in binding affinity and fusion when the FD was in POPC:BMP as opposed to POPC:POPG (Figs. 6B and S16 *A*). The FD had a K_d of 25.2 $\pm$ 3.7 μM in vesicles comprised of BMP, which was twofold higher than that established in POPC:POPG; however, there was no statistically significant difference in fusion. In contrast, when the LASV FD was in vesicles comprised of POPC:POPE, POPC:POPS, or POPC:POPS:POPE, we witnessed a nearly identical reduction in binding affinity and fusion when compared with POPC:BMP and POPC:POPG. The FD had a minimal affinity for vesicles comprised of POPC:POPE, as denoted by the drastic increase in the K_d to 606 $\pm$ 155 μM (Fig. S16 *B*), which led to an over threefold decrease in fusion. This remained unchanged in vesicles containing POPC:POPS or POPC:POPS:POPE as the K_d was 453 $\pm$ 32 μM (Fig. S16 *C*) and 415 $\pm$ 21 μM (Fig. S16 *D*), respectively, with similar amounts of fusion. It could be argued that studying POPS and POPE together did not truly encapsulate the double unsaturation and anionic headgroup at concentrations similar to BMP, which is why vesicles comprised of 65:17.5:17.5 POPC:POPS:POPE did not have similar levels of LASV FD-initiated fusion witnessed in 65:35 POPC:BMP. However, we saw no significant difference in the ability of the LASV FD to initiate fusion in lipids with the same headgroup, but different tail saturations (Fig. S17). Thus, the functionality of the FD has a clear preference on the type of lipid present, namely BMP.

MD simulations were employed to visualize the interaction of the FD with various membranes, specifically the degree to which the helical structure existed and, thus, penetrated the membrane. Three membrane systems were simulated, including POPC:POPG, POPC:BMP, and POPC:POPS. Systems with POPC:POPE and POPC:POPS:POPE were not simulated since the FD has a clear preference for anionic lipids and there was no significant difference experimentally in the structure or function when compared with POPS. We provide evidence that the LASV FD could associate with membranes comprised of POPC:POPG (Fig. 6 *C*; Video S1) and POPC:BMP (Fig. 6 *D*; Video S2). Our membrane packing results also show that, in the presence of FD, BMP and POPG show larger lateral membrane size, especially when the membrane composed of BMP is highly perturbed by FD (Fig. S19). In addition, helical content was observed in POPC:POPG (Fig. S18 *A*) and POPC:BMP (Fig. S18 *B*), especially in the FL for residues R^{282}-L^{290}, but more residues were frequently involved in the formation of the helix in POPC:POPG than POPC:BMP, supporting our experimental CD data. The helix inserted itself just below the lipid headgroup in both the POPC:POPG and

POPC:BMP systems. On the contrary, the FD did not associate with the POPC:POPS membrane (Fig. 6 *E*; Video S3) and remained largely random coil over the entire course of the simulation (Fig. S18 *C*). This finding was evident in contact analysis that the FD overall interacts with the membrane in a predominantly surface-associated manner. Comparing three different membranes, the FD has stronger contact with membranes containing BMP and POPG, which in contrast to contact with POPS which was relatively lower (Fig. S20). Put together, our results indicate that anionic lipids in the lysosomal membrane, chiefly BMP, are imperative for the LASV FD to interact with the membrane and initiate fusion.

DISCUSSION

Infection with LASV results in LF, a severe hemorrhagic fever with no FDA-approved therapeutic options. Delivery of LASV's genetic material is achieved by the FD, which undergoes a conformational change to become helical and initiate fusion at the lysosomal compartment. In other class one fusion proteins, the FD is either an FP or FL, but in LASV the FD has both an FP and FL in tandem (Fig. 1). Previous work has demonstrated that the LASV FD preferentially initiates fusion at the lysosomal membrane, at which point it adopts a fusogenic, helical conformation (42). However, the structural and functional characteristics governing this interaction remain poorly understood, especially the location of the helix and the role of particular lipids in the lysosomal membrane. Here, we reveal that the FL of the LASV FD forms a helix to associate with the membrane, whereas the FP remains solvent exposed and samples multiple conformations. We indicate that the LASV FD has a preference to initiate fusion with anionic lipids, namely BMP, which is not mediated by an ionic interaction with lysine residues located in the FP or FL. These observations highlight the distinct roles of the FP and FL in facilitating membrane fusion and suggest a unique mechanism for viral infectivity.

In the literature, the FD of class one fusion proteins undergoes conformational changes, often becoming helical, to penetrate the host cell membrane and mediate fusion. For example, the HIV FD adopts an amphipathic helix upon membrane insertion, contributing to the destabilization of the target host cell membrane such that fusion can occur (29–31,37,94–96). The influenza FD must also undertake a similar structure transition to mediate fusion, but instead adopts a helical hairpin structure (32–36,97,98). While the LASV FD undergoes a pH-dependent conformational change from the pre- (Fig. 2 *A*; Table S2) to postfusion (Fig. 2 *B*; Table S3) state, our results suggest that the FP remains solvent exposed while the FL has helical content and is the primary membrane-interacting component (Fig. 2 *D*). More specifically, a helix is adopted for residues R^{282}-L^{290} within the FL (Fig. 2 *C*), which is juxtaposed to a properly formed disulfide bond between C^{279} and C^{290} (Figs. S5 and S6) that is important for the tertiary structure and initial interaction of the FD with the host cell membrane. We speculate that this disulfide bond serves to stabilize the helix in the membrane and increase the energy required to remove it (46,50,99). This agrees with the EBOV FD, which mediates fusion through a hydrophobic fist that is clamped by a disulfide bond (28,38,39,43,100,101). However, while the EBOV FD inserts itself deeply within the membrane, our PRE experiments indicate that the LASV FD associates with the membrane more superficially, just beneath the lipid headgroups (Figs. 2 *D* and S8; Table S4). Our experiments probing molecule dynamics agreed that the LASV FD became more restricted once fusion occurred, especially the FL (Figs. 2 *E* and S9; Table S5). This aligns more closely with the SARS-CoV-2 FD, which also has both an FP and FL in tandem. In the SARS-CoV-2 FD, the FP adopts a boomerang structure, akin to the influenza FD, and becomes more restricted after association below the lipid headgroups. In contrast, the FL remains exposed to the environment and dynamic, serving as a mechanical stabilizer for the FP and interacting with extracellular factors to increase fusogenicity (44,47–49). Thus, we suggest that the FP and FL of the LASV FD have opposite roles as their counterparts in the SARS-CoV-2 FD. The FL forms a boat-like structure that is capped by a disulfide bond and facilitates the initial interaction with the membrane, while the FP, especially residues D^{268}-T^{274}, functions as its mechanical stabilizer and is like a sail for the boat (Fig. 7).

During our structural investigation, we uncovered multiple peaks with different ^{1}H-^{15}N shifts, but identical Cα, Cβ, and CO shifts in both the pre- (Fig. S11) and postfusion (Fig. S12) states. One plausible explanation would be that a single residue adopted multiple conformations on a slow enough timescale, i.e., milliseconds to seconds, that could be captured via solution NMR spectroscopy. For example, recent studies have revealed alternative conformations undergone by the influenza FD on a submillisecond scale and suggested structural plasticity (32,33,102–111). A three-model system was thus developed to explain these distinct conformers as each had a different tertiary structure that uniquely influenced fusion pore formation. This aligns with a new theory that has recently emerged as structural intermediates have been described for various class one and two fusion proteins, which are believed to properly orient their FDs for membrane remodeling (112–115). We postulate that the LASV FD also follows a multisystem model to carry out fusion with up to four different conformations (Fig. 3 *A* and *B*); however, a much wider array of structural transitions may be quickly sampled and, thus, not captured during our NMR experiments. This likely affects the side chains more than the backbone as the predicted dihedral angles were relatively similar between the different conformers in both the pre- (Table S7) and postfusion (Table S8) states. Our ^{19}F experiments, which are

particularly sensitive to environmental changes, support this notion as additional conformers were observed in both the pre- (Fig. S14 *A*) and postfusion (Fig. S14 *B*) states when the side chain of F^{262} was specifically labeled at the *para* position of the aromatic. In the prefusion state, roughly half of the LASV FD transiently samples multiple conformations with residues distributed throughout both the FP (9 residues, 60%) and FL (7 residues, 40%) having different conformers (Fig. 3 *C*; Table S6). The LASV FD also contains a highly conserved proline residue at position 275 within its linker region, which in principle can undergo *cis-trans* isomerization. The resolution of the current data does not allow us to conclusively identify a *cis-trans* population, the presence of proline could contribute to the conformational heterogeneity observed in the spectra. This large sampling of alternative conformations by the LASV FD in the prefusion state explains the small, but uniform, reduction in the overall signal intensity upon Gd-DTPA titration (Fig. S7). A similar phenomenon has been observed in intrinsically disordered proteins (IDPs) where the Gd-DTPA probe does not form a complex with the IDP but rather becomes homogeneously distributed around the IDP (116–118). IDPs are notoriously extremely flexible, often functioning without a well-defined structure until folding, which is often binding induced. In the prefusion state, we contend that the LASV FD behaves similarly to an IDP and slowly samples alternative conformations to extensively interact with various environmental factors. Once the right conditions are sensed, the FD is triggered to transition into the postfusion state so that fusion occurs at the appropriate location.

Interestingly, in the postfusion state, the FP (eight residues, 53%) continued to effectively adopt alternative conformations, whereas the FL (four residues, 24%) was largely limited to a single conformer (Fig. 3 *D*; Table S6). We theorize that the FP continued to sample multiple conformations and remained dynamic in the postfusion state as it was still solvent exposed (Figs. 2 *D* and S8; Table S4). This is in alignment with our notion that adopting multiple conformations likely allows for more interactions with the environment to increase fusion. In contrast, the FL undergoes binding-induced folding and is “locked” into a single conformer, which provides a rationalization for why only the peripheral residues continued to transiently populate alternate conformers in the postfusion state. The formation of the helix within the FL thus potentially functions as the rate-limiting step for the multisystem model of the LASV FD, akin to the influenza FD. It is important to note that one of these residues within the FL, E^{289}, is part of a salt bridge shown to significantly influence the structure and function of the LASV FD (42). In influenza, it has been postulated that the conformational change between the different structures is driven by changes in the protonation states of the acidic side chains at low pH and is an early rate-limiting step (111,119–122). We believe that protonation of E^{289} results in a similar occurrence and helps to drive the transition of the FL into its single, postfusion conformer. This is in agreement with literature where the protonation of E^{289} at a low pH results in the formation of a new bonding network that prompts the adoption of the fusogenic, helical conformation (42). Notably, this includes the bonding network with R^{282}, the salt bridge counterpart to E^{289}, which is also important for the structure and function of the LASV FD, but to a lesser extent than E^{289}. Taken together, this indicates that the LASV FD follows a multisystem model to mediate fusion, potentially exploiting its conformational plasticity to have optimal fusion under varying physiological conditions (Fig. 7).

One of the most important, but often overlooked, environmental factors in membrane fusion is the lipid composition of the target host cell membrane. A key feature of LASV FD-initiated fusion is its preference for the lysosomal compartment, where the fusogenic, helical conformation is only adopted at a low pH of 4.0 (24–27,42,123,124). The predominant lipids of the lysosomal membrane are phosphatidylcholine, PE, BMP, sphingomyelin, and cholesterol at a molar ratio of 50:20:15:10:5, respectively (92). Additionally, there are trace amounts of the anionic lipids phosphatidylinositol and PS at roughly 2–3% and 1–2%, respectively, but totaling no more than 5%. BMP is an anionic lipid that is specific to the lysosome and is the most abundant anionic lipid present in this organelle. Moreover, PE is unusual in that it does not carry a charge but has an inverted cone shape similar to that argued to exist within BMP, which is known to induce negative curvature in lipid membranes and elicit fusion more readily (88–90,93). Therefore, this led us to theorize that the LASV FD interacts with specific lipids, namely anionic lipids, to influence fusion. We show that increased concentrations of POPG, a rudimentary anionic lipid, resulted in a progressively higher affinity of the FD for the lipid bilayer (Figs. 4 *A*–*C*). More specifically, the FD existed in its nonfusogenic, random coil form in vesicles containing little to no concentrations of POPG, but transitioned into its fusogenic, helical form as the concentration of POPG was increased (Fig. 5 *A*), which correlated to a positive, exponential increase in fusion (Fig. 5 *B*). We postulate that, while a low pH may be necessary for fusion to occur, it in itself is insufficient to trigger a robust interaction between the LASV FD and the target host cell membrane. Collectively, this gives credence to our hypothesis that anionic lipids impart a specific function within LASV FD-initiated fusion.

A preference for anionic lipids has been linked to positively charged residues in several class one fusion proteins, including HIV and SARS-CoV-2 (45,51,125). Given the similarities between the LASV FD and SARS-CoV-2 FD, we decided to investigate if positively charged residues in the LASV FD contributed to the anionic lipid preference. A sequence alignment of the LASV FD alongside other arenaviruses revealed three positively charged residues: K^{272}, R^{282}, and K^{291} (Fig. 1). K^{272} is located in the FP and not

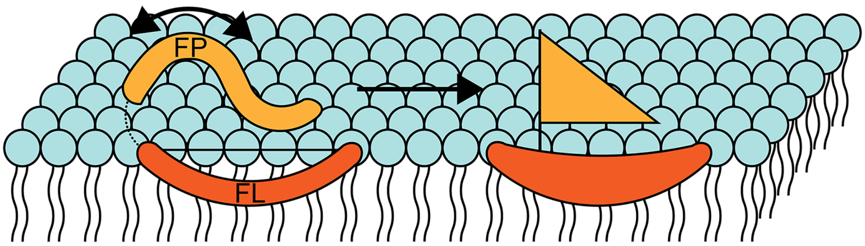

FIGURE 7 Structural model of the LASV FD associated with the host cell membrane. The Fl (*orange*, C^{279}-N^{295}) adopts a helix (R^{282}-L^{290}) that is capped by a disulfide bond (*black line*, C^{279} and C^{292}) and associates with the membrane in a shallow manner, almost like a boat. The FP (*yellow*, G^{260}-T^{274}) remains solvent exposed, especially the residues (D^{268}-T^{274}) closest to the linker region (*dotted line*, P^{275}-Y^{278}), and adopts multiple conformations (*double-ended arrow*) to engage with the additional extracellular factors, acting as a sail for the boat.

conserved, whereas R^{282} and K^{291} were both located in the FL and conserved to different degrees. Single mutants were created such that K^{272} and K^{291} were converted to a chemically inert alanine (i.e., K^{272}A and K^{291}A), while R^{282} was not mutated as its necessity for the structure and function of the LASV FD was previously established. We hypothesized that K^{291} would be most likely to have an ionic interaction with the anionic lipids given its position in the lipid headgroup, as demonstrated via our NMR experiments (Figs. 2 *D* and S8), and degree of conservation. In turn, we believed that a lack of charge at this position would impede the necessary ionic interaction and result in decreased function and/or structural perturbations. On the other hand, mutation of K^{272} would have no significant impact on the function or structure of the LASV FD as it remains solvent exposed throughout the fusion process. Unexpectedly, we observed no significant structural change in either mutant when compared with WT (Fig. 5 *C*). Subsequently, the binding affinity (Fig. S15, *A* and *B*) and fusogenicity (Fig. 5 *D*) of both mutants were similar to WT. These findings thus suggest that the LASV FD does not rely on ionic interactions to mediate its interaction with anionic lipids but rather exploits lipid-specific properties to facilitate fusion.

Anionic lipids, particularly BMP, have been implicated to have a positive and specific influence on fusion for several other viruses that employ the endocytic pathway, such as influenza, SARS-CoV-2, and vesicular stomatitis virus (45,89–91,126). For example, studies on the entry of flaviviruses, which include class two fusion proteins such as dengue virus and Zika virus, indicate that their FDs preferentially interact with anionic lipids, including BMP, to promote viral fusion (127–129). We hypothesized that a similar preference might influence LASV FD-initiated fusion, especially since the lysosomal compartment has a high composition of BMP and the FD is specifically influenced by the property of a lipid itself. We also postulated that POPS and POPE could impart a particular interaction as well given that they are also known to influence fusion. Surprisingly, we found that the FD had a significantly higher affinity for BMP over the other anionic lipids (Fig. S16, *A*–*D*). Additionally, helical content (Fig. 6 *A*) and, thus, fusion (Fig. 6 *B*) were only noted in BMP and were similar to that seen in POPG. MD simulations reinforced these observations, demonstrating that the LASV FD remains helical and membrane associated in a lipid environment with POPG (Figs 6 *C*, S18 *A*, and S19 *A*; Video S1) and BMP (Figs. 6 *D*, S18 *B*, and S19 *B*; Video S2), but becomes more disordered in the presence of POPS (Figs 6 *E*, S18 *C*, and S19 *C*; Video S3). It could be claimed that this observation was due to a difference in the tail saturation between POPS and BMP and the headgroup charge between POPE and BMP. However, both of these properties should have been captured when POPS and POPE were in tandem, yet we observed no significant difference in the structure or function of the LASV FD when POPS and POPE were in combination or alone. Even when we introduced vesicles with the same lipid headgroups of PG, PS, or phosphatidic acid, but different tail saturations (i.e., single unsaturation [POXX, 16:0–18:1] vs. double unsaturation [DOXX, 18:1]), we saw no significant difference in fusion (Fig. S17). A previous study on the complete LASV GPC also indicated a specific effect on fusion mediated by BMP and not POPS or DOPS (88). Based on our findings, a possible explanation is that LASV FD-initiated fusion has a specificity for a particular type of anionic headgroup moiety. In the literature, it has been shown that the headgroup of BMP is the only mammalian glycerophospholipid to have a distinctive *sn*-1;*sn*-1′ arrangement, as opposed to the traditional *sn*-3:*sn*-1′ orientation, that confers increased stability and is found in PG (130,131). This unique configuration has been found to directly influence fusion by the SARS-CoV-2 FD and likely explains why we observed some fusion in POPA/DOPA, which has partial similarity to BMP and PG (45). Nonetheless, the preference of the SARS-CoV-2 FD for BMP is a result of both the unique bonding network for the headgroup moiety and decreased lipid packing caused by the increased unsaturation of the tails, which is not true for the LASV FD (50). Although membrane curvature and leaflet asymmetry are also known to play important roles in membrane fusion, such features are difficult to reproduce in our systems and thus were not directly probed here. Put together, these findings enforce the idea that LASV FD-initiated fusion is optimized for the unique composition of the lysosomal membrane, with a distinct preference for the unique headgroup moiety found in BMP.

In summary, our study provides new insights into the structural plasticity of the LASV FD and its role in viral entry. We have illustrated that conformational changes occur from the pre-to postfusion state with the FP and FL having distinct structural and function roles. Here, the ability of the FD to sample multiple conformations may be a key evolutionary advantage that allows it to fine-tune its fusion process in response to different lipid environments and initiate fusion at the appropriate time. Additionally, the low pH of the lysosomal compartment alone is necessary, but insufficient to trigger fusion with the target host cell membrane and requires anionic lipids. Positively charged residues within the FD do not impart a specific, ionic interaction with anionic lipids. Instead, the LASV FD takes advantage of the membrane disorder created by BMP to preferentially interact with the membrane and initiate fusion. Overall, we have provided a deeper understanding of the molecular details governing the structure and function of the LASV FD. These findings highlight the critical role of both the conformational flexibility of the FD and anionic lipids, chiefly BMP, in fusion and suggest that disrupting either of these interactions could serve as a novel antiviral strategy. Further research is warranted to explore the implications of these alternative conformations and the role of lipids in a broader context of related arenaviruses, potentially providing insights into viral evolution and therapeutic targets.

DATA AND CODE AVAILABILITY

The associated NMR chemical shifts have been deposited in the BMRB under the accession numbers 53085 and 53084 for the pre- and postfusion states, correspondingly.

ACKNOWLEDGMENTS

We thank all lab members for their assistance in editing this manuscript.

This work was supported by the National Science Foundation (CHE-2238139 and MCB-2111728 to J.L. and W.I., respectively) and the National Institutes of Health Shared Instrumentation Grant Program (1S10OD 030350-01).

AUTHOR CONTRIBUTIONS

H.N.P. and J.L. designed the experiments. H.N.P. performed the experiments and was supported by K.P. and Q.M.M. H.N.P. analyzed the data. S.S. and W.I. performed and analyzed the MD simulations. H.N.P. and J.L. prepared the manuscript.

DECLARATION OF INTERESTS

The authors declare no conflict of interest.

SUPPORTING MATERIAL

Supporting material can be found online at https://doi.org/10.1016/j.bpj.2026.01.049.

REFERENCES

1. Richmond, J. K., and D. J. Baglole. 2003. Lassa fever: epidemiology, clinical features, and social consequences. *Br Med J.* 327:1271–1275.
2. Woyessa, A. B., L. Maximore, …, N. Mahmoud. 2019. Lesson learned from the investigation and response of Lassa fever outbreak, Margibi County, Liberia, 2018: case report. *BMC Infect. Dis.* 19:610.
3. Safronetz, D., J. E. Lopez, …, H. Feldmann. 2010. Detection of Lassa virus, Mali. *Emerg. Infect. Dis.* 16:1123–1126.
4. Frame, J. D., J. M. Baldwin, …, J. M. Troup. 1970. Lassa Fever, a New Virus Disease of Man from West Africa: I. Clinical Description and Pathological Findings. *Am. J. Trop. Med. Hyg.* 19:670–676.
5. Yaro, C. A., E. Kogi, …, J. P. Oni. 2021. Infection pattern, case fatality rate and spread of Lassa virus in Nigeria. *BMC Infect. Dis.* 21:149.
6. McCormick, J. B. 1986. Clinical, epidemiologic, and therapeutic aspects of Lassa fever. *Med. Microbiol. Immunol.* 175:153–155.
7. McCormick, J. B., P. A. Webb, …, E. S. Smith. 1987. A Prospective Study of the Epidemiology and Ecology of Lassa Fever. *J. Infect. Dis.* 155:437–444.
8. Okogbenin, S., J. Okoeguale, …, E. Ogbaini-Emovon. 2019. Retrospective Cohort Study of Lassa Fever in Pregnancy, Southern Nigeria. *Emerg. Infect. Dis.* 25:1494–1500.
9. Organization, W. H. 2022. Lassa Fever - United Kingdom of Great Britain and Northern Ireland. https://www.who.int/emergencies/disease-outbreak-news/item/lassa-fever-united-kingdom-of-great-britain-and-northern-ireland.
10. Lecompte, E., E. Fichet-Calvet, …, J. Ter Meulen. 2006. Mastomys natalensis and Lassa Fever, West Africa. *Emerg. Infect. Dis.* 12:1971–1974.
11. Olayemi, A., D. Cadar, …, E. Fichet-Calvet. 2016. New Hosts of The Lassa Virus. *Sci. Rep.* 6:25280.
12. Mire, C. E., R. W. Cross, …, T. W. Geisbert. 2017. Human-monoclonal-antibody therapy protects nonhuman primates against advanced Lassa fever. *Nat. Med.* 23:1146–1149.
13. Eberhardt, K. A., J. Mischlinger, …, M. Ramharter. 2019. Ribavirin for the treatment of Lassa fever: A systematic review and meta-analysis. *Int. J. Infect. Dis.* 87:15–20.
14. World Health Organization. 2021. Prioritizing Diseases for Research and Development in Emergency Contexts. https://www.who.int/activities/prioritizing-diseases-for-research-and-development-in-emergency-contexts.
15. McCormick, J. B., I. J. King, …, R. Belmont-Williams. 1986. Lassa fever. Effective therapy with ribavirin. *N. Engl. J. Med.* 314:20–26.
16. Katz, M., J. Weinstein, …, R. Diskin. 2022. Structure and receptor recognition by the Lassa virus spike complex. *Nature.* 603:174–179.
17. Hastie, K. M., M. A. Zandonatti, …, E. O. Saphire. 2017. Structural basis for antibody-mediated neutralization of Lassa virus. *Science.* 356:923–928.
18. Pennington, H. N., and J. Lee. 2022. Lassa virus glycoprotein complex review: Insights into its unique fusion machinery. *Biosci. Rep.* 42
19. Oppliger, J., G. Torriani, …, S. Kunz. 2016. Lassa Virus Cell Entry via Dystroglycan Involves an Unusual Pathway of Macropinocytosis. *J. Virol.* 90:6412–6429.
20. Guo, J., Y. Wan, …, W. Wang. 2024. Identification of residues in Lassa virus glycoprotein 1 involved in receptor switch. *Virol. Sin.* 39:600–608.
21. Pasqual, G., J. M. Rojek, …, S. Kunz. 2011. Old World Arenaviruses Enter the Host Cell via the Multivesicular Body and Depend on the Endosomal Sorting Complex Required for Transport. *PLoS Pathog.* 7:e1002232.
22. Cao, W., M. D. Henry, …, M. B. Oldstone. 1998. Identification of α-Dystroglycan as a Receptor for Lymphocytic Choriomeningitis Virus and Lassa Fever Virus. *Science.* 282:2079–2081.

23. Acciani, M., J. T. Alston, …, M. A. Brindley. 2017. Mutational Analysis of Lassa Virus Glycoprotein Highlights Regions Required for Alpha-Dystroglycan Utilization. *J. Virol.* 91:e00574-17.
24. Bulow, U., R. Govindan, and J. B. Munro. 2020. Acidic pH Triggers Lipid Mixing Mediated by Lassa Virus GP. *Viruses.* 12:716.
25. Cohen-Dvashi, H., N. Cohen, …, R. Diskin. 2015. Molecular Mechanism for LAMP1 Recognition by Lassa Virus. *J. Virol.* 89:7584–7592.
26. Hulseberg, C. E., L. Fénéant, …, J. M. White. 2018. Lamp1 Increases the Efficiency of Lassa Virus Infection by Promoting Fusion in Less Acidic Endosomal Compartments. *mBio.* 9:1–14.
27. Cohen-Dvashi, H., H. Israeli, …, R. Diskin. 2016. Role of LAMP1 Binding and pH Sensing by the Spike Complex of Lassa Virus. *J. Virol.* 90:10329–10338.
28. Li, S., Z. Sun, …, J. T. Huiskonen. 2016. Acidic pH-Induced Conformations and LAMP1 Binding of the Lassa Virus Glycoprotein Spike. *PLoS Pathog.* 12:e1005418.
29. Dimitrov, A. S., S. S. Rawat, …, R. Blumenthal. 2003. Role of the fusion peptide and membrane-proximal domain in HIV-1 envelope glycoprotein-mediated membrane fusion. *Biochemistry.* 42:14150–14158.
30. Li, Y., and L. K. Tamm. 2007. Structure and plasticity of the human immunodeficiency virus gp41 fusion domain in lipid micelles and bilayers. *Biophys. J.* 93:876–885.
31. Lai, A. L., A. E. Moorthy, …, L. K. Tamm. 2012. Fusion activity of HIV gp41 fusion domain is related to its secondary structure and depth of membrane insertion in a cholesterol-dependent fashion. *J. Mol. Biol.* 418:3–15.
32. Chang, D. K., S. F. Cheng, …, Y. T. Liu. 2008. Membrane interaction and structure of the transmembrane domain of influenza hemagglutinin and its fusion peptide complex. *BMC Biol.* 6:2–12.
33. Han, X., J. H. Bushweller, …, L. K. Tamm. 2001. Membrane structure and fusion-triggering conformational change of the fusion domain from influenza hemagglutinin. *Nat. Struct. Biol.* 8:715–720.
34. Smrt, S. T., A. W. Draney, and J. L. Lorieau. 2015. The influenza hemagglutinin fusion domain is an amphipathic helical hairpin that functions by inducing membrane curvature. *J. Biol. Chem.* 290:228–238.
35. Gray, C., S. A. Tatulian, …, L. K. Tamm. 1996. Effect of the N-terminal glycine on the secondary structure, orientation, and interaction of the influenza hemagglutinin fusion peptide with lipid bilayers. *Biophys. J.* 70:2275–2286.
36. Gething, M. J., R. W. Doms, …, J. White. 1986. Studies on the mechanism of membrane fusion: site-specific mutagenesis of the hemagglutinin of influenza virus. *J. Cell Biol.* 102:11–23.
37. Qiang, W., Y. Sun, and D. P. Weliky. 2009. A strong correlation between fusogenicity and membrane insertion depth of the HIV fusion peptide. *Proc. Natl. Acad. Sci. USA.* 106:15314–15319.
38. Gregory, S. M., P. Larsson, …, L. K. Tamm. 2014. Ebolavirus entry requires a compact hydrophobic fist at the tip of the fusion loop. *J. Virol.* 88:6636–6649.
39. Gregory, S. M., E. Harada, …, L. K. Tamm. 2011. Structure and function of the complete internal fusion loop from Ebolavirus glycoprotein 2. *Proc. Natl. Acad. Sci. USA.* 108:11211–11216.
40. Glushakova, S. E., I. S. Lukashevich, and L. A. Baratova. 1990. Prediction of arenavirus fusion peptides on the basis of computer analysis of envelope protein sequences. *FEBS Lett.* 269:145–147.
41. Willard, K. A., J. T. Alston, …, M. A. Brindley. 2018. Identification of Residues in Lassa Virus Glycoprotein Subunit 2 That Are Critical for Protein Function. *Pathogens.* 8:1–13.
42. Pennington, H. N., D. Birtles, …, J. Lee. 2024. A Salt Bridge and Disulfide Bond within the Lassa Virus Fusion Domain Are Required for the Initiation of Membrane Fusion. *ACS Omega.* 9:4920–4930.
43. Lee, J., S. M. Gregory, …, L. K. Tamm. 2016. The Roles of Histidines and Charged Residues as Potential Triggers of a Conformational Change in the Fusion Loop of Ebola Virus Glycoprotein. *PLoS One.* 11:e0152527.
44. Birtles, D., and J. Lee. 2021. Identifying Distinct Structural Features of the SARS-CoV-2 Spike Protein Fusion Domain Essential for Membrane Interaction. *Biochemistry.* 60:2978–2986.
45. Birtles, D., W. Abbas, and J. Lee. 2024. Bis(Monoacylglycero)Phosphate Promotes Membrane Fusion Facilitated by the SARS-CoV-2 Fusion Domain. *J. Phys. Chem. B.* 128:2675–2683.
46. Birtles, D., A. E. Oh, and J. Lee. 2022. Exploring the pH dependence of the SARS-CoV-2 complete fusion domain and the role of its unique structural features. *Protein Sci.* 31:e4390.
47. Lai, A. L., J. K. Millet, …, G. R. Whittaker. 2017. The SARS-CoV Fusion Peptide Forms an Extended Bipartite Fusion Platform that Perturbs Membrane Order in a Calcium-Dependent Manner. *J. Mol. Biol.* 429:3875–3892.
48. Madu, I. G., S. L. Roth, …, G. R. Whittaker. 2009. Characterization of a Highly Conserved Domain within the Severe Acute Respiratory Syndrome Coronavirus Spike Protein S2 Domain with Characteristics of a Viral Fusion Peptide. *J. Virol.* 83:7411–7421.
49. Santamaria, A., K. C. Batchu, …, N. R. Zaccai. 2022. Strikingly Different Roles of SARS-CoV-2 Fusion Peptides Uncovered by Neutron Scattering. *J. Am. Chem. Soc.* 144:2968–2979.
50. Schaefer, S. L., H. Jung, and G. Hummer. 2021. Binding of SARS-CoV-2 Fusion Peptide to Host Endosome and Plasma Membrane. *J. Phys. Chem. B.* 125:7732–7741.
51. Birtles, D., L. Guiyab, …, J. Lee. 2024. Positive residues of the SARS-CoV-2 fusion domain are key contributors to the initiation of membrane fusion. *J. Biol. Chem.* 300:107564.
52. Pennington, H. N., and J. Lee. 2025. Acidic bicelles are a suitable membrane mimic for structural studies of the Lassa Virus Fusion Domain. *Biomembranes.* 1867:184428.
53. Studier, F. W. 2005. Protein production by auto-induction in high-density shaking cultures. *Protein Expr. Purif.* 41:207–234.
54. Truhlar, S. M. E., C. F. Cervantes, …, E. A. Komives. 2008. Rapid mass spectrometric analysis of 15N-Leu incorporation fidelity during preparation of specifically labeled NMR samples. *Protein Sci.* 17:1636–1639.
55. Glover, K. J., J. A. Whiles, …, R. R. Vold. 2001. Structural Evaluation of Phospholipid Bicelles for Solution-State Studies of Membrane-Associated Biomolecules. *Biophys. J.* 81:2163–2171.
56. Delaglio, F., S. Grzesiek, …, A. Bax. 1995. NMRPipe: A multidimensional spectral processing system based on UNIX pipes. *J. Biomol. NMR.* 6:277–293.
57. Lee, W., M. Tonelli, and J. L. Markley. 2015. NMRFAM-SPARKY: enhanced software for biomolecular NMR spectroscopy. *Bioinformatics.* 31:1325–1327.
58. Maciejewski, M. W., A. D. Schuyler, …, J. C. Hoch. 2017. NMRbox: A Resource for Biomolecular NMR Computation. *Biophys. J.* 112:1529–1534.
59. Wishart, D. S., and B. D. Sykes. 1994. The 13C chemical-shift index: a simple method for the identification of protein secondary structure using 13C chemical-shift data. *J. Biomol. NMR.* 4:171–180.
60. Wishart, D. S., B. D. Sykes, and F. M. Richards. 1991. Relationship between nuclear magnetic resonance chemical shift and protein secondary structure. *J. Mol. Biol.* 222:311–333.
61. Shen, Y., F. Delaglio, …, A. Bax. 2009. TALOS+: a hybrid method for predicting protein backbone torsion angles from NMR chemical shifts. *J. Biomol. NMR.* 44:213–223.
62. Lee, W., M. Rahimi, …, A. Chiu. 2021. POKY: a software suite for multidimensional NMR and 3D structure calculation of biomolecules. *Bioinformatics.* 37:3041–3042.
63. Miles, A. J., and B. A. Wallace. 2018. CDtoolX, a downloadable software package for processing and analyses of circular dichroism spectroscopic data. *Protein Sci.* 27:1717–1722.
64. Shepherd, N. E., H. N. Hoang, …, D. P. Fairlie. 2005. Single Turn Peptide Alpha Helices with Exceptional Stability in Water. *J. Am. Chem. Soc.* 127:2974–2983.

65. Kwok, S. C., and R. S. Hodges. 2004. Stabilizing and destabilizing clusters in the hydrophobic core of long two-stranded alpha-helical coiled-coils. *J. Biol. Chem.* 279:21576–21588.

66. Scheuermann, T. H., and C. A. Brautigam. 2015. High-precision, automated integration of multiple isothermal titration calorimetric thermograms: new features of NITPIC. *Methods.* 76:87–98.

67. Zhao, H., G. Piszczek, and P. Schuck. 2015. SEDPHAT–a platform for global ITC analysis and global multi-method analysis of molecular interactions. *Methods.* 76:137–148.

68. Brautigam, C. A. 2015. Calculations and Publication-Quality Illustrations for Analytical Ultracentrifugation Data. *Methods Enzymol.* 562:109–133.

69. Villalaín, J. 2022. Interaction of Lassa virus fusion and membrane proximal peptides with late endosomal membranes. *Biochim. Biophys. Acta Biomembr.* 1864:184031.

70. Promsri, S., G. M. Ullmann, and S. Hannongbua. 2012. Molecular dynamics simulation of HIV-1 fusion domain-membrane complexes: Insight into the N-terminal gp41 fusion mechanism. *Biophys. Chem.* 170:9–16.

71. Lee, J., D. S. Patel, …, W. Im. 2018. CHARMM-GUI Membrane Builder for Complex Biological Membrane Simulations with Glycolipids and Lipoglycans. *J. Chem. Theory Comput.* 15:775–786.

72. Jo, S., T. Kim, …, W. Im. 2008. CHARMM-GUI: a web-based graphical user interface for CHARMM. *J. Comput. Chem.* 29:1859–1865.

73. Wu, E. L., X. Cheng, …, W. Im. 2014. CHARMM-GUI*Membrane Builder*toward realistic biological membrane simulations. *J. Comput. Chem.* 35:1997–2004.

74. Brooks, B. R., C. L. Brooks, 3rd, …, D. M. York. 2009. CHARMM: the biomolecular simulation program. *J. Comput. Chem.* 30:1545–1614.

75. Huang, J., S. Rauscher, …, A. D. MacKerell. 2017. CHARMM36m: an improved force field for folded and intrinsically disordered proteins. *Nat. Methods.* 14:71–73.

76. Jorgensen, W. L., J. Chandrasekhar, …, M. L. Klein. 1983. Comparison of simple potential functions for simulating liquid water. *J. Chem. Phys.* 79:926–935.

77. Eastman, P., R. Galvelis, …, T. E. Markland. 2024. OpenMM 8: Molecular Dynamics Simulation with Machine Learning Potentials. *J. Phys. Chem. B.* 128:109–116.

78. Jo, S., T. Kim, and W. Im. 2007. Automated builder and database of protein/membrane complexes for molecular dynamics simulations. *PLoS One.* 2:e880.

79. Lee, J., X. Cheng, …, J. B. Klauda. 2016. CHARMM-GUI Input Generator for NAMD, GROMACS, AMBER, OpenMM, and CHARMM/OpenMM Simulations Using the CHARMM36 Additive Force Field. *J. Chem. Theory Comput.* 12:405–413.

80. Michaud-Agrawal, N., E. J. Denning, …, O. Beckstein. 2011. MDAnalysis: a toolkit for the analysis of molecular dynamics simulations. *J. Comput. Chem.* 32:2319–2327.

81. Gowers, R., M. Linke, …, O. Beckstein. 2019. MDAnalysis: A Python Package for the Rapid Analysis of Molecular Dynamics Simulations. *In* Proceedings of the Python in Science Conference. SciPy, pp. 98–105.

82. Humphrey, W., A. Dalke, and K. Schulten. 1996. VMD: visual molecular dynamics. *J. Mol. Graph.* 14:33.

83. Wirmer, J., W. Peti, and H. Schwalbe. 2006. Motional properties of unfolded ubiquitin: a model for a random coil protein. *J. Biomol. NMR.* 35:175–186.

84. Ramelot, T. A., R. Tejero, and G. T. Montelione. 2023. Representing structures of the multiple conformational states of proteins. *Curr. Opin. Struct. Biol.* 83:102703.

85. Fox, R. O., P. A. Evans, and C. M. Dobson. 1986. Multiple conformations of a protein demonstrated by magnetization transfer NMR spectroscopy. *Nature.* 320:192–194.

86. Khago, D., I. J. Fucci, and R. A. Byrd. 2020. The Role of Conformational Dynamics in the Recognition and Regulation of Ubiquitination. *Molecules.* 25:5933.

87. Yu, J.-X., R. R. Hallac, …, R. P. Mason. 2013. New frontiers and developing applications in 19F NMR. *Prog. Nucl. Magn. Reson. Spectrosc.* 70:25–49.

88. Markosyan, R. M., M. Marin, …, G. B. Melikyan. 2021. The late endosome-resident lipid bis(monoacylglycero)phosphate is a cofactor for Lassa virus fusion. *PLoS Pathog.* 17:e1009488.

89. Luquain-Costaz, C., M. Rabia, …, I. Delton. 2020. Bis(monoacylglycero)phosphate, an important actor in the host endocytic machinery hijacked by SARS-CoV-2 and related viruses. *Biochimie.* 179:247–256.

90. Roth, S. L., and G. R. Whittaker. 2011. Promotion of vesicular stomatitis virus fusion by the endosome-specific phospholipid bis(monoacylglycero)phosphate (BMP). *FEBS Lett.* 585:865–869.

91. Mannsverk, S., A. M. Villamil Giraldo, and P. M. Kasson. 2022. Influenza Virus Membrane Fusion Is Promoted by the Endosome-Resident Phospholipid Bis(monoacylglycero)phosphate. *J. Phys. Chem. B.* 126:10445–10451.

92. van Meer, G., D. R. Voelker, and G. W. Feigenson. 2008. Membrane lipids: where they are and how they behave. *Nat. Rev. Mol. Cell Biol.* 9:112–124.

93. Pohl, E. E., and O. Jovanovic. 2019. The Role of Phosphatidylethanolamine Adducts in Modification of the Activity of Membrane Proteins under Oxidative Stress. *Molecules.* 24:4545.

94. Lai, A. L., and J. H. Freed. 2014. HIV gp41 fusion peptide increases membrane ordering in a cholesterol-dependent fashion. *Biophys. J.* 106:172–181.

95. Chan, D. C., D. Fass, …, P. S. Kim. 1997. Core structure of gp41 from the HIV envelope glycoprotein. *Cell.* 89:263–273.

96. Freed, E. O., D. J. Myers, and R. Risser. 1990. Characterization of the fusion domain of the human immunodeficiency virus type 1 envelope glycoprotein gp41. *Proc. Natl. Acad. Sci. USA.* 87:4650–4654.

97. Lüneberg, J., I. Martin, …, A. Herrmann. 1995. Structure and topology of the influenza virus fusion peptide in lipid bilayers. *J. Biol. Chem.* 270:27606–27614.

98. Das, D. K., R. Govindan, …, J. B. Munro. 2018. Direct Visualization of the Conformational Dynamics of Single Influenza Hemagglutinin Trimers. *Cell.* 174:926–937.e12.

99. Shen, H., and Z. Wu. 2022. Effect of Disulfide Bridge on the Binding of SARS-CoV-2 Fusion Peptide to Cell Membrane: A Coarse-Grained Study. *ACS Omega.* 7:36762–36775.

100. Kirchdoerfer, R. N., D. M. Abelson, …, E. O. Saphire. 2015. Assembly of the Ebola Virus Nucleoprotein from a Chaperoned VP35 Complex. *Cell Rep.* 12:140–149.

101. Das, D. K., U. Bulow, …, J. B. Munro. 2020. Conformational changes in the Ebola virus membrane fusion machine induced by pH, Ca2+, and receptor binding. *PLoS Biol.* 18:e3000626.

102. Lai, A. L., H. Park, …, L. K. Tamm. 2006. Fusion peptide of influenza hemagglutinin requires a fixed angle boomerang structure for activity. *J. Biol. Chem.* 281:5760–5770.

103. Lai, A. L., and L. K. Tamm. 2007. Locking the kink in the influenza hemagglutinin fusion domain structure. *J. Biol. Chem.* 282:23946–23956.

104. Légaré, S., and P. Lagüe. 2012. The influenza fusion peptide adopts a flexible flat V conformation in membranes. *Biophys. J.* 102:2270–2278.

105. Vaccaro, L., K. J. Cross, …, F. Fraternali. 2005. Plasticity of influenza haemagglutinin fusion peptides and their interaction with lipid bilayers. *Biophys. J.* 88:25–36.

106. Huang, Q., C. L. Chen, and A. Herrmann. 2004. Bilayer conformation of fusion peptide of influenza virus hemagglutinin: a molecular dynamics simulation study. *Biophys. J.* 87:14–22.

107. Bodner, M. L., C. M. Gabrys, …, D. P. Weliky. 2008. 13C-13C and (15)N-(13)C correlation spectroscopy of membrane-associated and

uniformly labeled human immunodeficiency virus and influenza fusion peptides: amino acid-type assignments and evidence for multiple conformations. *J. Chem. Phys.* 128:052319.

108. Sun, Y., and D. P. Weliky. 2009. 13C-13C correlation spectroscopy of membrane-associated influenza virus fusion peptide strongly supports a helix-turn-helix motif and two turn conformations. *J. Am. Chem. Soc.* 131:13228–13229.

109. Jang, H., N. Michaud-Agrawal, …, T. B. Woolf. 2008. How to lose a kink and gain a helix: pH independent conformational changes of the fusion domains from influenza hemagglutinin in heterogeneous lipid bilayers. *Proteins.* 72:299–312.

110. Dubovskii, P. V., H. Li, …, K. Akasaka. 2000. Structure of an analog of fusion peptide from hemagglutinin. *Protein Sci.* 9:786–798.

111. Lorieau, J. L., J. M. Louis, …, A. Bax. 2012. pH-triggered, activated-state conformations of the influenza hemagglutinin fusion peptide revealed by NMR. *Proc. Natl. Acad. Sci. USA.* 109:19994–19999.

112. Hover, S., F. W. Charlton, …, J. Fontana. 2023. Organisation of the orthobunyavirus tripodal spike and the structural changes induced by low pH and K(+) during entry. *Nat. Commun.* 14:5885.

113. Mangala Prasad, V., J. S. Blijleven, …, K. K. Lee. 2022. Visualization of conformational changes and membrane remodeling leading to genome delivery by viral class-II fusion machinery. *Nat. Commun.* 13:4772.

114. Chen, C. L., T. Klose, …, R. J. Kuhn. 2022. Cryo-EM structures of alphavirus conformational intermediates in low pH-triggered prefusion states. *Proc. Natl. Acad. Sci. USA.* 119:e2114119119.

115. White, J. M., A. E. Ward, …, L. K. Tamm. 2023. Viral Membrane Fusion: A Dance Between Proteins and Lipids. *Annu. Rev. Virol.* 10:139–161.

116. Kooshapur, H., C. D. Schwieters, and N. Tjandra. 2018. Conformational Ensemble of Disordered Proteins Probed by Solvent Paramagnetic Relaxation Enhancement (sPRE). *Angew Chem Int Ed.* 57:13519–13522.

117. Kosol, S., S. Contreras-Martos, …, P. Tompa. 2013. Structural characterization of intrinsically disordered proteins by NMR spectroscopy. *Molecules.* 18:10802–10828.

118. Grossauer, J., S. Kosol, …, K. Zangger. 2010. The peptide hormone ghrelin binds to membrane-mimetics via its octanoyl chain and an adjacent phenylalanine. *Bioorg. Med. Chem.* 18:5483–5488.

119. Lorieau, J. L., J. M. Louis, and A. Bax. 2010. The complete influenza hemagglutinin fusion domain adopts a tight helical hairpin arrangement at the lipid:water interface. *Proc. Natl. Acad. Sci. USA.* 107:11341–11346.

120. Benton, D. J., S. J. Gamblin, …, J. J. Skehel. 2020. Structural transitions in influenza haemagglutinin at membrane fusion pH. *Nature.* 583:150–153.

121. Benhaim, M. A., V. Mangala Prasad, …, K. K. Lee. 2020. Structural monitoring of a transient intermediate in the hemagglutinin fusion machinery on influenza virions. *Sci. Adv.* 6:eaaz8822.

122. Li, Z., T. Li, …, T. Ivanovic. 2022. Hemagglutinin Stability Determines Influenza A Virus Susceptibility to a Broad-Spectrum Fusion Inhibitor Arbidol. *ACS Infect. Dis.* 8:1543–1552.

123. Klewitz, C., H. D. Klenk, and J. ter Meulen. 2007. Amino acids from both N-terminal hydrophobic regions of the Lassa virus envelope glycoprotein GP-2 are critical for pH-dependent membrane fusion and infectivity. *J. Gen. Virol.* 88:2320–2328.

124. Cosset, F. L., P. Marianneau, …, B. Bartosch. 2009. Characterization of Lassa virus cell entry and neutralization with Lassa virus pseudoparticles. *J. Virol.* 83:3228–3237.

125. Zaitseva, E., E. Zaitsev, …, L. V. Chernomordik. 2017. Fusion Stage of HIV-1 Entry Depends on Virus-Induced Cell Surface Exposure of Phosphatidylserine. *Cell Host Microbe.* 22:99–110.e7.

126. Matos, P. M., M. Marin, …, G. B. Melikyan. 2013. Anionic Lipids Are Required for Vesicular Stomatitis Virus G Protein-mediated Single Particle Fusion with Supported Lipid Bilayers. *J. Biol. Chem.* 288:12416–12425.

127. Zaitseva, E., S. T. Yang, …, L. V. Chernomordik. 2010. Dengue Virus Ensures Its Fusion in Late Endosomes Using Compartment-Specific Lipids. *PLoS Pathog.* 6:e1001131.

128. Modis, Y., S. Ogata, …, S. C. Harrison. 2004. Structure of the dengue virus envelope protein after membrane fusion. *Nature.* 427:313–319.

129. Klein, D. E., J. L. Choi, and S. C. Harrison. 2013. Structure of a dengue virus envelope protein late-stage fusion intermediate. *J. Virol.* 87:2287–2293.

130. Dickey, A., and R. Faller. 2008. Examining the contributions of lipid shape and headgroup charge on bilayer behavior. *Biophys. J.* 95:2636–2646.

131. Hayakawa, T., Y. Hirano, …, T. Kobayashi. 2006. Differential membrane packing of stereoisomers of bis(monoacylglycero) phosphate. *Biochemistry.* 45:9198–9209.

Biophysical Journal, Volume *125*

Supplemental information

The Lassa virus fusion domain has structural plasticity and exploits bis(monoacylglycero)phosphate for fusion

Hallie N. Pennington, Kiruthika Prahadeesh, Quinn M. Mulvihill, Sungjai Shin, Wonpil Im, and Jinwoo Lee

SUPPORTING INFORMATION

THE LASSA VIRUS FUSION DOMAIN HAS STRUCTURAL PLASTICITY AND EXPLOITS BIS(MONOACYLGLYCERO)PHOSPHATE FOR FUSION

HALLIE N. PENNINGTON[1], KIRUTHIKA PRAHADEESH[1], QUINN M. MULVIHILL[1], SUNGJAI SHIN[1], WONPIL IM[2], AND JINWOO LEE[1]*

[1]Department of Chemistry and Biochemistry, College of Computer, Mathematics, and Natural Science, University of Maryland, College Park, MD, 20740, USA

[2]Department of Biological Sciences, College of Arts and Sciences, Lehigh University, Bethlehem, PA, 18015, USA

*Correspondence: Jinwoo Lee (jinwoo@umd.edu)

Figures	Supporting Information
S1	Structural organization of LASV Glycoprotein complex (GPC) and its viral entry pathway via endocytosis
S2	Structures of purchased lipids
S3	Temperature titration of the LASV FD in the pre- and post-fusion states via solution NMR spectroscopy
S4	Chemical shift indexing of the LASV FD vs BMRB values
S5	Cβ strips for the pre- and post-fusion states of the LASV FD
S6	Thiol assay in the LASV FD under native and reducing conditions
S7	Gd-DTPA titration of the LASV FD in the pre-fusion state
S8	Gd-DTPA and 16-DSA titration of the LASV FD in the post-fusion state
S9	R_1 and R_2 relaxation rates of the LASV FD in the pre- and post-fusion states
S10	^1H – ^{15}N NOE shows that the FD becomes reduced in flexibility upon fusion
S11	^1H – ^{15}N strips for L^{266} in the pre-fusion state
S12	^1H – ^{15}N strips for L^{266} in the post-fusion state
S13	Membrane depth and dynamics of different L^{266} conformations
S14	^{19}F spectra of $F^{293}W$ in the pre- and post-fusion state
S15	Exemplary isotherms of the lysine mutants
S16	Exemplary isotherms of the LASV FD in different lipids

S17	Fusion of the LASV FD in lipids with different tail saturations
S18	Helical graphs of the LASV FD in POPG, BMP, and POPS
S19	Effect of LASV FD on membrane packing
S20	Contact analysis of LASV FD in different anionic lipid membranes

**Tables**	**Supporting Information**
S1	Lipid compositions of the membrane systems generated for MD simulations
S2	TALOS+ statistics for the pre-fusion state
S3	TALOS+ statistics for the post-fusion state
S4	Average relative intensities of LASV FD in different paramagnetic probes
S5	Overall relaxation times of the LASV FD in the pre- and post-fusion state
S6	Populations for residues with multiple conformers in the pre- and post-fusion state
S7	Predicted dihedral angles for different conformers in the pre-fusion state
S8	Predicted dihedral angles for different conformers in the post-fusion state

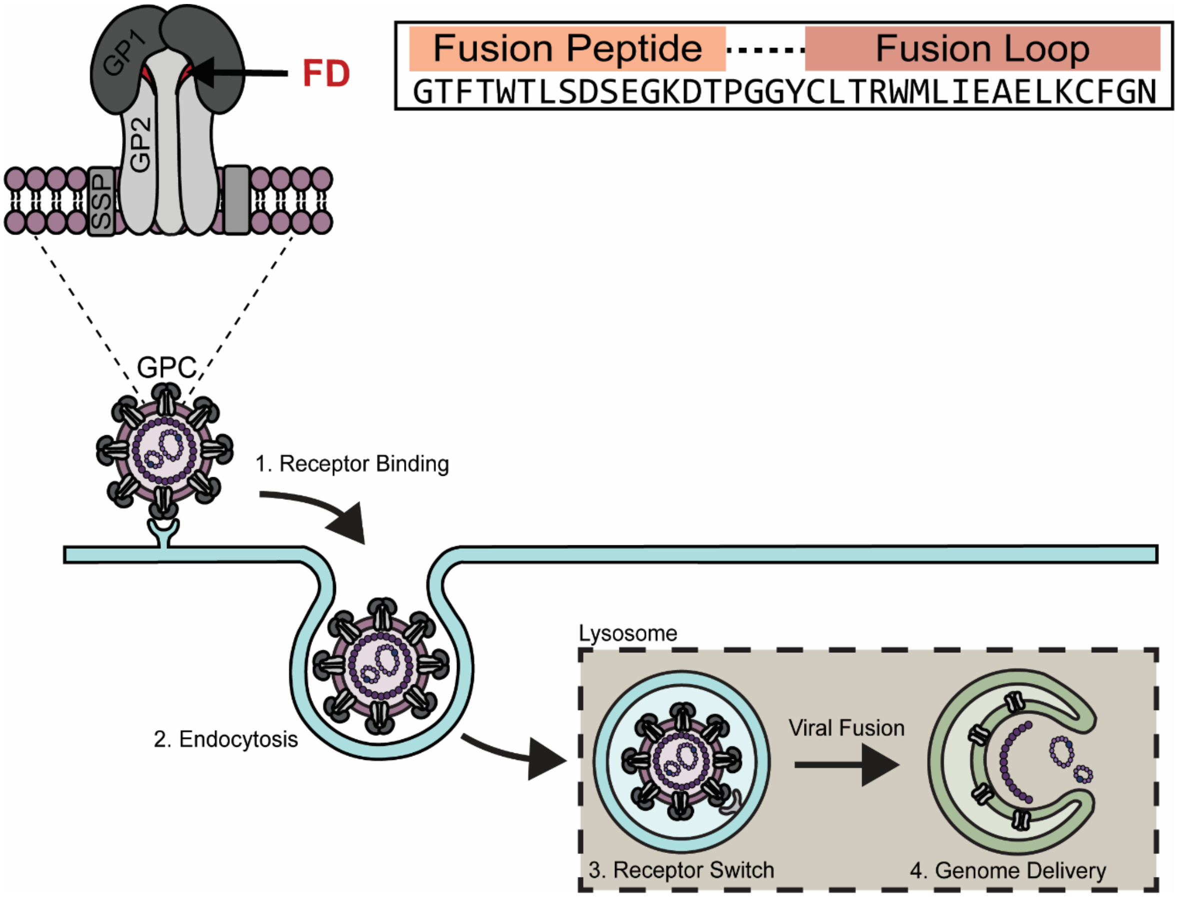

Figure S1: Structural organization of LASV Glycoprotein complex (GPC) and its viral entry pathway via endocytosis. The glycoprotein complex on the Lassa Virus (LASV) surface consists of three components; the receptor binding subunit (GP1), the fusion subunit (GP2), and the stable signal peptide (SSP). Fusion Domain (FD) located at the N-terminus of GP2, is highlighted in red, with its sequence provided to distinguish the fusion peptide (FP) and fusion loop (FL). During the entry, LASV first binds to the host cell receptor through GP1 (Step 1), triggering endocytosis of the virion (Step 2). After internalization, the virion is trafficked to the late endosome/ lysosome where GP1 interact with its secondary receptor, LAMP1 (Step 3). Following GP1 dissociation from GPC, membrane fusion occurs, delivering the viral genome into the host cell cytoplasm (Step 4).

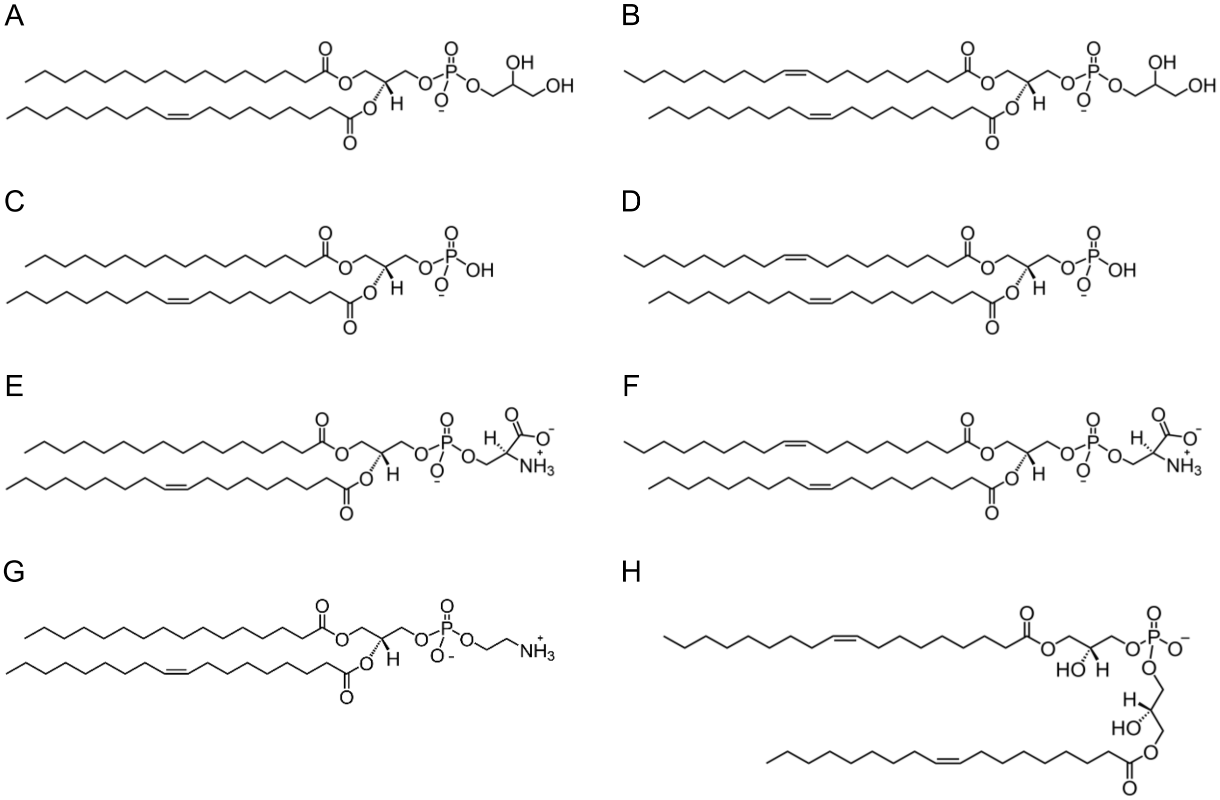

Figure S2. Structure of various lipids employed in this study that were purchased from Avanti Lipids. [A] 16:0–18:1 1-palmitoyl-2-oleoyl-*sn*-glycero-3-phospho-[1′-rac-glycerol] (POPG); [B] 1,2-dioleoyl-*sn*-glycero-3-[phospho-rac-(3-lysyl(1-glycerol))] (DOPG); [C] 1-palmitoyl-2-oleoyl-*sn*-glycero-3-phosphate (POPA); [D] 1,2-dioleoyl-*sn*-glycero-3-phosphate (DOPA); [E] 1-palmitoyl-2-oleoyl-*sn*-glycero-3-phospho-L-serine (POPS); [F] 1,2-dioleoyl-*sn*-glycero-3-phospho-L-serine (DOPS); [G] 1-palmitoyl-2-oleoyl-*sn*-glycero-3-phosphoethanolamine (POPE); and [H] bis(monooleoylglycero)phosphate (S,R Isomer) (BMP). All structures drawn in ChemDraw.

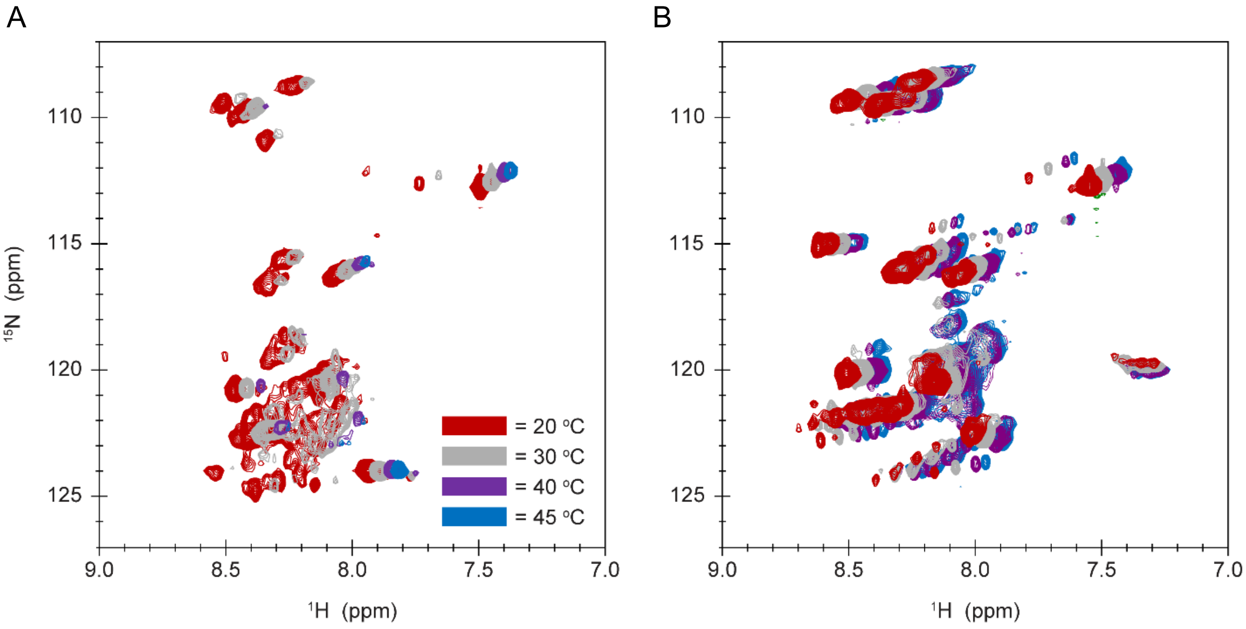

Figure S3. The LASV FD requires a different temperature for the different fusion states to be effectively visualized via NMR spectroscopy. [A,B] An overlay of 1H – 15N HSQC spectra for the [A] pre- and [B] post-fusion state of the LASV FD at 20 ℃ (red), 30 ℃ (grey), 40 ℃ (purple), and 45 ℃ (blue). All measurements were carried out on ~500 μM of the LASV FD in 300 μL of 25 mM Na2HPO4, 100 mM NaCl, pH 7.0 (pre-fusion) or pH 4.0 with acidic bicelles, q = 0.5 (post-fusion).

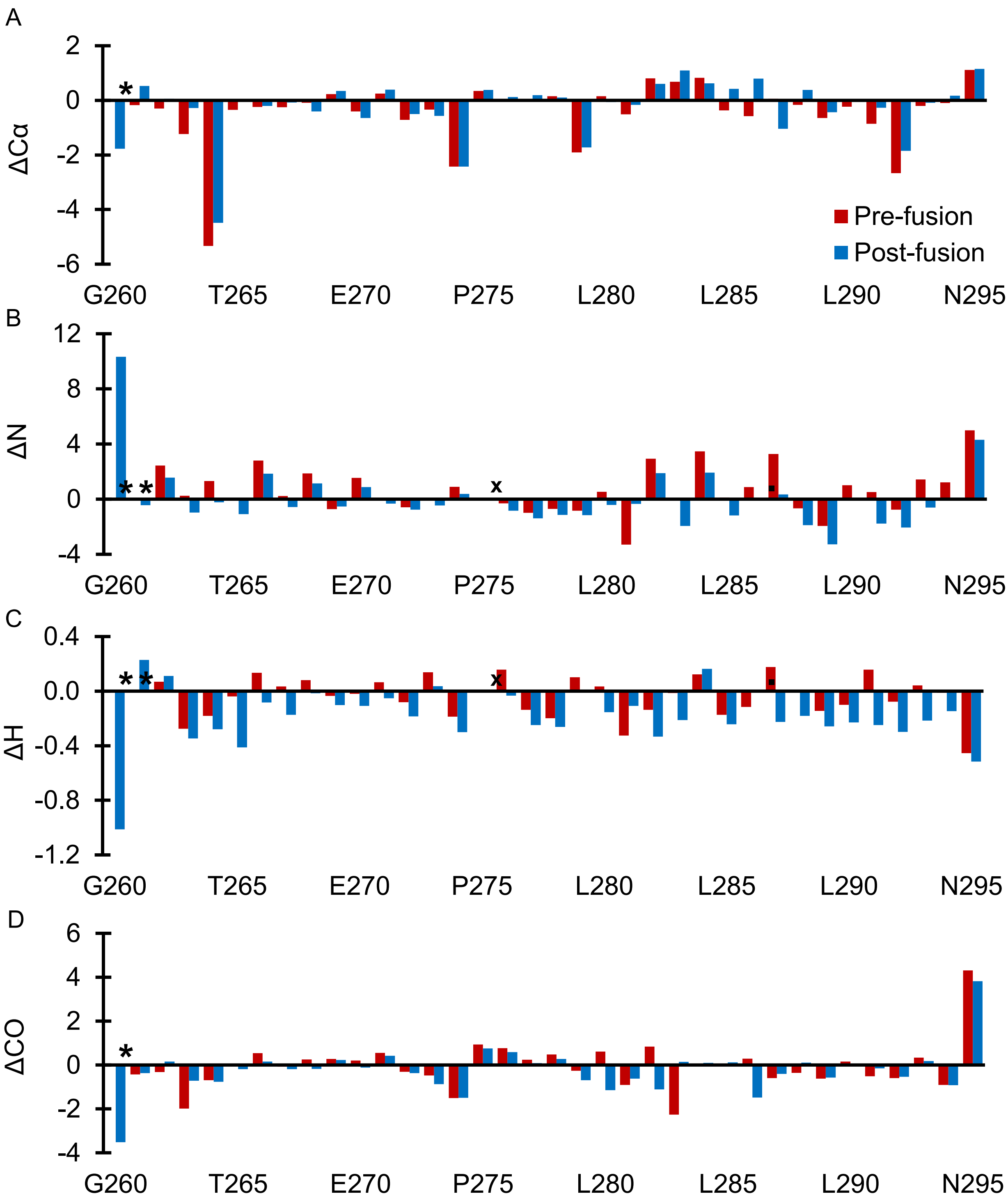

Figure S4. Chemical shift indexing in comparison to backbone atoms does not reveal information regarding the secondary structure of the LASV FD. [A] ΔCα [B] ΔN; [C] ΔH; and [D] ΔCO. All measurements were carried out on ~500 μM of the LASV FD in 300 μL of 25 mM Na2HPO4, 100 mM NaCl, pH 7.0 at 20 ℃ (pre-fusion) or pH 4.0 with acidic bicelles, q = 0.5 at 45 ℃ (post-fusion). Proline residues and other residues that could not be assigned for both states are marked (X), whereas residues that could not be assigned for the pre- or post-fusion state are marked with an asterisk (*) or period (.), accordingly.

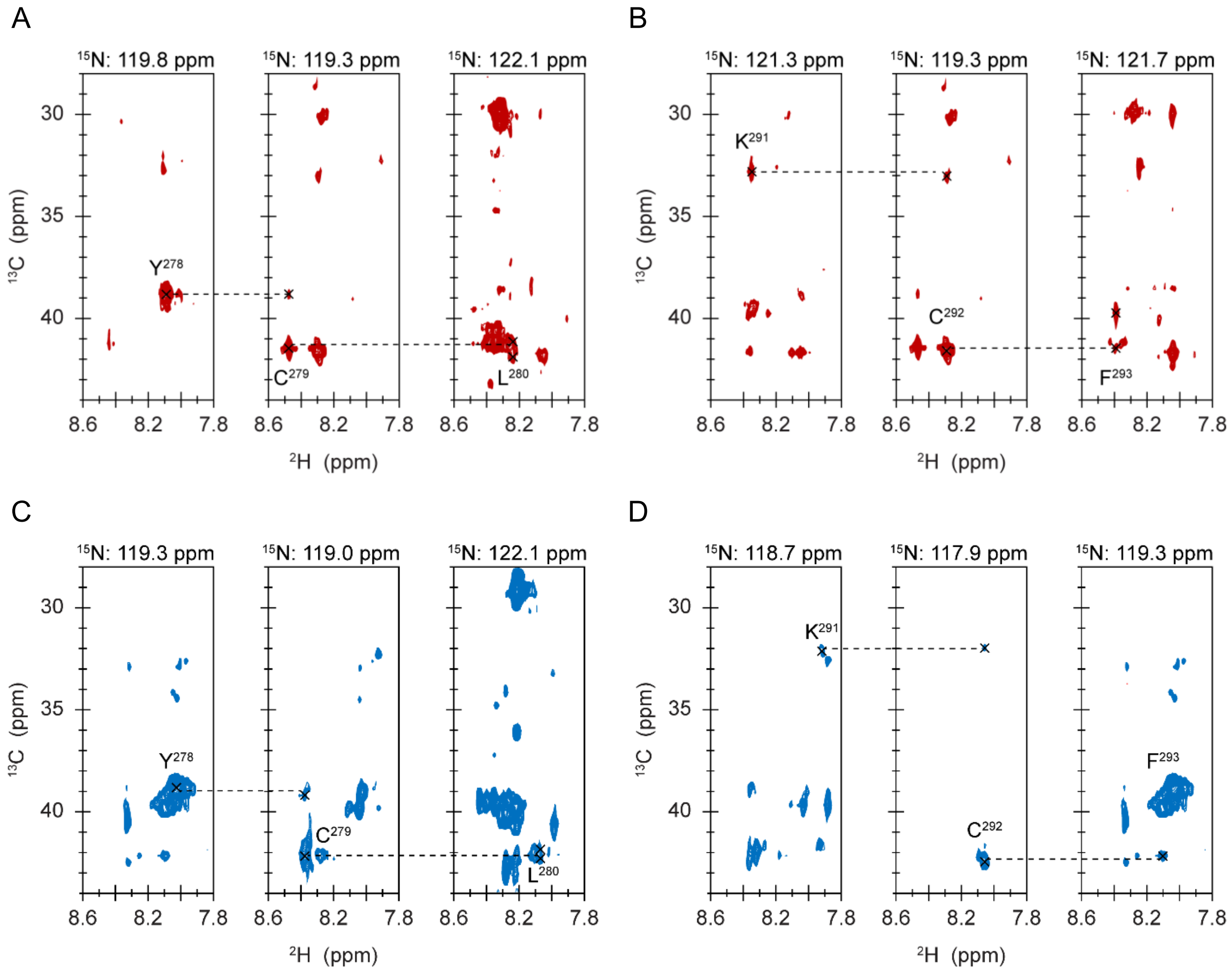

Figure S5. Cβ shifts confirm the presence of an internal disulfide bond within the FL of the LASV FD in both the pre- and post-fusion states. [A] Pre-fusion C279; [B] Pre-fusion C292; [C] Post-fusion C279; and [D] Post-fusion C292 all have 13Cβ chemical shifts greater than 35 ppm, indicative that the cysteine residues were oxidized. All data were acquired from HN(CA)CB experiments with 650 μM of a triple labelled sample (2H/13C/15N) in 25 mM Na2HPO4, 100 mM NaCl, pH 7.0 at 20 ℃ [A, B] or pH 4.0 at 45 ℃ with acidic bicelles, q = 0.5 [C,D].

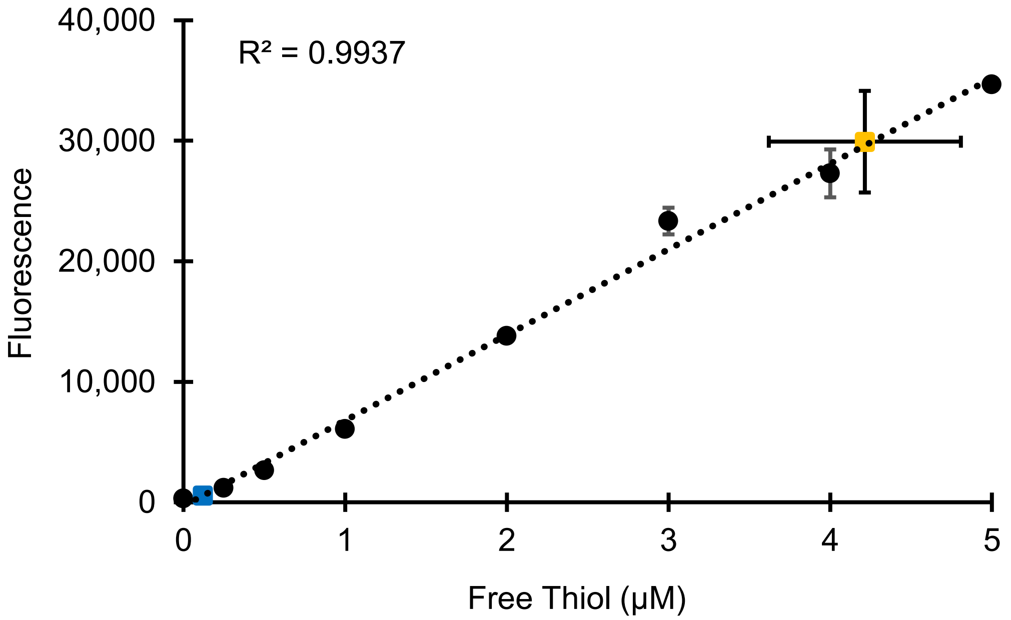

Figure S6. The LASV FD has a properly formed disulfide bond. Under native conditions (blue, square; n = 3), the LASV FD showed that the disulfide bond was properly formed. Upon introduction of 1 mM TCEP to the FD (yellow, square; n = 2), there was a large increase in the concentration of free thiol present, indicative that the disulfide bond was disrupted. Assay was performed according to the manufacturer's protocol with standards in duplicates. Standard curve (black, circle) was generated to determine the unknown thiol concentration. The average fluorescence of 1 mM TCEP was run and subtracted from the average fluorescence of the FD with 1 mM TCEP. Errors were propagated accordingly from the standard deviations.

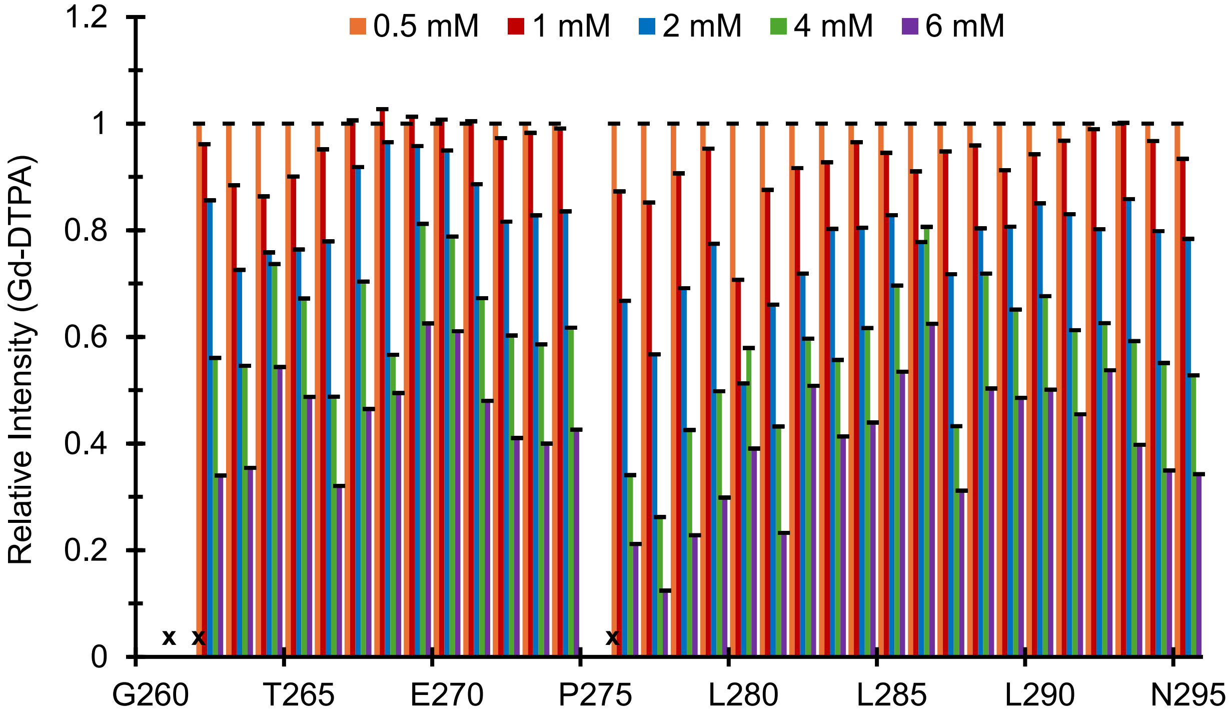

Figure S7. The LASV FD is solvent-exposed in the pre-fusion state. Progressive titration of the water-soluble, paramagnetic agent Gd-DTPA in increments of 0.5 mM (orange), 1 mM (red), 2 mM (blue), 4 mM (green), and 6 mM (blue) into the LASV FD revealed that all residues experience virtually the same amount of quenching. The relative intensity prior to titration is not shown due to increasing signals from the paramagnetic effect. Data normalized to 0.5 mM Gd-DTPA for each titration. Error bars shown are propagated from the signal-to-noise ratio and standard error of the mean (SEM). All measurements were carried out on ~500 μM of the LASV FD in 300 μL of 25 mM Na2HPO4, 100 mM NaCl, pH 7.0 at 20 ℃. Proline residues and other residues that could not be assigned are marked (X).

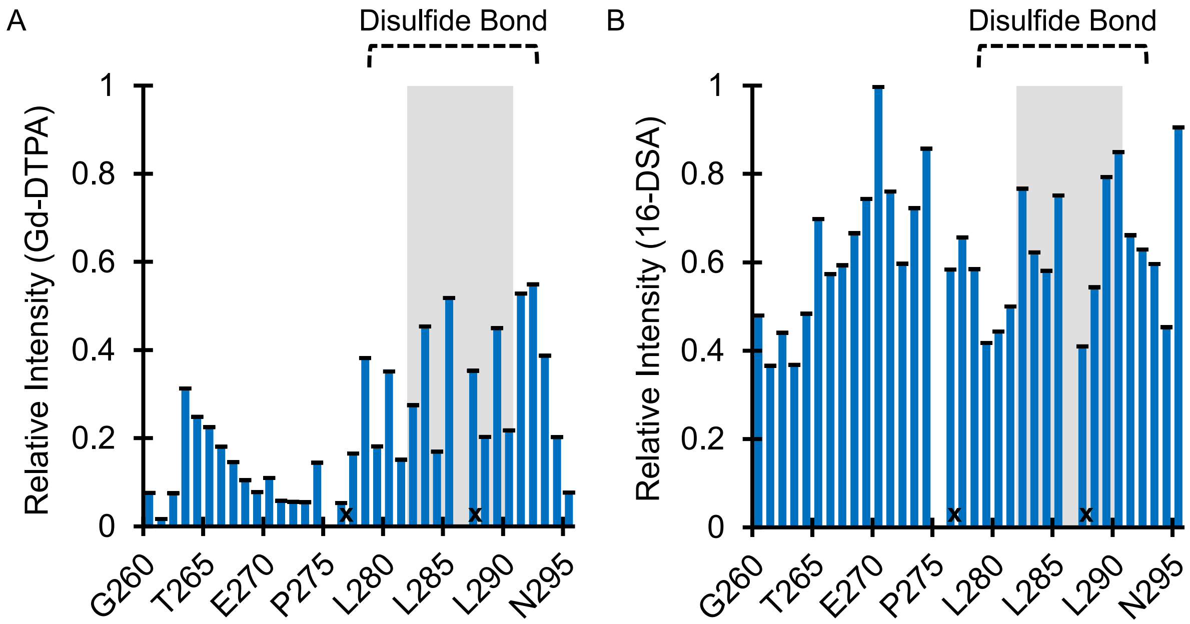

Figure S8. In the post-fusion state, the LASV FD associates with the membrane in a shallow manner via its FL while the FP remains solvent-exposed. [A] Introduction of 6 mM Gd-DTPA into the system containing the LASV FD resulted in significant quenching of the LASV FP, particularly D268 – T274, but not the FL. Titration of 6 mM 16-DSA into the LASV resulted in nearly identical quenching for both the FP and FL. For all experiments, the relative intensity at 0.5 mM of a given probe was subtracted due to increasing signals. All error bars shown are propagated from the signal-to-noise ratio and standard error of the mean (SEM). All measurements were carried out on ~500 μM of the LASV FD in 300 μL of 25 mM Na2HPO4, 100 mM NaCl, pH 4.0 with acidic bicelles, q = 0.5 at 45 ℃. The disulfide bond (C^{279} and C^{292}, dashed line) and helix (R^{282} – L^{290}, transparent grey box) are indicated. Proline residues and other residues that could not be assigned are marked (X).

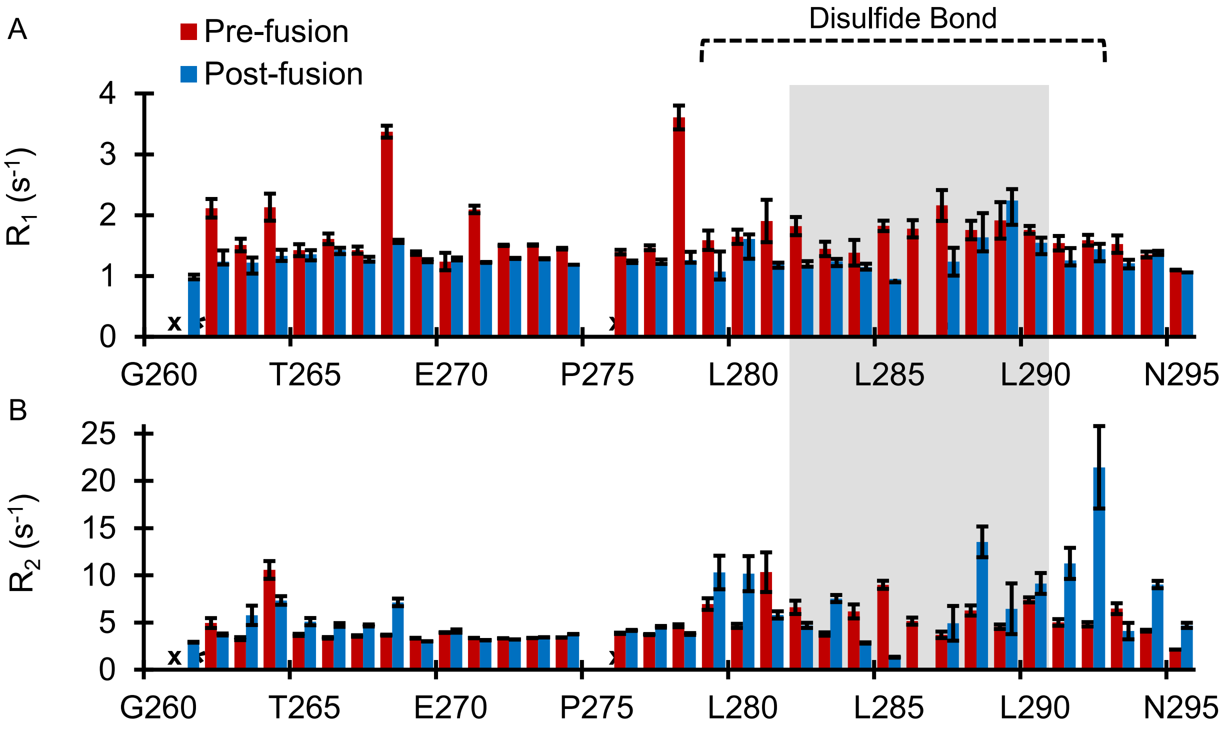

Figure S9. Dynamic properties were slightly altered in several regions within the LASV FD from the pre- (red) to post-fusion (blue) state. [A] Both the FP and FL have similar R_1 relaxation rates in the pre-fusion state that were similar but decreased in the post-fusion state. [B] R_2 relaxation rates for the FP were similar between the pre- and post-fusion states but increased for the FL in the post-fusion state. Error bars shown are propagated from the signal-to-noise ratio. All measurements were carried out on ~500 μM of the LASV FD in 300 μL of 25 mM Na_2HPO_4, 100 mM NaCl, pH 7.0 at 20 ℃ (pre-fusion) or pH 4.0 with acidic bicelles, q = 0.5 at 45 ℃ (post-fusion). The disulfide bond (C^{279} and C^{292}, dashed line) and helix (R^{282} – L^{290}, transparent grey box) are indicated. Proline residues and other residues that could not be assigned for both states are marked (X), whereas residues that could not be assigned for the pre- or post-fusion state are marked with an asterisk (*) or period (.), accordingly.

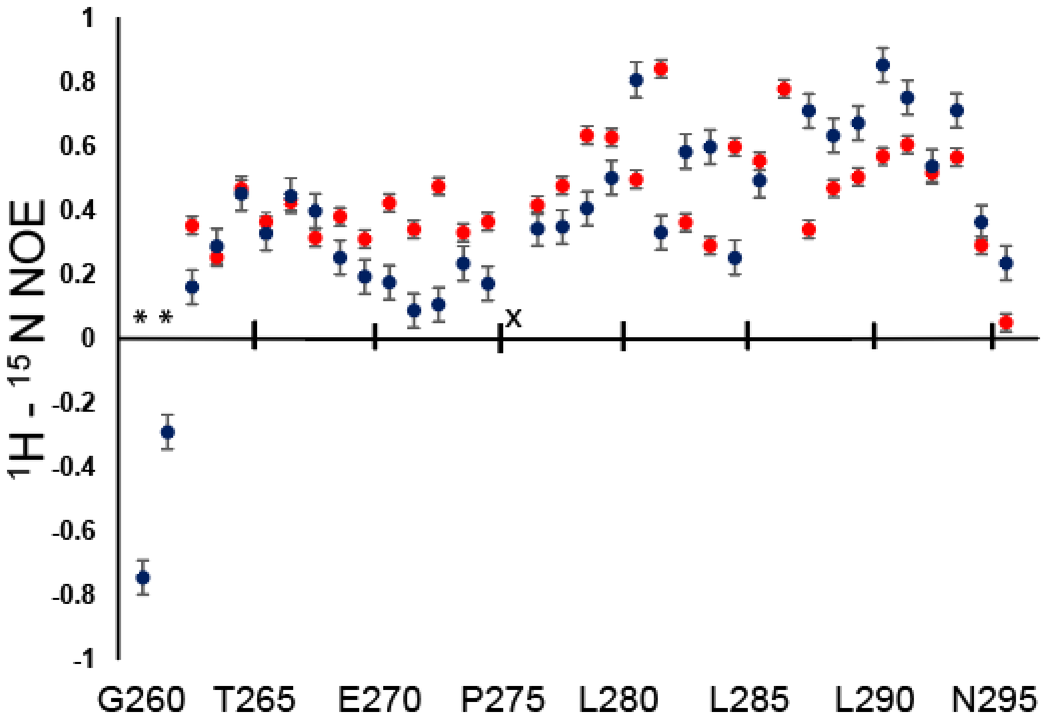

Figure S10. ^{1}H – ^{15}N NOE shows that the FD becomes reduced in flexibility upon fusion (Prefusion-red and postfusion-blue). All measurements were carried out on ~500 μM of the LASV FD in 300 μL of 25 mM Na_2HPO_4, 100 mM NaCl, pH 7.0 at 20 ℃ (pre-fusion) or pH 4.0 with acidic bicelles, q = 0.5 at 45 ℃ (post-fusion). Proline residues and other residues that could not be assigned for both states are marked with an x, whereas residues that could not be assigned for the pre- or post-fusion state are marked with an asterisk (*).

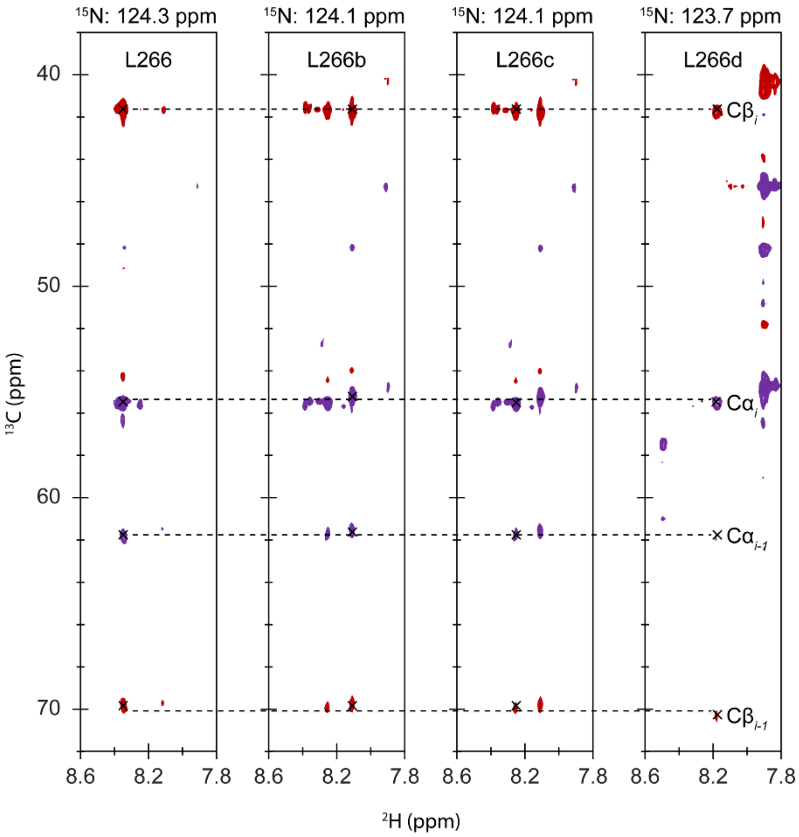

Figure S11. Multiple conformations for L[266] were observed in the pre-fusion states. Four different ^1H – ^{15}N strips had the same Cα (purple) and Cβ (red) shifts for the *i* and *i – 1*, corresponding to L[266] and T[265], accordingly. Data was acquired from HN(CA)CB experiments with 650 µM of a triple labelled sample ($^2H/^{13}C/^{15}N$) in 25 mM Na_2HPO_4, 100 mM NaCl, pH 7.0 at 20 ℃. The most populated conformer (a) is the residue label, whereas the second (b), third (c), and fourth (d) most populated conformers are indicated as such.

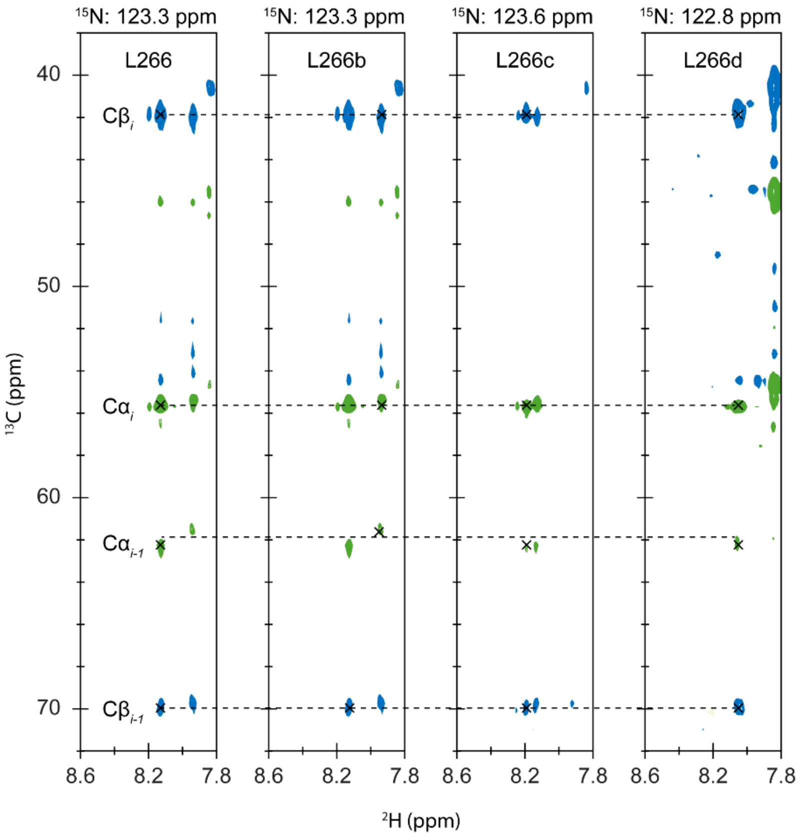

Figure S12. L266 has several conformers in the post-fusion state. Different ^{15}N strips with the same Cα (green) and Cβ (blue) shifts were observed in the post-fusion state for L266. All data were acquired from HN(CA)CB experiments with 650 μM of a triple labelled sample ($^{2}H/^{13}C/^{15}N$) in 25 mM Na_2HPO_4, 100 mM NaCl, pH 4.0 at 45 ℃ with acidic bicelles, q =0.5. The most populated conformer (a) is the residue label, whereas the second (b), third (c), and fourth (d) most populated conformers are indicated as such.

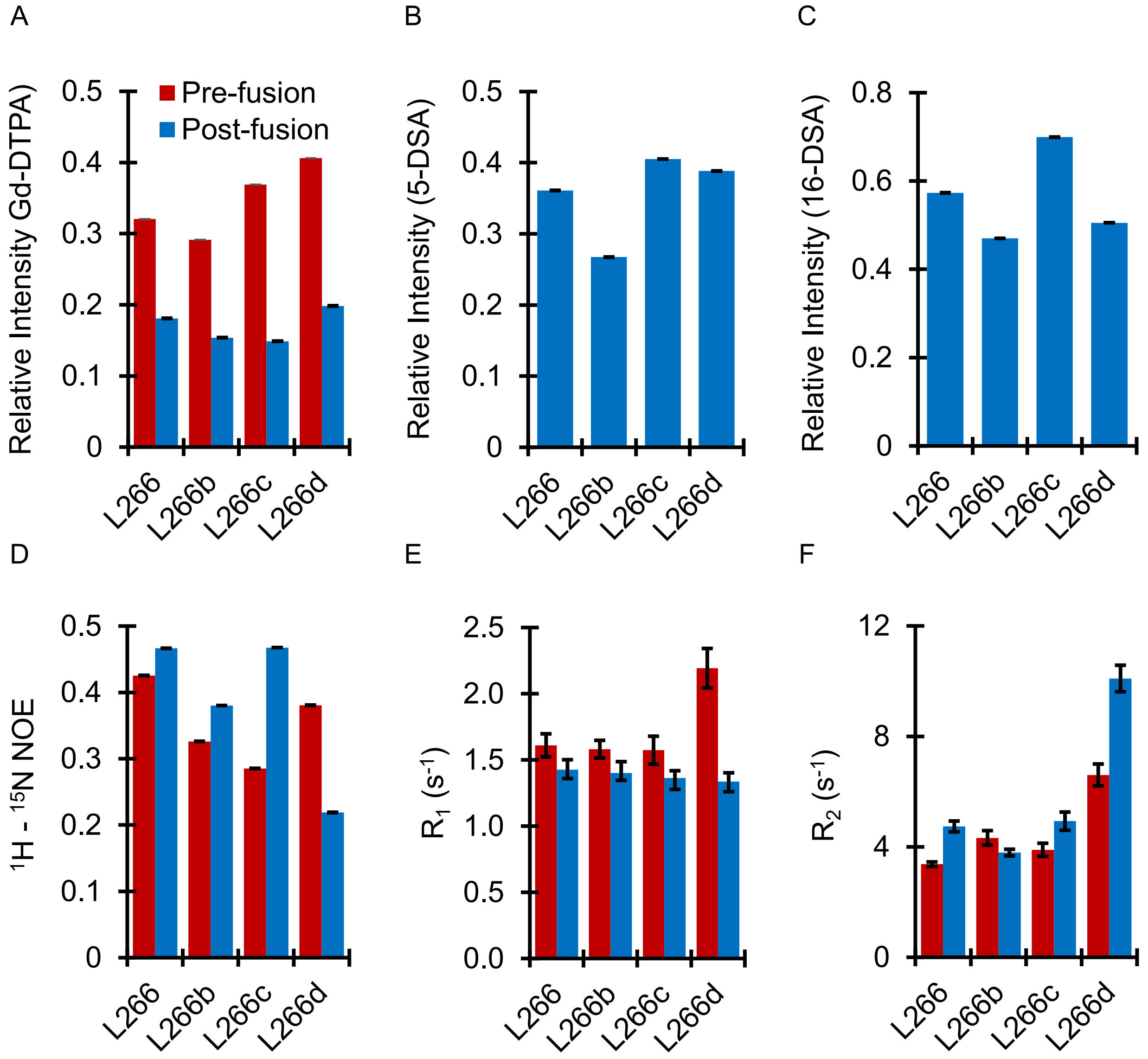

Figure S13. Membrane depth and dynamics of L^{266} are relatively similar between the different conformations. [A-C] Each conformer of L^{266} experiences comparable amounts of quenching in the presence of [A] 6 mM Gd-DTPA, [B] 8 mM 5-DSA, and [C] 6 mM 16-DSA. [D-E] The different L^{266} populations have similar dynamics as revealed by [D] ^{1}H – ^{15}N NOE, [E] R_1 relaxation, and [F] R_2 relaxation experiments. Error bars shown are propagated from the signal-to-noise ratio. All measurements were carried out on ~500 µM of the LASV FD in 300 µL of 25 mM Na_2HPO_4, 100 mM NaCl, pH 7.0 at 20 ℃ (pre-fusion, red) or pH 4.0 with acidic bicelles, q = 0.5 at 45 ℃ (post-fusion, blue). The second (b), third (c), and fourth (d) most populated conformers of a given residue are indicated.

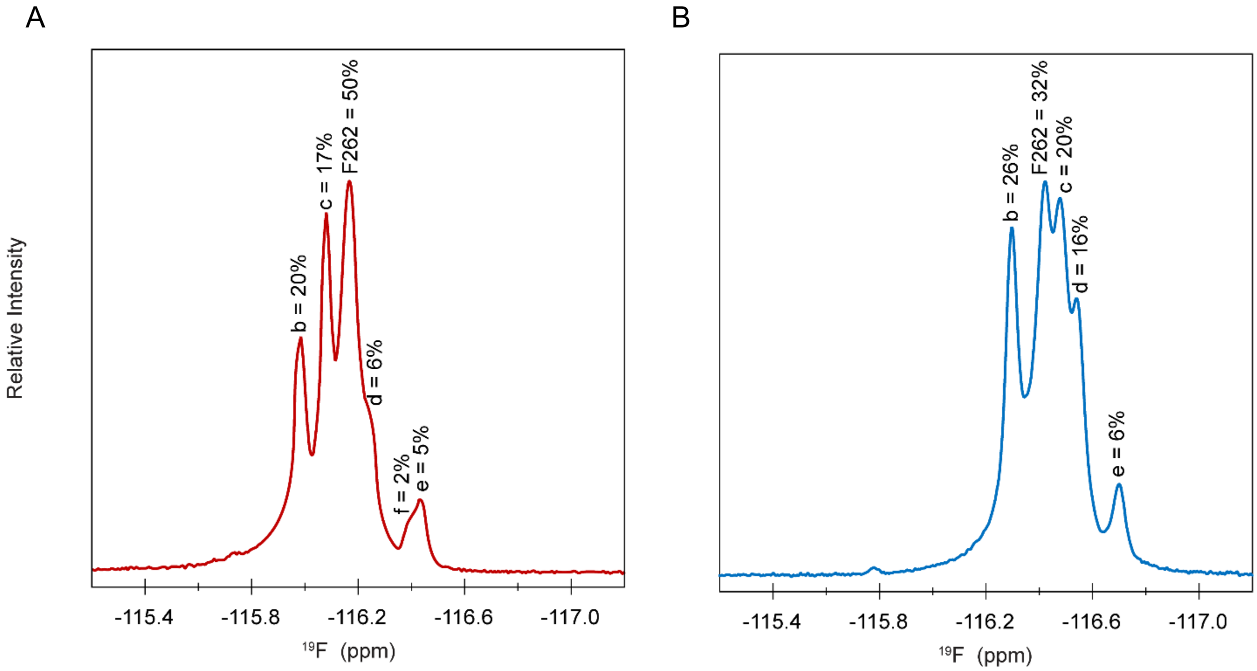

Figure S14. Site specific ^{19}F labeling of F^{262} in the LASV FD revealed additional conformers adopted by the side chain that were not observed in the backbone assignment. [A,B] Each F^{262} conformer is occupied for different percentages of time in the [A] pre- and [B] post-fusion state. All measurements were carried out on ~1,000 μM of $F^{293}W$ in 300 μL of 25 mM Na_2HPO_4, 100 mM NaCl, pH 7.0 at 20 ℃ (pre-fusion) or pH 4.0 with acidic bicelles, q = 0.5 at 45 ℃ (post-fusion). The most populated conformer (a) is the residue label, whereas the second (b), third (c), fourth (d), fifth (e), and sixth (f) most populated conformers are indicated as such.

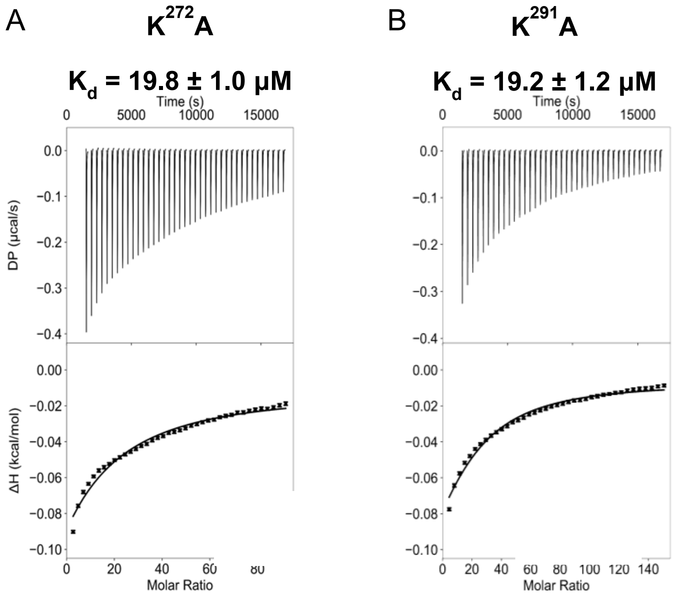

Figure S15. Mutation of the charged lysine residue located within the FP (K^{272}) or FL (K^{291}) to a chemically inert alanine residue had no impact on the binding affinity of the LASV FD. [A] K^{272}A and [B] K^{291}A mutant FD. Both ITC experiments were conducted in 10 mM NaOAc, 100 mM NaCl, pH 4.0 with 65:35 POPC:POPG vesicles titrated into the protein. Dissociation constants (K_d) are displayed above the respective isotherm.

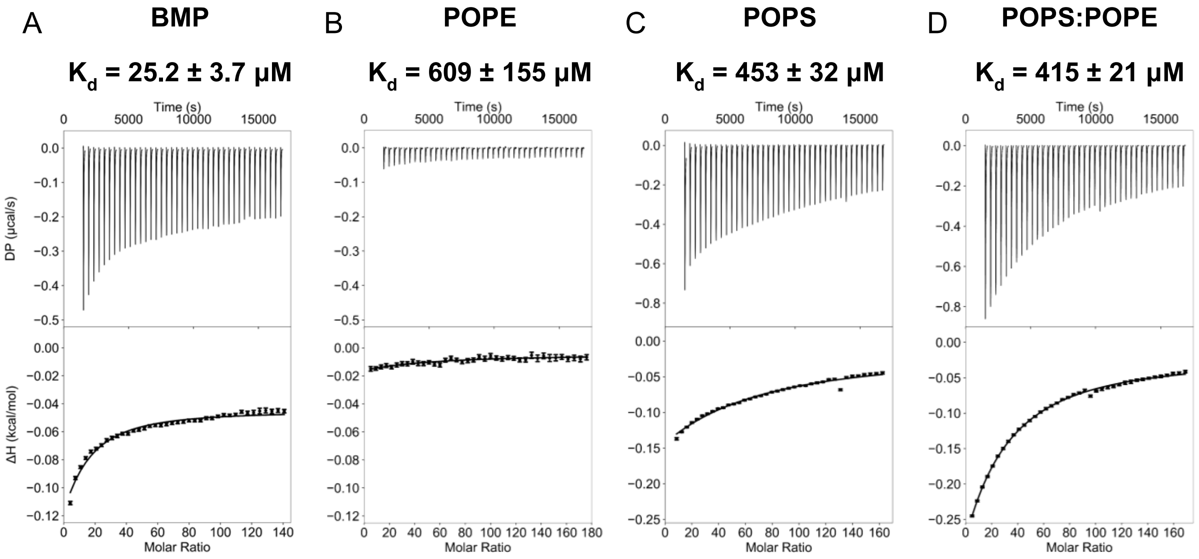

Figure S16. The LASV FD has the highest binding affinity for vesicles comprised of BMP. [A] 65:35 POPC:BMP; [B] 65:35 POPC:POPE; [C] 65:35 POPC:POPS; and [D] 65:17.5:17.5 POPC:POPS:POPE were titrated into the protein. All ITC experiments were conducted in 10 mM NaOAc, 100 mM NaCl, pH 4.0 with vesicles titrated into the protein. Dissociation constants (K_d) are displayed above the respective isotherm.

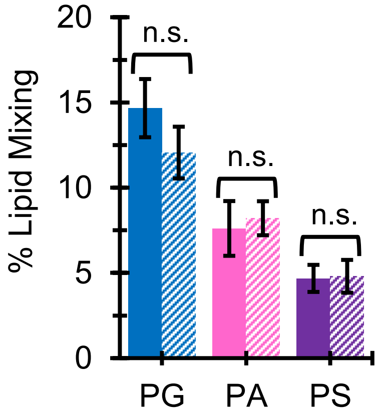

Figure S17. Tail saturation has no impact on LASV FD-initiated fusion. Lipids with a single unsaturated tail (POXX, solid color) and double unsaturated tail (DOXX, dashed color) did not have significant impact on the ability of the LASV FD to initiate fusion if the same head group moiety was present, i.e., PG (blue), PA (pink), and PS (purple) (n ≥ 9). Student's t–test assuming unequal variances used to calculate the *P*-value; n.s. = not significant.

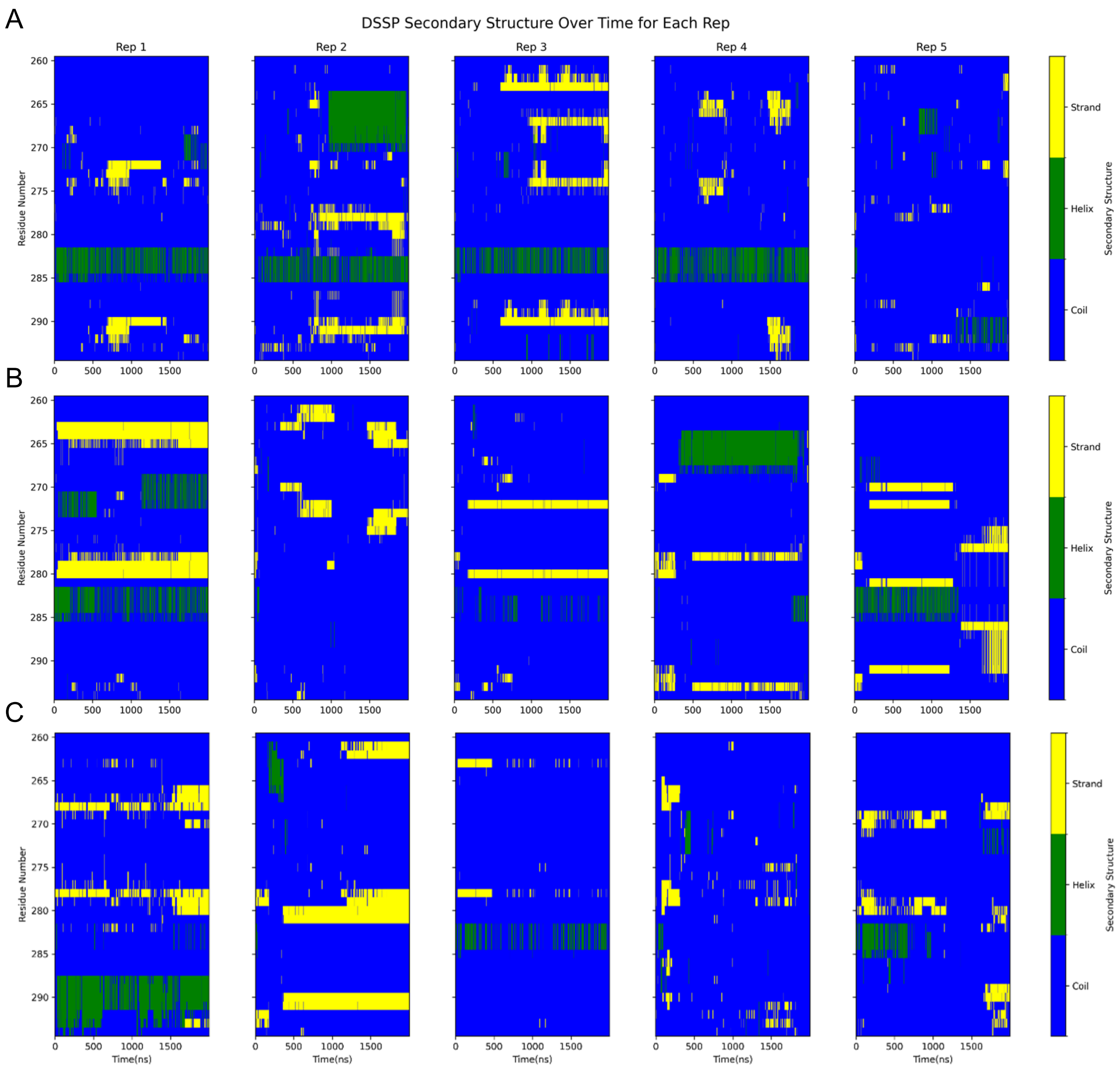

Figure S18. In MD simulations, the LASV FD can associate with membranes containing POPG and BMP, but not POPS. [A-C] Helical graph over the time course of the simulation for the different replicates indicates that the LASV FD has helical content in [A] POPC:POPG and [B] POPC:BMP, but relatively transient in [C] POPC:POPS.

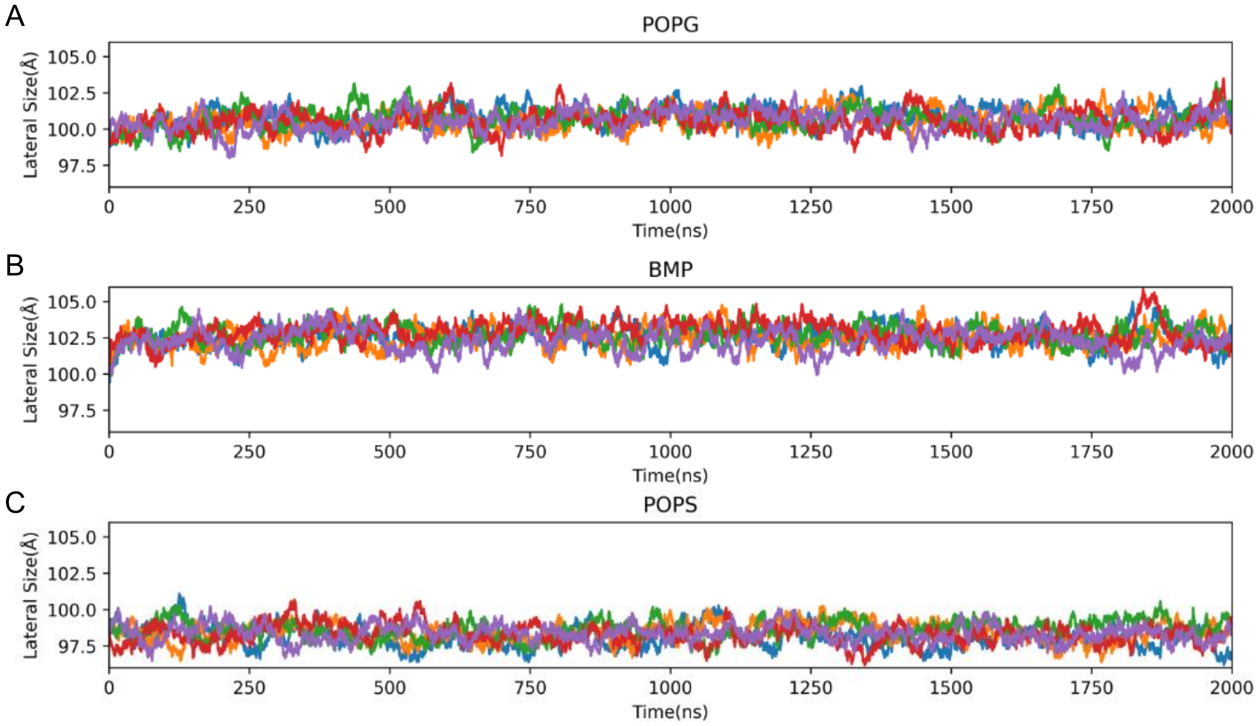

Figure S19. MD simulation showing the effect of the LASV FD on packing of different membrane environment. [A-C] shows how the lateral size of membrane changes over the time course in the presence of [A] POPC:POPG [B] POPC:BMP and [C] POPC:POPS.

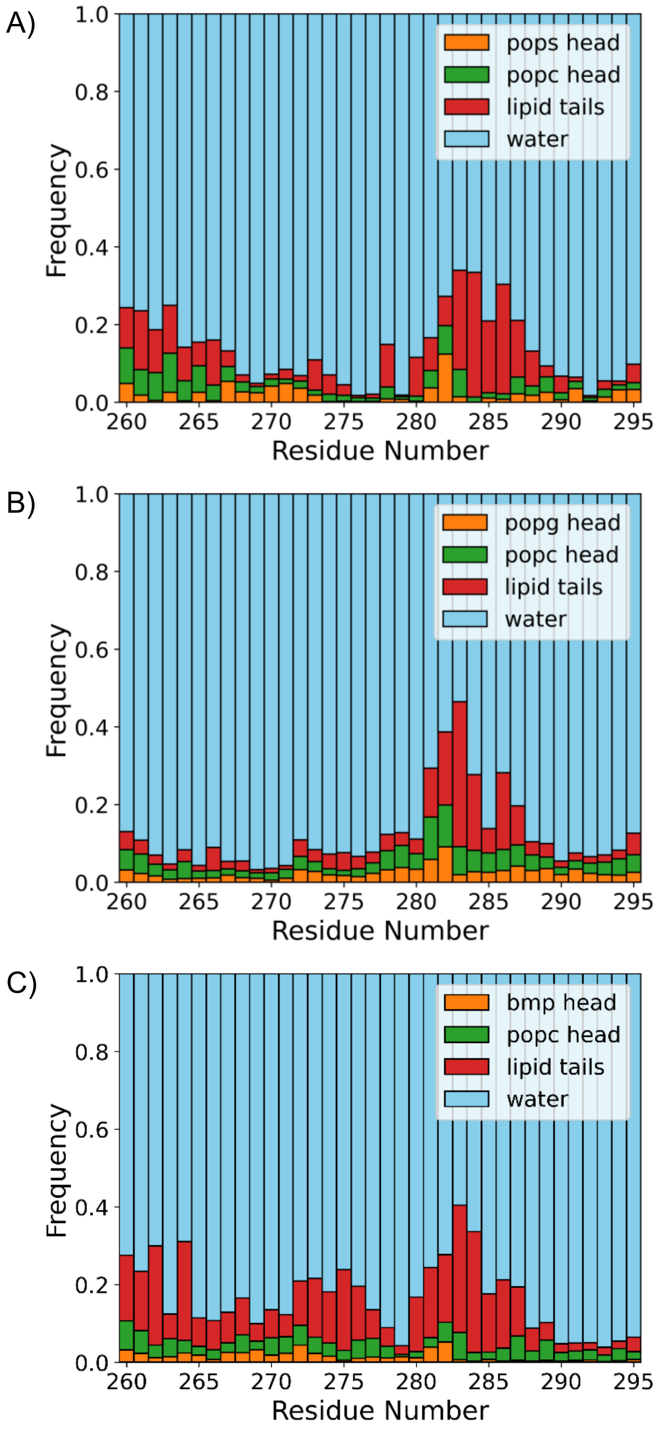

Figure S20. Contact analysis of LASV FD in different anionic lipid membranes. The frequency of contact is shown with water (blue), POPC head (green), head group of specific lipids (orange) [A] POPC:POPS, [B]POPC:POP, and [C] POPC:BMP, and lipid tails (red).

Table S1. Lipid composition of each membrane system utilized in molecular dynamics (MD) simulations.

Membrane System	POPC (16:0, 18:1 (9Z))	POPG (16:0, 18:1 (9Z))	BMP (18:1, 18:1)	POPS (16:0, 18:1 (9Z))	Total Lipid Count per Leaflet
POPC:POPG	104	56	0	0	160
POPC:BMP	104	0	56	0	160
POPC:POPS	104	0	0	56	160

Table S2. The LASV FD is largely a random coil in the pre-fusion state. A three-state backbone code was assigned to each residue via TALOS+ to relate the NMR chemical shifts and backbone torsion angles: A – alpha, -160 < Φ < 0 and -70 < Ψ < 60; P – positive, 0 < Φ < 160 and -60 < Ψ < 95; B – all others.

Residue	A	P	B	Φ (deg)	Ψ (deg)	Classification
G260	X	X	X	X	X	X
T261	0.3629	0.0445	0.5926	9999	9999	B
F262	0.02	0.0038	0.9762	-103.36	135.905	B
T263	0.0072	0.0012	0.9916	-124.138	135.929	B
W264	0.0091	0.002	0.9889	-140.552	160.013	B
T265	0.0026	0	0.9974	-90.04	142.794	B
L266	0.1901	0.0053	0.8046	-81.972	133.706	B
S267	0.1238	0.0067	0.8695	-87.453	131.924	B
D268	0.9132	0	0.0868	-62.05	-37.185	A
S269	0.9256	0.0081	0.0663	-66.001	-35.269	A
E270	0.7835	0.0149	0.2016	-99.847	2.97	A
G271	0.0891	0.7638	0.1471	87.45	5.133	P
K272	0.0459	0	0.9541	-77.97	145.786	B
D273	0.1483	0.0232	0.8285	-90.186	133.528	B
T274	0	0	1	-87.326	126.207	B
P275	0.1642	0.0092	0.8265	-56.491	139.354	B
G276	0.0379	0.9368	0.0253	71.27	13.882	P
G277	0.0665	0.7898	0.1437	87.597	2.132	P
Y278	0.0146	0	0.9854	-72.939	140.26	B
C279	0.5061	0.0253	0.4686	-69.064	-29.003	A
L280	0.9162	0.0138	0.07	-94.849	-20.5	A
T281	0.0559	0.0041	0.94	-140.896	125.101	B
R282	0.8469	0	0.1531	-76.105	-28.183	A
W283	0.7979	0.0154	0.1867	-72.422	-30.108	A
M284	0.8202	0.0143	0.1655	-71.043	-23.787	A
L285	0.1161	0.0255	0.8584	-75.178	130.323	B
I286	0.0266	0	0.9734	-97.401	137.845	B
E287	0.1494	0.0082	0.8423	-89.871	136.457	B
A288	0.9317	0.0077	0.0606	-70.208	-26.748	A
E289	0.2645	0.0476	0.688	-103.589	147.751	B
L290	0.0107	0.0056	0.9837	-81.757	136.902	B
K291	0.0281	0.0015	0.9704	-100.366	146.75	B
C292	0.0171	0	0.9829	-87.151	116.576	B
F293	0.3211	0.0255	0.6535	-104.298	141.561	B
G294	0	0.0031	0.9969	-127.736	162.394	B
N295	0.372	0.0498	0.5782	9999	9999	B

Table S3. Predicted dihedral angles suggest that the LASV FD has a helical structure within its FL in its post-fusion state. A three-state backbone code was assigned to each residue via TALOS+ to relate the NMR chemical shifts and backbone torsion angles: A – alpha, -160 < Φ < 0 and -70 < Ψ < 60; P – positive, 0 < Φ < 160 and -60 < Ψ < 95; B – all others. The residues likely to form a helix (black box) are located within the FL from R^{282} to L^{290}.

Residue	A	P	B	Φ (deg)	Ψ (deg)	Classification
G260	0.5068	0.0437	0.4495	9999	9999	N
T261	0.0235	0	0.9765	-96.604	138.83	B
F262	0.3067	0.013	0.6804	-101.298	129.364	B
T263	0.1028	0.0218	0.8755	-109.042	138.603	B
W264	0.0032	0.004	0.9928	-138.058	163.49	B
T265	0.0019	0	0.9981	-88.179	152.043	B
L266	0.5564	0	0.4436	-82.183	137.134	A
S267	0.4401	0.0407	0.5192	-66.043	-34.703	B
D268	0.9278	0.0036	0.0686	-68.762	-31.162	A
S269	0.9466	0.0073	0.0461	-65.386	-34.876	A
E270	0.9587	0	0.0413	-97.485	0.763	A
G271	0.0986	0.8361	0.0653	78.626	16.904	P
K272	0.1452	0.012	0.8428	-75.683	144.486	B
D273	0.169	0.0318	0.7993	-86.531	141.921	B
T274	0	0	1	-94.543	126.679	B
P275	0.259	0.0147	0.7264	-56.491	139.354	B
G276	0.0389	0.943	0.0181	68.861	18.746	P
G277	0.0694	0.8347	0.0959	86.458	1.243	P
Y278	0.0216	0	0.9784	-83.047	138.209	B
C279	0.6291	0.0277	0.3432	-80.975	115.565	A
L280	0.0176	0.0008	0.9816	-105.654	134.554	B
T281	0.1842	0.0049	0.8109	-86.045	142.567	B
R282	0.9033	0	0.0967	-69.85	-31.059	A
W283	0.9265	0.0195	0.0539	-72.727	-33.439	A
M284	0.9522	0.0014	0.0464	-66.117	-36.429	A
L285	0.987	0.0043	0.0088	-78.996	-30.413	A
I286	0.8864	0.0063	0.1073	-73.005	-27.414	A
E287	0.9854	0.0061	0.0085	-67.92	-37.857	A
A288	0.9877	0.0033	0.009	-64.826	-33.075	A
E289	0.9816	0.0047	0.0136	-79.945	-20.587	A
L290	0.6746	0.016	0.3094	-84.02	-26.751	A
K291	0.2883	0.0155	0.6962	-101.144	146.926	B
C292	0.2778	0.0155	0.7066	-72.498	-28.285	B
F293	0.9382	0.0141	0.0476	-100.604	-7.119	A
G294	0	0.0459	0.9541	-121.627	146.372	B
N295	0.3975	0.0624	0.5401	9999	9999	N

Table S4. Comparison of the average relative intensities of the LASV FD in different paramagnetic probes. In the pre-fusion state, both components of the FD have similar signals and are solvent-exposed. However, in the post-fusion state, the FP remains solvent-exposed, whereas the FL was inserted into the lipid head group of the membrane.

	**PRE-FUSION**	**POST-FUSION**		
	**Gd-DTPA**	**Gd-DTPA**	**5-DSA**	**16-DSA**
FD	0.420 ± 0.021	0.222 ± 0.026	0.439 ± 0.037	0.620 ± 0.027
FP	0.458 ± 0.027	0.126 ± 0.022	0.530 ± 0.068	0.623 ± 0.046
FL	0.431 ± 0.025	0.317 ± 0.038	0.353 ± 0.034	0.620 ± 0.039

Table S5. Comparison of the overall relaxation times of the LASV FD in the different fusion states. In the pre-fusion state, the FP was slightly more flexible than the FL with values supportive of a random coil conformation. The FP continued to be more flexible than the FL in the post-fusion state, but to a much greater extent, with the FL becoming restricted.

	**PRE-FUSION**			**POST-FUSION**		
	**$^{1}H - ^{15}N$ NOEs (s)**	**R_1 (s^{-1})**	**R_2 (s^{-1})**	**$^{1}H - ^{15}N$ NOEs (s)**	**R_1 (s^{-1})**	**R_2 (s^{-1})**
FD	0.448 ± 0.027	1.735 ± 0.091	4.941 ± 0.356	0.419 ± 0.038	1.305 ± 0.040	6.109 ± 0.683
FP	0.369 ± 0.018	1.750 ± 0.159	4.137 ± 0.550	0.253 ± 0.034	1.280 ± 0.036	4.426 ± 0.390
FL	0.497 ± 0.046	1.651 ± 0.062	5.708 ± 0.501	0.564 ± 0.048	1.339 ± 0.078	7.947 ± 1.224

Table S6. The percentage that a given conformer is populated is residue-dependent in both the pre- and post-fusion states. Notably, in the pre-fusion state, the signal for the b conformer of W^{264} and a conformer of D^{268} (bolded) overlap with each other and are not an accurate representation of the conformer populations. In turn, peak intensities in the backbone strips were used to justify the assignment of each population. Residues that do not occupy a given conformer are blacked out. The first (a), second (b), third (c), and fourth (d) most populated conformers of a given residue are shown.

		PRE-FUSION				POST-FUSION			
		a	b	c	d	a	b	c	d
FP	F262	53%	37%	10%		59%	26%	9%	6%
	T263	76%	13%	11%		45%	28%	27%	
	**W264**	**34%**	**56%**	**10%**		70%	13%	9%	8%
	T265	71%	29%			53%	29%	18%	
	L266	38%	25%	22%	15%	40%	24%	20%	16%
	S267	79%	21%			68%	32%		
	**D268**	**78%**	**22%**			100%			
	K272	91%	9%			88%	12%		
	T274	95%	5%			91%	9%		
	Y278	43%	27%	23%	7%	100%			
FL	C279	71%	29%			61%	39%		
	L285	77%	23%			100%			
	I286	47%	35%	18%		100%			
	E287	89%	11%			100%			
	A288	79%	21%			100%			
	E289	46%	32%	22%		83%	17%		
	L290	67%	67%	12%		100%			
	C292	100%				57%	43%		
	F293	100%				78%	22%		
	**Average**	**67 ± 19%**	**24 ± 12%**	**16 ± 6%**	**11 ± 6%**	**67 ± 17%**	**25 ± 11%**	**16 ± 8%**	**10 ± 6%**

Table S7. In the pre-fusion state, the different conformers of the LASV FD have similar predicted dihedral angles. A three-state backbone code was assigned to each residue via TALOS+ to relate the NMR chemical shifts and backbone torsion angles: A – alpha, $-160 < \Phi < 0$ and $-70 < \Psi < 60$; P – positive, $0 < \Phi < 160$ and $-60 < \Psi < 95$; B – all others. The most populated conformer (a) is the residue label, whereas the second (b), third (c), and fourth (d) most populated conformers are indicated as such.

Residue	A	P	B	Φ (deg)	Ψ (deg)	Classification
F262	0.0200	0.0038	0.9762	-103.360	135.905	B
F262b	0.4000	0.0072	0.9528	-99.874	146.588	B
F262c	0.0147	0.0016	0.9837	-107.236	141.816	B
T263	0.0072	0.0012	0.9916	-124.138	135.929	B
T263b	0.0057	0.0015	0.9928	-127.687	138.507	B
T263c	0.0233	0.0029	0.9738	-108.678	131.182	B
W264	0.0091	0.0020	0.9889	-140.552	160.013	B
W264b	0.0096	0.0021	0.9883	-140.353	158.993	B
W264c	0.0065	0.0023	0.9912	-132.191	159.132	B
T265	0.0026	0	0.9974	-90.040	142.794	B
T265b	0.0029	0	0.9971	-94.818	150.419	B
L266	0.1901	0.0053	0.8046	-81.972	133.706	B
L266b	0.2166	0.0018	0.7816	-81.972	133.706	B
L266c	0.1909	0.0036	0.8055	-81.972	133.706	B
L266d	0.1845	0.0027	0.8128	-81.972	133.706	B
S267	0.1238	0.0067	0.8695	-87.453	131.924	B
S267b	0.2735	0.0140	0.7124	-63.324	-37.756	B
D268	0.9132	0	0.0868	-62.050	-37.185	A
D268b	0.9444	0.0010	0.0546	-64.607	-35.129	A
K272	0.0459	0	0.9541	-77.970	145.786	B
K272b	0.0468	0	0.9532	-79.519	136.657	B
T274	0	0	1	-87.326	126.207	B
T274b	0	0	1	-115.991	127.654	B
Y278	0.0146	0	0.9854	-72.939	140.260	B
Y278b	0.0189	0	0.9811	-72.939	140.260	B
Y278c	0.0034	0	0.9966	-76.794	141.392	B
Y278d	0.0036	0	0.9966	-77.226	143.301	B
C279	0.5061	0.0253	0.4686	-69.064	-29.003	A
C279b	0.3114	0.0077	0.6808	-101.077	142.122	B
L285	0.1161	0.0255	0.8584	-75.178	130.323	B
L285b	0.4407	0.0586	0.5007	-69.516	126.270	B
I286	0.0266	0	0.9734	-97.401	137.845	B
I286b	0.0120	0.0056	0.9824	-77.489	131.803	B
I286c	0.0445	0.0009	0.9546	-96.844	150.896	B

TABLE S7 CONTINUED ON NEXT PAGE

TABLE S7 CONTINUED						
**Residue**	**A**	**P**	**B**	**Φ (deg)**	**Ψ (deg)**	**Classification**
E287	0.1494	0.0082	0.8423	-89.871	136.457	B
E287b	0.0429	0	0.9571	-90.715	144.977	B
A288	0.9317	0.0077	0.0606	-70.208	-26.748	A
A288b	0.9257	0.0110	0.0633	-75.261	-21.553	A
E289	0.2645	0.0476	0.688	-103.589	147.751	B
E289b	0.1203	0.0278	0.8519	-93.807	146.772	B
E289c	0.0893	0.0236	0.8871	-87.517	134.364	B
L290	0.0107	0.0056	0.9837	-81.757	136.902	B
L290b	0.0155	0.0038	0.9808	-80.729	134.134	B
L290c	0.0121	0.0045	0.9834	-85.355	130.667	B

Table S8. Predicted dihedral angles of the different conformers are relatively similar for the LASV FD in the post-fusion state. A three-state backbone code was assigned to each residue via TALOS+ to relate the NMR chemical shifts and backbone torsion angles: A – alpha, $-160 < \Phi < 0$ and $-70 < \Psi < 60$; P – positive, $0 < \Phi < 160$ and $-60 < \Psi < 95$; B – all others. The most populated conformer (a) is the residue label, whereas the second (b), third (c), and fourth (d) most populated conformers are indicated as such.

Residue	A	P	B	Φ (deg)	Ψ (deg)	Classification
F262	0.3067	0.0130	0.6804	-101.298	129.364	B
F262b	0.2659	0.0236	0.7105	-104.883	132.792	B
F262c	0.0752	0.0056	0.9191	-102.444	131.969	B
F262d	0.0666	0.0052	0.9282	-102.444	131.969	B
T263	0.1028	0.0218	0.8755	-109.042	138.603	B
T263b	0.0019	0.0049	0.9932	-123.795	140.850	B
T263c	0.0291	0.0049	0.9660	-111.131	141.192	B
W264	0.0091	0.0020	0.9889	-140.552	160.013	B
W264b	0.0095	0.0020	0.9885	-139.437	159.124	B
W264c	0.0067	0.0027	0.9906	-140.259	165.618	B
W264d	0.0069	0.0026	0.9905	-140.259	165.618	B
T265	0.0019	0	0.9981	-88.179	152.043	B
T265b	0.0016	0	0.9984	-92.295	154.232	B
T265c	0.0023	0	0.9977	-94.363	154.151	B
L266	0.5564	0	0.4436	-82.183	137.134	A
L266b	0.3562	0	0.6438	-82.183	137.134	B
L266c	0.8594	0	0.1406	-68.489	-24.282	A
L266d	0.8645	0	0.1355	-68.489	-24.282	A
S267	0.4401	0.0407	0.5192	-66.043	-34.703	B
S267b	0.6084	0.0075	0.3841	-64.255	-33.783	A
K272	0.1452	0.0120	0.8428	-75.683	144.486	B
K272b	0.1128	0.0049	0.8823	-74.603	134.747	B
T274	0	0	1	-94.543	126.679	B
T274b	0	0	1	-119.516	126.546	B
C279	0.6291	0.0277	0.3432	-80.975	115.565	A
C279b	0.5678	0.0284	0.4038	-75.107	122.862	A
E289	0.9816	0.0047	0.0136	-79.945	-20.587	A
E289b	0.9869	0.0046	0.0085	-79.557	-19.575	A
C292	0.2778	0.0155	0.7066	-72.498	-28.285	B
C292b	0.2577	0.0108	0.7315	-72.498	-28.285	B
F293	0.9382	0.0141	0.0476	-100.604	-7.119	A
F293b	0.9430	0.0135	0.0436	-104.646	-3.126	A
